# Immunostimulatory gene therapy with chemotherapy remodels pancreatic tumors and induces immune signatures associated with prolonged survival

**DOI:** 10.1136/jitc-2026-016908

**Published:** 2026-10-28

**Authors:** Jessica Wenthe, Hanna Grauers Wiktorin, Amanda Hahn, Sedigheh Naseri, Ahmed Calandigary, Luke White, Linda C Sandin, Clara Bernedal Nordström, Stephan Mielke, Tanja Lövgren, Gustav J Ullenhag, Angelica Loskog

**Affiliations:** 1 Immunology, Genetics and Pathology, Uppsala University, Uppsala, Sweden; 2 Department of Oncology, Uppsala University Hospital, Uppsala, Sweden; 3 Lokon Pharma AB, Uppsala, Sweden; 4 Department of Cellular Therapy and Allogeneic Stem Cell Transplantation (CAST), Karolinska Institutet, Stockholm, Sweden

**Keywords:** Gene therapy, Tumor microenvironment - TME, Immunotherapy, Oncolytic virus

## Abstract

**Background:**

Immune desert/excluded tumors remain a major immunotherapeutic challenge. Herein, we aim to describe mechanistic insights of LOAd703, a novel tumor microenvironment gene engineering therapy targeting CD40 and 4–1BB, in patients with advanced solid cancer commonly associated with unresponsiveness to immunotherapy.

**Methods:**

The study evaluated blood and biopsy samples from pancreatic (n=29), colorectal (n=5), biliary (n=3), and ovarian cancer (n=4), treated with LOAd703 in combination with chemotherapy in a Swedish phase I/II clinical trial (NCT032259899).

**Results:**

Transcriptomic profiling of tumors before and after treatment initiation revealed induction of signatures known to predict immunotherapy responses and an enrichment for adaptive immune response pathways encompassing antigen-presentation and T-cell activation. In pancreatic cancer subanalysis, adaptive immune responses together with increased CD40 expression were associated with longer survival and CD40 appeared as a central node among upregulated immune biomarkers consistent with the mechanism of action of LOAd703. Treatment benefit was further associated with reduced expression of immunosuppressive and tumor-promoting factors both in tumors and peripheral blood. Systemically, treatment generated a Th1 profile in plasma, accompanied by an increase in effector/effector memory T cells and a reduction of regulatory T cells.

**Conclusions:**

These findings support that intratumoral LOAd703 in combination with chemotherapy drives local and systemic immune remodeling in commonly immunotherapy-unresponsive tumors. Further, the treatment reprograms the tumor microenvironment to reduce tumor-promoting factors and immunosuppression.

**One sentence summary:**

Targeting CD40 and 4–1BB with gene therapy in combination with chemotherapy induces immune responses associated with longer survival in pancreatic cancer.

## Graphical abstract

**Graphical abstract:**
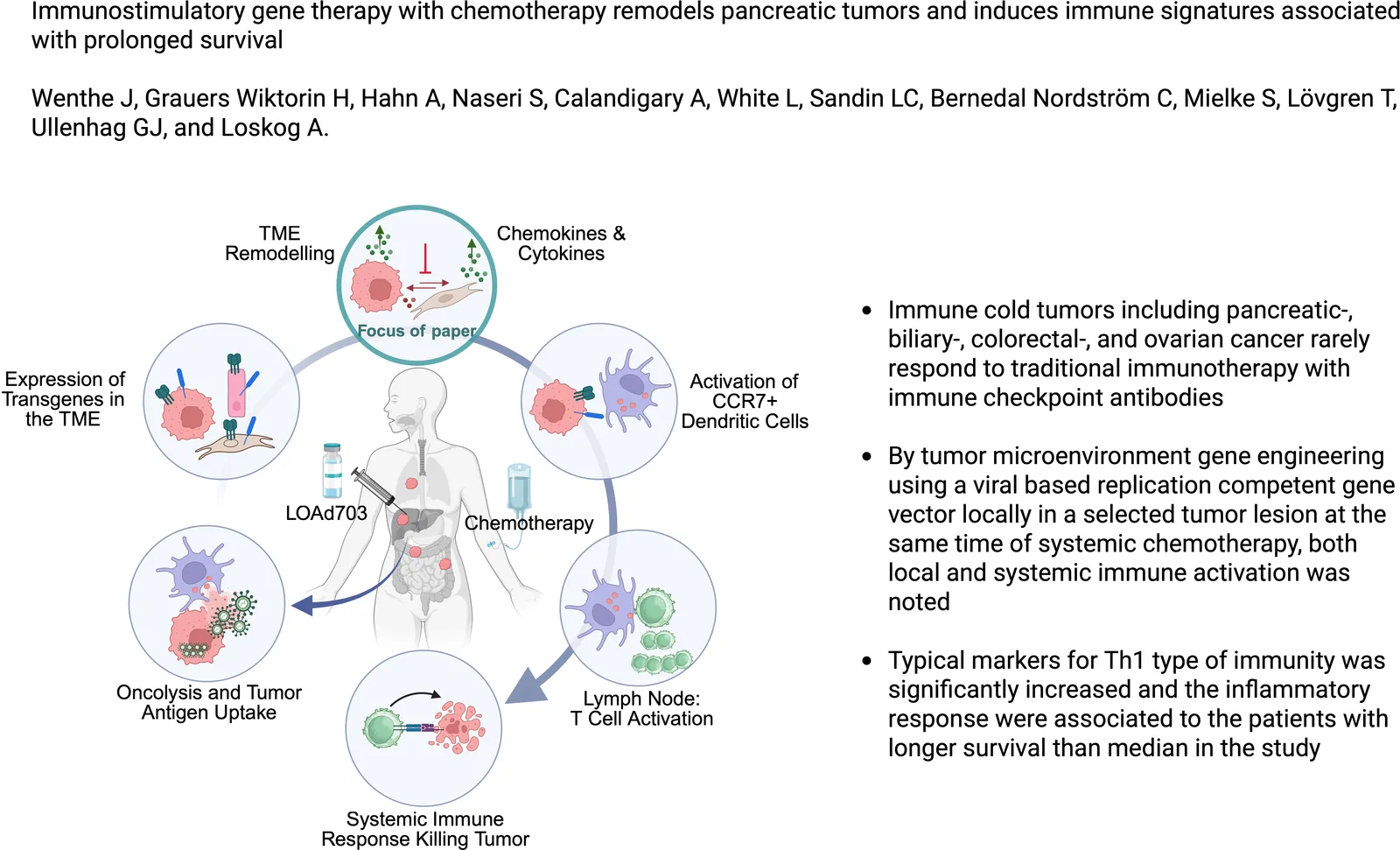

**WHAT IS ALREADY KNOWN ON THIS TOPIC**

- The tumor microenvironment (TME) of so-called immune cold cancers such as pancreatic, biliary, colorectal, and ovarian cancer, is characterized by dense stroma, few infiltrating T cells, and a predominance of immunosuppressive myeloid cells and fibroblasts producing protumorigenic factors that collectively hinder antitumor immune responses and confer resistance to immune checkpoint inhibitors (ICI). Virus-based gene vehicles such as oncolytic viruses (OV) armed with immunostimulatory genes can resensitize ICI-refractory melanoma to continued ICI treatment.

**WHAT THIS STUDY ADDS**

- LOAd703 is both a gene therapy and an OV as it expresses immunostimulatory transgenes (TMZ-CD40L and 4–1 BBL) in both the cells of the TME and the tumor cells. The immunostimulatory genes were selected to drive activation of antigen presentation by activating dendritic cells with subsequent activation of T cells. Further, TMZ-CD40L can redirect M2 to M1 differentiation of myeloid cells. In this study, it is demonstrated that LOAd703 together with chemotherapy can spark activation of Th1-type immune responses both locally in the TME and systemically in blood, in the category of immune cold tumors.

**HOW THIS STUDY MIGHT AFFECT RESEARCH, PRACTICE OR POLICY**

- The presented data may increase the interest of off-the-shelf viral vectors for TME gene engineering simultaneously to providing virus-mediated oncolysis which uniquely uses the tumor itself as a source of antigen at the same time of providing a local Th1 adjuvant to spark antitumor immunity and thereby sensitize patients with so far ICI-resistant cancers.

## Background

 The introduction of immunotherapy, that is, immune checkpoint inhibitors which facilitate tumor-specific T-cell responses by releasing inhibitory brakes, has revolutionized the treatment of patients with cancer. However, this approach is largely ineffective in patients who present with so-called immune excluded or desert tumor phenotypes characterized by few functional T cells in the tumor microenvironment (TME).^1^ Pancreatic cancer is a notoriously difficult-to-treat tumor with a TME dominated by desmoplasia and high infiltration of myeloid cells, conditions that create an immunosuppressive environment and severely limit T-cell entry and activation.^2 3^ Similarly, the TME of other cancers with limited responsiveness to immune checkpoint inhibitors, such as biliary, colorectal, and ovarian cancer, is frequently characterized by dense stroma, lack of immune cell infiltration, and a predominance of immunosuppressive cells and protumorigenic factors that collectively hinder antitumor immune responses.^4–6^ Hence, the advancement of innovative therapeutic strategies capable of remodeling the immunosuppressive TME and promoting robust antitumor immune responses represents an urgent clinical need. To answer this unmet need, the TME gene engineering therapy LOAd703 (delolimogene mupadenorepvec), which uses an adenoviral replication-competent gene vehicle that encodes trimerized membrane-bound CD40L (TMZ-CD40L) and 4–1 BBL, was developed. Expression of these potent immunostimulatory molecules is induced across all infected cells within the TME, facilitating strong immune activation. However, direct cytolytic effects are strictly confined to tumor cells due to restrictive viral replication.^7^ The transgenes target central pathways in antitumor immunity. CD40/CD40L signaling is essential for adequate activation of dendritic cells (DCs) and consequently priming of potent tumor-specific T-cell responses, but is also involved in B-cell immunity, endothelial cell activation, and reduction of protumorigenic factors produced by fibroblasts/stellate cells.^7–9^ Additionally, engagement of CD40 expressed by tumor cells can mediate tumor cell apoptosis.^10^ Interaction of 4–1 BBL with its receptor 4–1BB on T cells and natural killer (NK) cells promotes survival signals and enhanced effector functions.^11^ Preclinical studies have confirmed that LOAd703 alters immunosuppressive components of the TME, matures DCs with subsequent activation of antigen-specific T-cell responses, expands NK cells, sensitizes treatment-resistant tumors to immune checkpoint inhibitors, and lyses multiple different tumor cell types.^7 12–16^

Clinically, LOAd703 has been evaluated in combination with nab-paclitaxel and gemcitabine±atezolizumab in patients with pancreatic cancer and in combination with atezolizumab in patients with melanoma.^17 18^ In the Phase I/II LOKON002 trial, LOAd703 was evaluated in patients with advanced solid cancers (pancreatic, ovarian, colorectal or biliary cancer) in combination with gemcitabine-based chemotherapy.^19^ Herein, we conducted transcriptomic and proteomic/secretomic analysis on tumor biopsies and peripheral blood samples from patients treated in the LOKON002 trial to elucidate the potential of intratumoral LOAd703 TME gene engineering therapy to induce inflammatory responses both locally and systemically during ongoing chemotherapy in patients commonly unresponsive to immunotherapy.

## Methods

### Study design

Blood and tumor biopsies from patients participating in the LOKON002 (NCT03225989) phase I/II clinical trial were analyzed in this study. LOKON002 was an open-label, single-arm, bi-center study (Uppsala University Hospital and Karolinska University Hospital).^19^ In brief, 41 patients were dosed (pancreatic cancer, n=29; colorectal cancer, n=5; ovarian cancer, n=4; biliary cancer, n=3) to receive a maximum of eight biweekly intratumoral injections of LOAd703 in combination with a gemcitabine-based regimen as part of their standard-of-care treatment, or with gemcitabine as an immune-conditioning treatment if no standard options were available (ie, last line). Blood samples were collected at baseline (week 1) and at multiple time points post-treatment initiation. Tumor biopsies were collected at baseline (week 1) and at week 13 post-treatment initiation from the LOAd703 injection site. Effect was measured as overall survival (OS) and tumor response such as complete response (CR), partial response (PR), stable disease (SD), and progressive disease (PD) according to Response Evaluation Criteria for Solid Tumors version 1.1 (RECIST V.1.1).

### Gene expression analysis

Messenger RNA (mRNA) was purified from tumor biopsies (n=20) using the RNeasy Mini Kit and QIAshredder spin columns (QIAGEN, Hilden, Germany) according to manufacturer’s instructions. Gene expression was analyzed with nCounter PanCancer Immune Profiling Panel (NanoString Technologies, Seattle, Washington, USA) at the Clinical Genomics facility in Uppsala, Sweden. Data were normalized using the RUV-4 method in RStudio for calculating the fold change between week 13 and baseline. Approximated individual values were calculated by removing estimated unwanted factors calculated during the RUV-4 normalization from the observed log_2_ gene counts.

### Flow cytometry analysis

Peripheral blood mononuclear cells (PBMCs) were collected and analyzed at baseline (week 1; n=39), week 7 (n=39), week 13 (n=29), and at week 17 (n=21). PBMCs were washed in phosphate-buffered saline supplemented with 3 mM EDTA (Thermo Fisher Scientific) and 0.5% bovine serum albumin (Sigma-Aldrich, St. Louis, Missouri, USA) (staining buffer) and stained with fluorescent-labeled antibodies for 15 min at room temperature. The following antibodies and viability marker were used: 7-AAD (RRID:AB_2869266), anti-CD11b (RRID:AB_2868644), anti-CD127 (RRID:AB_2033938), anti-CD14 (RRID:AB_1645464), anti-CD16 (RRID:AB_2868628), anti-CD163 (RRID:AB_396296), anti-CD197/CCR7 (RRID:AB_10561679), anti-CD25 (RRID:AB_2783790), anti-CD3 (RRID:AB_2783791), anti-CD4 (RRID:AB_1645732) and (RRID:AB_396897), anti-CD45 (RRID:AB_2732010), anti-CD45RA (RRID:AB_1727497), anti-CD56 (RRID:AB_2868831), anti-CD8 (RRID:AB_2868803), anti-PD-1 (CD279) (RRID:AB_2033989), anti-ICOS (CD278) (RRID:AB_396878). All antibodies were purchased from BD Biosciences (San Jose, California, USA). After staining, cells were washed with staining buffer and analyzed with BD FACSCanto 2 (BD Biosciences) and FlowJo software (FlowJo, Ashland, Oregon, USA). T-cell phenotype analysis was performed with FlowJo plugins (FlowAI, DownSample, UMAP, FlowSOM).

### Proteomics

Plasma samples were analyzed at baseline (week 1; n=39), week 7 (n=39), week 13 (n=27), and at week 17 (n=21) with Olink Proteomics (Olink Target 96 Immuno-oncology and Oncology II panel) at Olink Proteomics service facility or TATAA Biocenter. The normalized protein expression is presented in log_2_.

### Statistical analysis

Alterations in gene/protein expression in the tumor/plasma post treatment were determined with correction for multiple comparisons by the false discovery rate (5%) Benjamini-Hochberg method. Differential expression between survival/response groups was calculated with multiple unpaired t-tests. Repeated measures analysis of variance (ANOVA) analysis with mixed effects model followed by Dunnett’s multiple comparison test was used for statistical comparison of expression data over time. For comparison of response groups over time, two-way ANOVA analysis with mixed effects models followed by Tukey’s multiple comparison test was used. The mixed models are preferred when samples from a patient may be lacking at certain time points as they make use of the full data set while the ANOVA test will ignore the incomplete patients’ data set.

### Procedure for managing conflicts of interest

Of the 12 coauthors, 5 reported conflicting interests in regard to the data in the manuscript. To manage conflicting interests, the data were handled with transparency to coauthors and are also presented in online supplemental information in the form of tables. Both authors with possible conflicts and without read and approved of the final manuscript and its figures and tables. A data availability statement is available with information on how to obtain data.

## Results

### Activation of an adaptive immune response in LOAd703 injected tumors

To evaluate the ability of LOAd703, in combination with chemotherapy, to revert TME-associated immunosuppression, tumor biopsies at baseline and at week 13 following six LOAd703 treatments in combination with chemotherapy were evaluated with NanoString analysis for gene expression. Figure 1a and online supplemental table 1 display all genes that were significantly increased at least twofold in the tumors post treatment. The genes and respective encoded proteins were explored in a functional interaction network and Gene Ontology (GO) enrichment analysis (STRING database^20^) (figure 1b,c). LOAd703 plus chemotherapy induced a broad intratumoral inflammatory response, marked by an adaptive immune response signature and high enrichment of antigen processing and presentation pathways along with both T-cell and B-cell signatures that included their key transcription factor interferon regulatory factor-4, which can be induced by CD40 signaling in B cells. In addition, elevated expression of cytotoxic effector molecules indicated an ongoing tumor cell lysis. A tumor inflammation signature (TIS) has been identified as a validated predictor of responses to checkpoint inhibition across different cancer indications.^21^ Biomarkers selected from the TIS were significantly induced by LOAd703 plus chemotherapy (figure 1d, harmonic p value=6.46E–07). Correspondingly, the treatment also significantly promoted the T effector/interferon gamma (IFN-γ) gene signature (figure 1e, harmonic p value=6.01E−06), previously correlated with responses to anti-programmed death-ligand 1 (PD-L1) therapy.^22^ Multiple checkpoint molecules were upregulated after treatment initiation, with the highest increase seen for CD96, IDO1, CD274 (PD-L1) and HAVCR2 (TIM-3). In contrast, the T cell immunoreceptor with Ig and ITIM domains (TIGIT) ligand Poliovirus receptor (PVR, CD155) and the NK cell inhibitory receptor CEACAM1 were downregulated (figure 1f).

**Figure 1:**
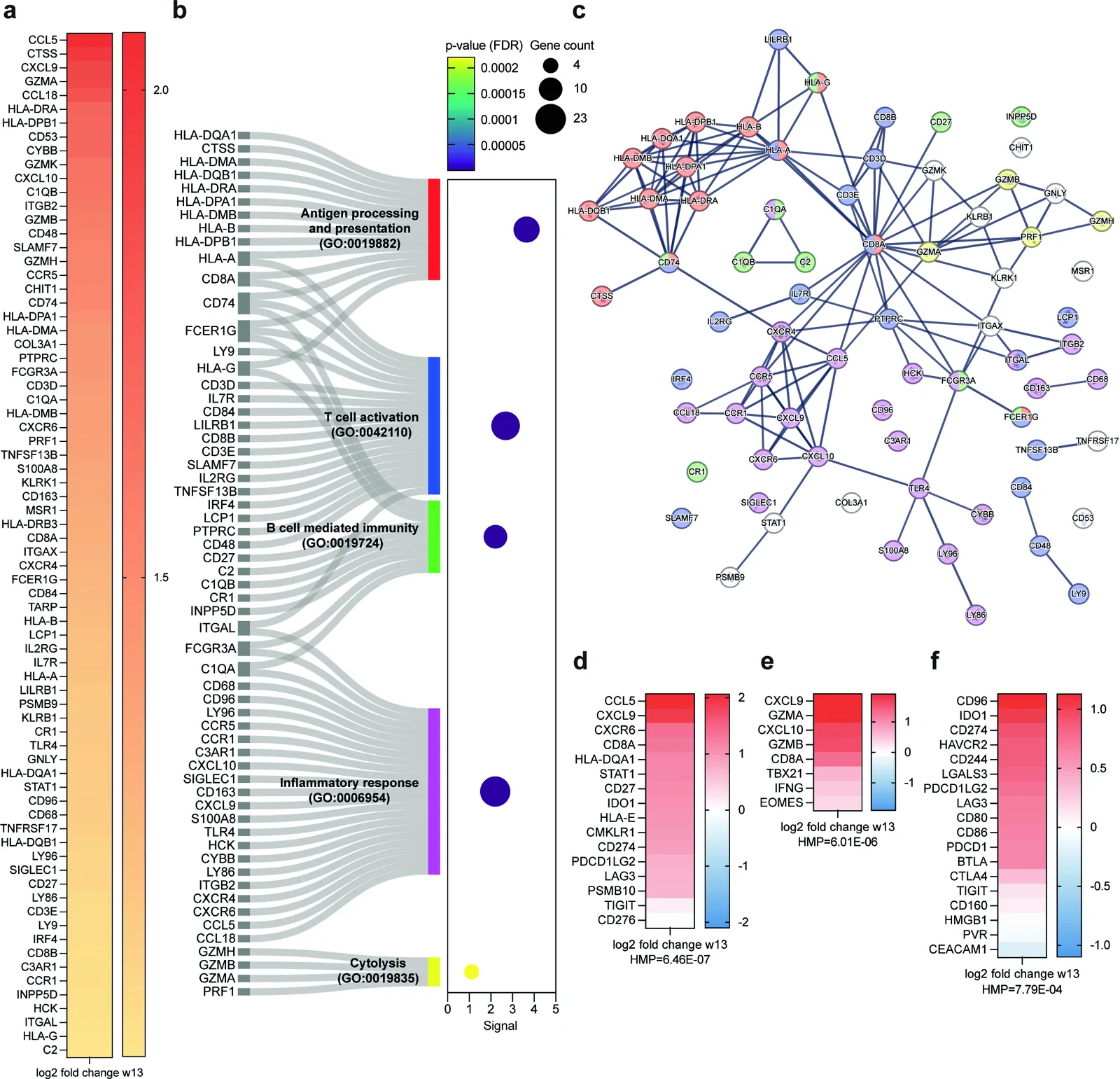
Biomarker profiling of the tumor microenvironment. Tumor biopsies of all patients with available paired pre/post-treatment biopsies (n=20; pancreatic cancer n=14; colorectal cancer n=2; ovarian cancer n=3; biliary cancer n=1) were analyzed with nCounter PanCancer Immune Profiling Panel (NanoString Technologies) at baseline and w 13 to evaluate mRNA biomarkers. Patients lacked paired samples due to progression (n=11) or withdrawn consent to biopsy (n=10). The data were normalized with RUV-4 method in RStudio and significantly changed expression at w13 was determined with correction for multiple comparisons by the FDR (5%) Benjamini-Hochberg method. The heatmap displays the log_2_ fold change of all genes that were upregulated by at least twofold at w13 (**a**) and the interaction of the encoded proteins are displayed in GO pathways (**b**) and in a functional association network (STRING) (**c**). The nodes are colored based on (**b**), which are displayed according to their signal (weighted harmonic mean between the ratio of observed versus expected genes in the respective pathway and the FDR. FDR adjusted p values are shown as color gradient and the observed gene counts are estimated by symbol size. In **d–f**, heatmaps display the log_2_ fold change of the respective genes in selected gene signatures with red and blue color indicating an upregulation and downregulation, respectively (d: tumor inflammation signature, e: T effector and IFN-γ signature, f: immune checkpoint molecules). For the gene signatures, a harmonic p value (HMP) was calculated. FDR, false discovery rate; GO, Gene Ontology; IFN, interferon; mRNA, messenger RNA; w, week. HMP, harmonic p value.

### Effector memory T cells were increased systemically and associated with response

Intratumoral injections of LOAd703 plus systemic chemotherapy triggered a multitude of inflammatory pathways in the tumors, yet achieving durable clinical responses likely requires systemic immune engagement. Peripheral blood mononuclear cells were characterized over time with flow cytometry, and the gating strategy is shown in online supplemental figure S1. Monocytes (figure 2a) were increased after treatment initiation, while CD163+myeloid cells decreased at week 7 (figure 2b). NK cells were also reduced, while T cells initially increased at week 7 and then subsequently decreased at week 17 (figure 2c,d). Most T cells were CD4+T cells, but the proportion of CD8+T cells was increasing over time (figure 2e). Importantly, a continuous shift towards a higher CD8+/ T regulatory ratio occurred, driven by a progressive decline in regulatory T cells (Tregs) (figure 2f). Immune checkpoint programmed cell death protein 1 (PD-1) was upregulated on both CD4+ and CD8+ T cells (figure 2g) alongside increased expression of activation marker ICOS predominantly on CD4+T cells (figure 2h). T cells were further characterized by their memory phenotype. A clustering analysis based on CD4, CD8, CD45RA and CCR7 expression identified seven distinct T-cell clusters (figure 2i). The effector memory (EM, Cluster 1: CD45RA−CCR7−) and effector (Cluster 2: CD45RA+CCR7−) phenotype were two main CD8+cell clusters expanding after treatment initiation. A small fraction of T cells was CD8+naïve (Cluster 3: CD45RA+CCR7+) and this phenotype further decreased over time. CD4+T cells were mainly of an EM (Cluster 4) or a naïve (Cluster 6) phenotype. The smallest CD4+fraction displayed a central memory (CM, Cluster 7: CD45RA−CCR7+) phenotype. Next, we evaluated the association between clinical responses observed in patients and T-cell phenotypes. Notably, the patients in the study experiencing partial response as best response had higher levels of both CD8+ and CD4+ EM cells at all measured time points, which indicates an association of systemic EM T-cell expansion with therapeutic efficacy (figure 2j,k).

**Figure 2:**
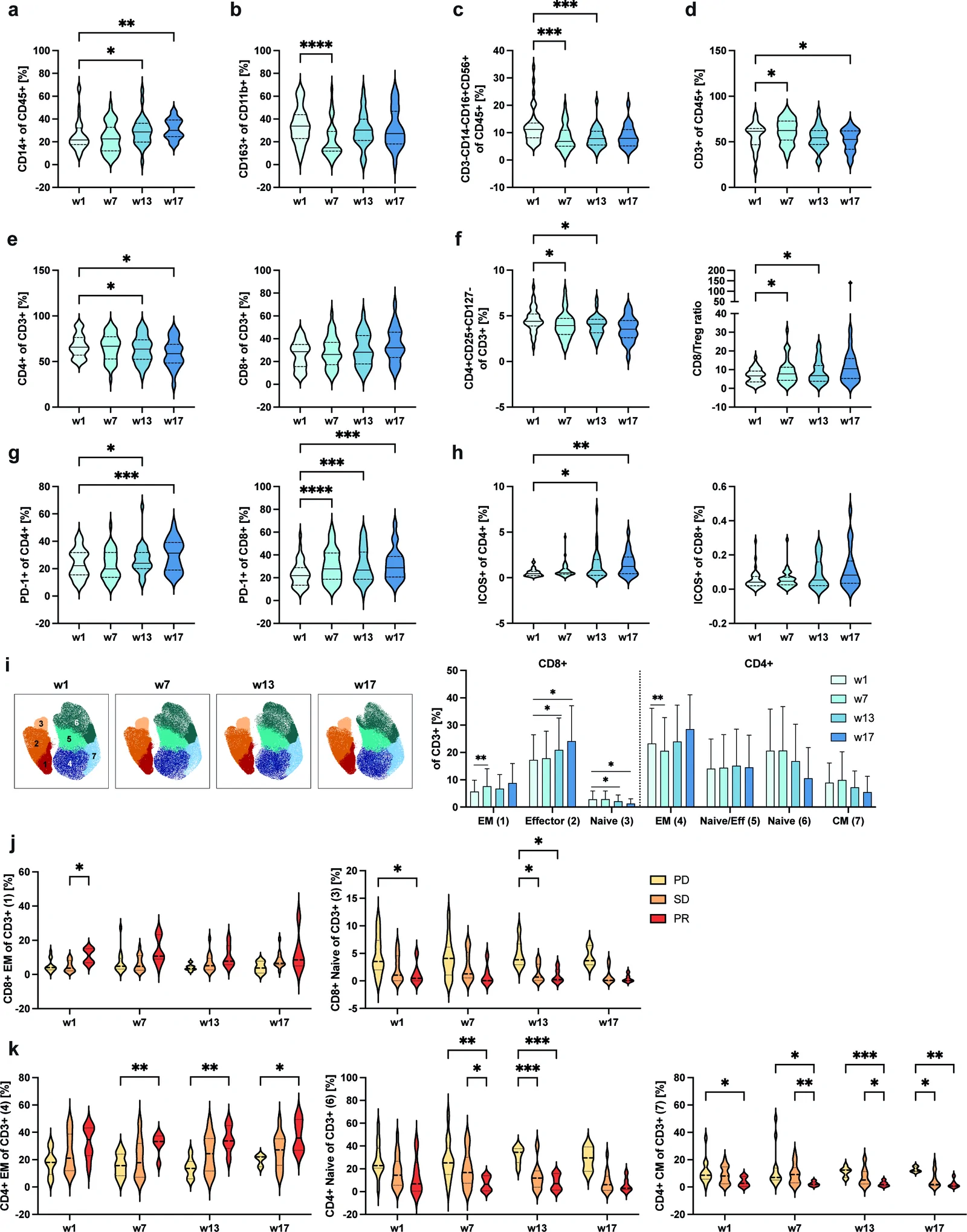
Immune cell phenotype analysis in blood. PBMCs were collected and analyzed with flow cytometry at baseline (w 1; n=39; pancreatic n=27; colorectal n=5; ovarian n=4; biliary n=3), w7 (n=39, same as w1), w13 (n=29; pancreatic n=21; colorectal n=2; ovarian n=4, biliary n=2), and at w17 (n=21; pancreatic n=15; colorectal n=2; ovarian n=3, biliary n=1). Drop out of samples over time were due to progression. The data are shown in violin plots with lines representing median and lower and upper quartiles at different time points (**a–h**) and in relation to best response (**j, k**): PR n=6 (w1, 7, 13, 17), SD n=18 (w1, 7), n=16 (w13), n=13 (w17), PD n=13 (w1, 7), n=7 (w13), n=3 (w17). Clustering analysis of T-cell phenotypes based on their expression of CCR7 and CD45RA were done with FlowJo Plugins UMAP and FlowSOM and identified clusters 1–7 are shown in I as UMAPs and bar graphs. Statistical calculations were performed by Mixed-effects models using either Dunnett’s (**a–i**) or Tukey’s (**j, k**) multiple comparison test (*p≤0.05, **p≤0.01, ***p≤0.001, ****p≤0.0001). CM, central memory; EM, effector memory; PBMC, peripheral blood mononuclear cells; PD, progressive disease; PR, partial response; SD, stable disease; w, week; UMAP, uniform manifold approximation and projection.

### Induction of a robust Th1-dominant immune profile in plasma

To further profile the systemic immune response, a proteomics analysis in blood was performed. Figure 3a displays the fold change of the investigated proteins at week 7 compared with baseline. Significantly changed proteins (online supplemental table 2) are shown in a heatmap (figure 3b). Most proteins were significantly upregulated and the top 10 upregulated proteins are further displayed over time, demonstrating a durable upregulation during the treatment period (figure 3c). These proteins were dominated by a Th1-associated cytokine profile with IFN-γ and its related cytokines (interleukin (IL)-18, C-X-C motif chemokine ligand (CXCL)9, CXCL10), DC-related proteins (lysosome associated membrane protein 3 [LAMP3], IL-15), and T-cell chemoattractant CX3CL1. Immunosuppressive mediators, such as the cell stress-induced heme-oxygenase-1, immune checkpoint ligand Gal-9, and Th2-related cytokine IL-6 were also increased. Only a few proteins were significantly downregulated. These included cytokines (IL-12, C-C motif chemokine ligand 17 [CCL17], CXCL5), factors involved in angiogenesis and tissue remodeling platelet-derived growth factor subunit b (PDGFB), angiopoietin 1 (ANGPT1), matrix metallo proteinas (MMP)12, MMP7, immune receptors/ligands (CD40L, FASLG, NCR1), and epidermal growth factor (EGF) (figure 3a,b).

**Figure 3:**
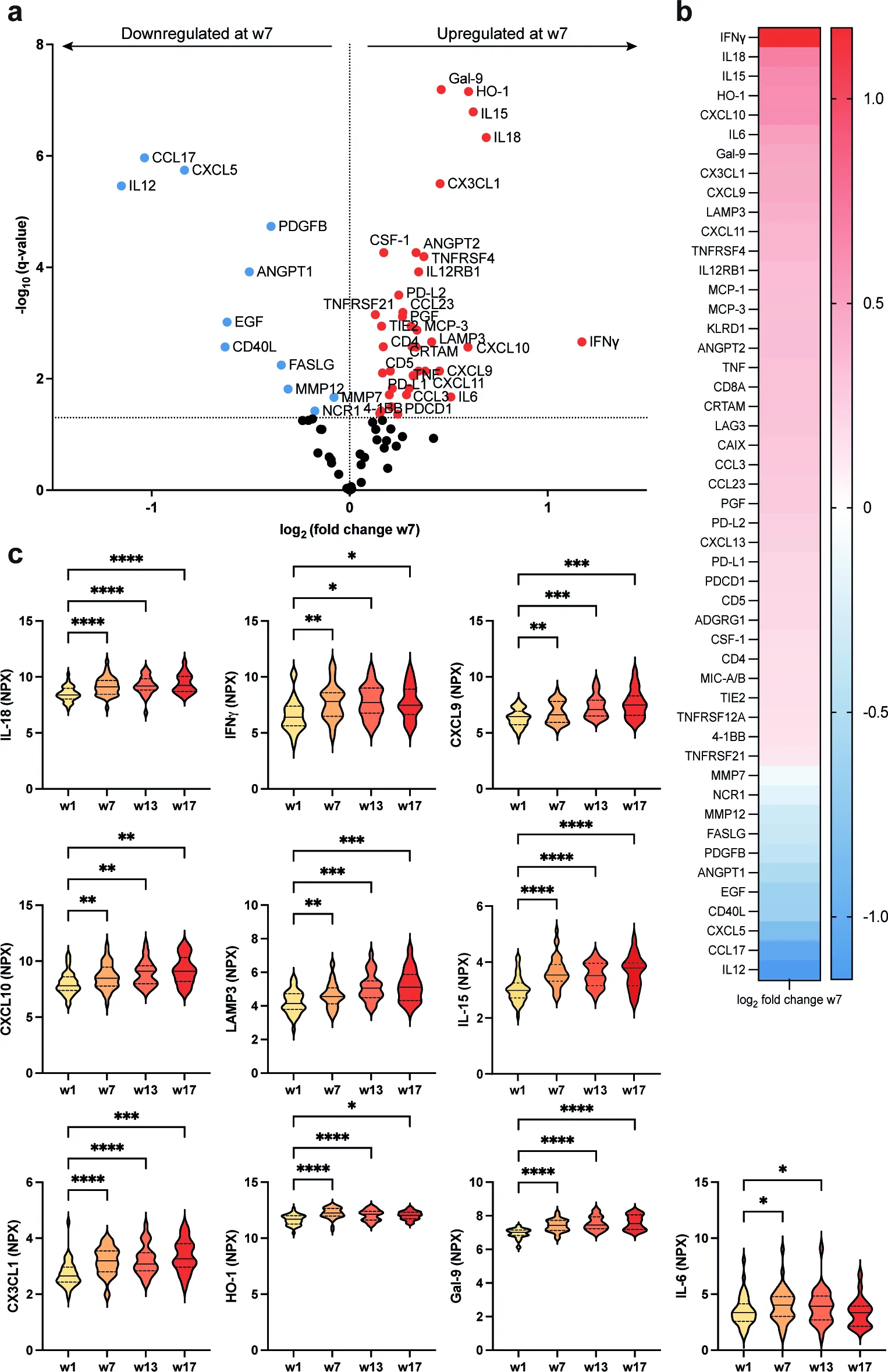
Immunological plasma biomarkers. Plasma samples from patients in the LOKON002 clinical trial were analyzed with Olink Target 96 Immuno-oncology panel. The volcano plot shows the log_2_ fold change of plasma proteins at w 7 compared with baseline (w1) (x-axis) (**a**). Statistically significant differences were determined with multiple paired t-tests followed by the FDR method of Benjamini and Hochberg (FDR=5%) and negative logarithm of adjusted p values are calculated and displayed as q value in the y-axis. The horizontal and vertical dotted lines represent the cut-off for significant q values and for upregulation versus downregulation, respectively. The heatmap shows the significantly changed proteins at w7 (**b**). In “a” and “b” red and blue colors represent upregulation and downregulation, respectively. Violin plots with lines representing median and lower and upper quartiles show the expression over time of the top 10 upregulated genes identified at w7 (**c**). The colors represent the different sample time points stated in the x-axis. Statistical differences in “c” were determined by mixed-effects model followed by Dunnett’s multiple comparison test (*p≤0.05, **p≤0.01, ***p≤0.001, ****p≤0.0001). Samples analyzed at baseline (w 1; n=39; pancreatic n=27; colorectal n=5; ovarian n=4; biliary n=3), w7 (n=39, same as w 1), w13 (n=29; pancreatic n=21; colorectal n=2; ovarian n=4; biliary n=2), and at w17 (n=21; pancreatic n=15; colorectal n=2; ovarian n=3; biliary n=1). Drop out of samples over time was due to progression. FDR, false discovery rate; IFN, interferon; IL, interleukin; w, week.

### Factors promoting cancer progression and metastasis were reduced

Next, we explored if LOAd703 triggers reduction of biomarkers of tumor growth, progression, and metastasis as seen in preclinical in vitro models.^7^ While most significantly changed proteins (online supplemental table 3) tested in the oncology panel increased following treatment, nine factors implicated in tumor growth and unfavorable outcomes showed notable reductions in plasma. These were growth factors EGF and stem cell factor (SCF), the endothelial growth factor receptor (EGFR) regulator protein secretory carrier membrane protein 3, Cathepsin V gene (CTSV) encoding for cathepsin L2, LYPD3, and the integrins integrin beta 5 (ITGB5) and integrin subunit alpha V (ITGAV, CD51), as well as the immune receptors CD207 and FASLG (figure 4a,b). To investigate further longitudinal changes of these proteins of interest, the expression levels were analyzed over time, showing a decrease only at week 7 and 13 for most, while SCF and CTSV maintained reduced levels until week 17 (figure 4c). Although several oncology panel proteins were upregulated at week 7, differential expression between patients surviving above versus below median OS (7.5 months) revealed significantly lower expression levels of PVRL4 encoding for nectin-4, vascular endothelial growth factor receptor-3, CD70, SYND1 encoding for Syndecan-1, WNT1-inducible-signaling pathway protein 1 (WISP-1), and the apoptosis inhibitor TNFRSF6B in longer surviving patients and a similar trend for the remaining overall upregulated markers (figure 4d, online supplemental table 4).

**Figure 4:**
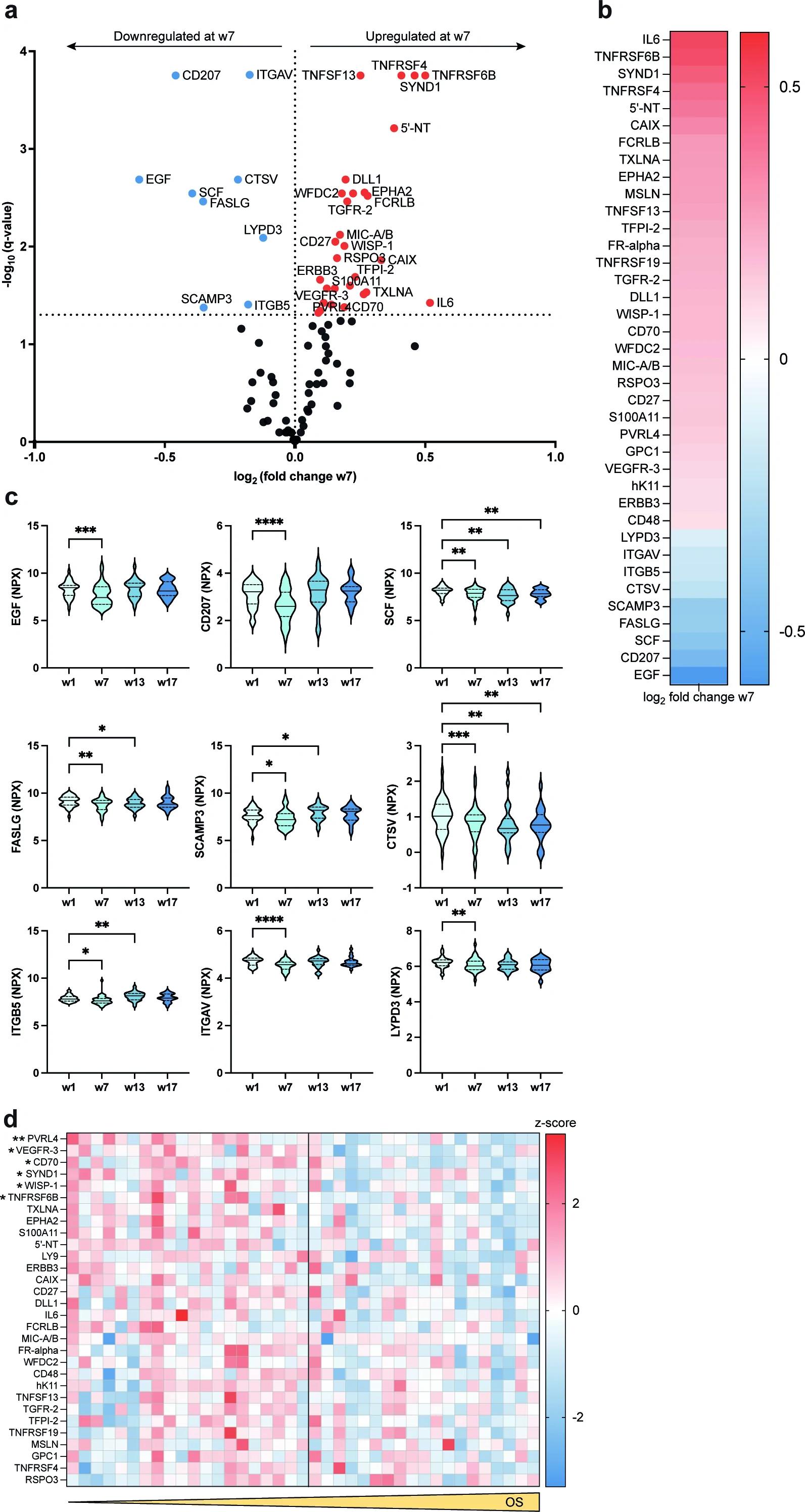
Oncology plasma biomarkers. Plasma samples from patients in the LOKON002 clinical trial were analyzed with Olink Target 96 Oncology II panel. The volcano plot shows the log_2_ fold change of plasma proteins at w7 compared with baseline (w1) (x-axis) (**a**). Statistically significant differences were determined with multiple paired t-tests followed by FDR method of Benjamini and Hochberg (FDR=5%) and negative logarithm of adjusted p values are calculated and displayed as q value in the y-axis. The horizontal and vertical dotted line represents the cut-off for significant q values and for upregulation versus downregulation, respectively. The heatmap shows the significantly changed proteins at w7 (**b**). In “a” and “b”, red and blue color represent an upregulation and downregulation, respectively. Violin plots with lines representing median and lower and upper quartiles show the expression over time of the downregulated genes identified at w7 (**c**). Statistical differences in “c” were determined Mixed-effects model followed by Dunnett’s multiple comparison test (*p≤0.05, **p≤0.01, ***p≤0.001, ****p≤0.0001). Samples analyzed at baseline (w 1; n=39; pancreatic n=27; colorectal n=5; ovarian n=4; biliary n=3), w7 (n=39, same as w 1), w13 (n=29; pancreatic n=21; colorectal n=2; ovarian n=4, biliary n=2), and at w17 (n=21; pancreatic n=15; colorectal n=2; ovarian n=3, biliary n=1). Drop out of samples over time were due to progression. Patients were divided in two groups based on their median OS and overall upregulated proteins were displayed on an individual patient level in a heatmap (**d**). Differential expression was analyzed with multiple unpaired t-tests followed by Benjamini and Hochberg (FDR=5%). Significant differences are highlighted in the heatmap with asterisks. FDR, false discovery rate; OS, overall survival; w, week.

To further explore proteins of interest over time, data were divided by biological role and tumor response. These data confirmed that proteins associated with immune activation and T cells and NK cells were more often increased in patients during PR or SD, while proteins associated with immunosuppression, angiogenesis, or other factors promoting tumor growth were more often increased in patients during PD (online supplemental figures 2–6).

### Distinct TME gene expression profile in responding patients with pancreatic cancer

The vast majority of patients in this study had pancreatic cancer, of which there were 14 matched pre and post (week 13) biopsy samples available. Hence, an in-depth TME-focused NanoString analysis was performed in patients with pancreatic cancer to characterize immunological effects in patients with longer survival and/or response to treatment. Figure 5a displays differential fold change gene expression in the TME at week 13 between patients surviving shorter or longer than median OS (10.8 months) in this particular study. The significant differentially changed genes are shown on an individual patient level in a heatmap in figure 5b (online supplemental table 5). Tumors from patients with shorter survival showed a higher fold change of genes encoding for components of the complement system (C3, C1R, C4B, C1S), interferon regulating genes (fasciculation and elongation protein zeta-1 [FEZ1]), and proteins involved in cell signaling, adhesion, vascular remodeling and migration (amyloid precursor protein (APP), integrin β1, and CD99). Additionally, genes implicated in antiviral responses (interleukin enhancer binding factor 3 [ILF3]) were higher in short-term survivors. Genes that were significantly higher in patients with longer survival were further explored in GO enrichment analysis and displayed in a functional protein interaction network (STRING database) in figure 5c. Tumors from longer-surviving patients displayed enriched adaptive T-cell responses (IFN-γ, IL-21, LCK, FPR2, TBX21 (Tbet), RUNX3, and EOMES) and innate immune pathways (CAMP (encoding for cathelicidin antimicrobial peptide), and KIR2DL2) together with myeloid cell activation (CLERC4A, CLERC6A, IL-18, and TNF), all supporting a robust Th1-associated immune response in the TME. For example, further, genes related to autophagy (ATG5, RIPK2, TRIM39), which is implicated in the adenoviral oncolysis and necroptosis process, were higher in patients with longer OS. Of interest, CD40, a target receptor of LOAd703 therapy, emerged as a center node of the protein interaction network, supporting LOAd703’s primary mechanism of action to reprogram myeloid cells to enhance antigen presentation to T cells and promote Th1 effector cells via CD40-mediated DC activation.

**Figure 5:**
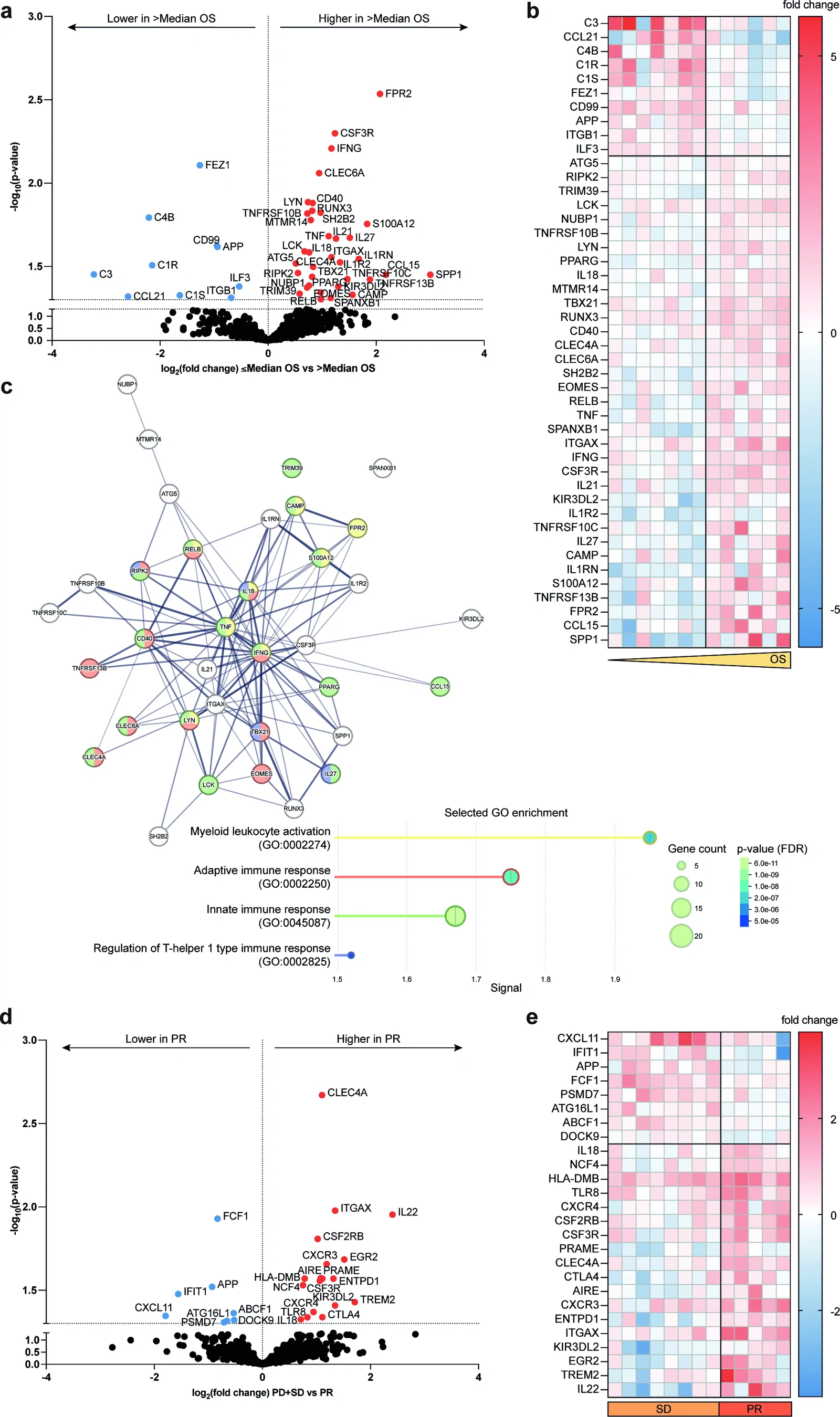
Differential gene expression in tumors of patients with pancreatic cancer. Tumor biopsies were analyzed with nCounter PanCancer Immune Profiling Panel (NanoString Technologies) at baseline and w 13 (n=14), the time point for the after treatment initiation biopsy. The data were normalized with RUV-4 method in RStudio and approximated individual values were calculated by removing estimated unwanted factors calculated during the RUV-4 normalization from the observed log_2_ gene counts. Patients with pancreatic cancer (n=14) were divided based on their median OS and differential gene expression between both groups was determined with multiple unpaired t-tests and the results are displayed in a volcano plot, where the log_2_ difference in expression is displayed on the x-axis and the q value on the y-axis (**a**). Significantly differentially expressed genes between shorter and longer surviving patients are shown above the dotted line. The heatmap displays the approximated individual log_2_ fold change of the significantly changed genes at w13 compared with baseline (**b**). Patients are sorted based on their OS in the heatmap. The interaction of the encoded proteins of genes that were upregulated in patients surviving above median OS is shown in a functional association network (STRING) (**c**). The nodes are colored based on selected enriched GO pathways, which are displayed according to their signal (weighted harmonic mean between the ratio of observed vs expected genes in the respective pathway and the FDR). FDR adjusted p values are shown as a color gradient and the observed gene counts are estimated by symbol size. Patients with pancreatic cancer were divided based on their best response (SD, n=8 vs PR, n=5) and differential gene expression between SD versus PR was determined with multiple unpaired t-tests and displayed in a volcano plot (**d**). Significantly differentially expressed genes are shown above the dotted line. The heatmap displays the approximated individual log_2_ fold change of the significantly changed genes at w13 compared with baseline (**e**). Patients are sorted based on their best response in the heatmap. FDR, false discovery rate; GO, Gene Ontology; OS, overall survival; PD, progressive disease; PR, partial response; SD, stable disease; w, week.

Next, gene expression fold change differences between patients experiencing a tumor response according to RECIST V.1.1 were determined (PR vs SD+PD) (figure 5d,e, online supplemental table 6). Responding patients showed lower expression of genes encoding proteins involved in antiviral response (IFT1), degradation and ubiquitination (PSMD7, ABCF1), autophagy (ATG16L1), cell proliferation, adhesion, and migration (FCF1, APP), but also CXCL11 attracting T cells to the site. Most significant upregulation was observed for CLEC4A, which is a receptor essential for DC cross-presentation. Of interest, also HLA-DMB, which is part of the major histocompatibility complex (MHC) II antigen presentation machinery, and CXCR3, which is expressed on Th1 cells and binds to CXCL9, CXCL10, CXCL11, were increased to a greater extent in responding patients.

### Decrease in unfavorable plasma proteins associates with increased T-cell and B-cell response biomarkers in the TME of patients with pancreatic cancer

To identify potential response biomarkers in plasma, patients with pancreatic cancer were divided into PD, SD or PR as best response according to RECIST V.1.1. and significantly changed plasma proteins at week 7 compared with baseline were determined for each response group (figure 6a, online supplemental table 7). Interestingly, there was a striking pattern in terms of upregulation versus downregulation of immune-associated proteins depending on response. Responding patients predominantly exhibited downregulated plasma proteins, markedly distinct from progressing patients who displayed widespread protein upregulation. Notably, IFN-γ was the only protein upregulated in responders and patients experiencing SD that was not elevated in progressing patients. To pinpoint differences between progressing and responding patients, expression levels and fold change of all investigated proteins at week 7 were compared between PD and PR and significant differences were displayed on an individual patient level in heatmaps (figure 6b,c). Overall, all significantly changed proteins were lower in responders than in progressing patients and eight proteins (ANGPT1, PDGFB, CAIX, MMP7, LAP transforming growth factor (TGF)β1, IL-7, MCP-3, TNFSF14) showed both a lower absolute level and a negative fold change at week 7, meaning that these decreased to a higher degree in responding patients. Further, differential expression at week 7 between patients surviving longer or shorter based on median OS threshold was determined (figure 6d). Patients with longer survival had significantly lower plasma levels of ANGPT2, HGF, MMP7, CSF-1, LAP TGFβ1, TNFSF14, CCL20, CD70, and CD83. In general, the responding patients had reduced levels of proteins related to poor prognosis such as tumor growth, immunosuppression, and angiogenesis. Therefore, they were explored as potential biomarkers for OS based on the median expression level of the respective protein at week 7. All statistically significant survival curves are shown in figure 6e. Overall, lower levels of the identified plasma proteins were associated with longer survival. To explore the connection of plasma proteins and TME gene expression, fold changes of all analyzed plasma proteins were correlated with the respective TME gene expression fold change at week 13 (figure 6f). Statistically significant correlations for each plasma protein shown in figure 6e with genes expressed in the TME are displayed in figure 6g. Patients with a plasma protein profile that is associated with longer survival correlated with an intratumoral increase in genes related to T-cell responses (CD27, CD5, CD40L, GZMH, CD8A, CD70, PD-1, CCL19), and to a decrease in genes implicated in myeloid cell recruitment and activity (CXCL1, CXCL5, CCL3, CCL20, CCL7, IL-6) and the ICOS/ICOSL pathway (ICOSLG).

**Figure 6:**
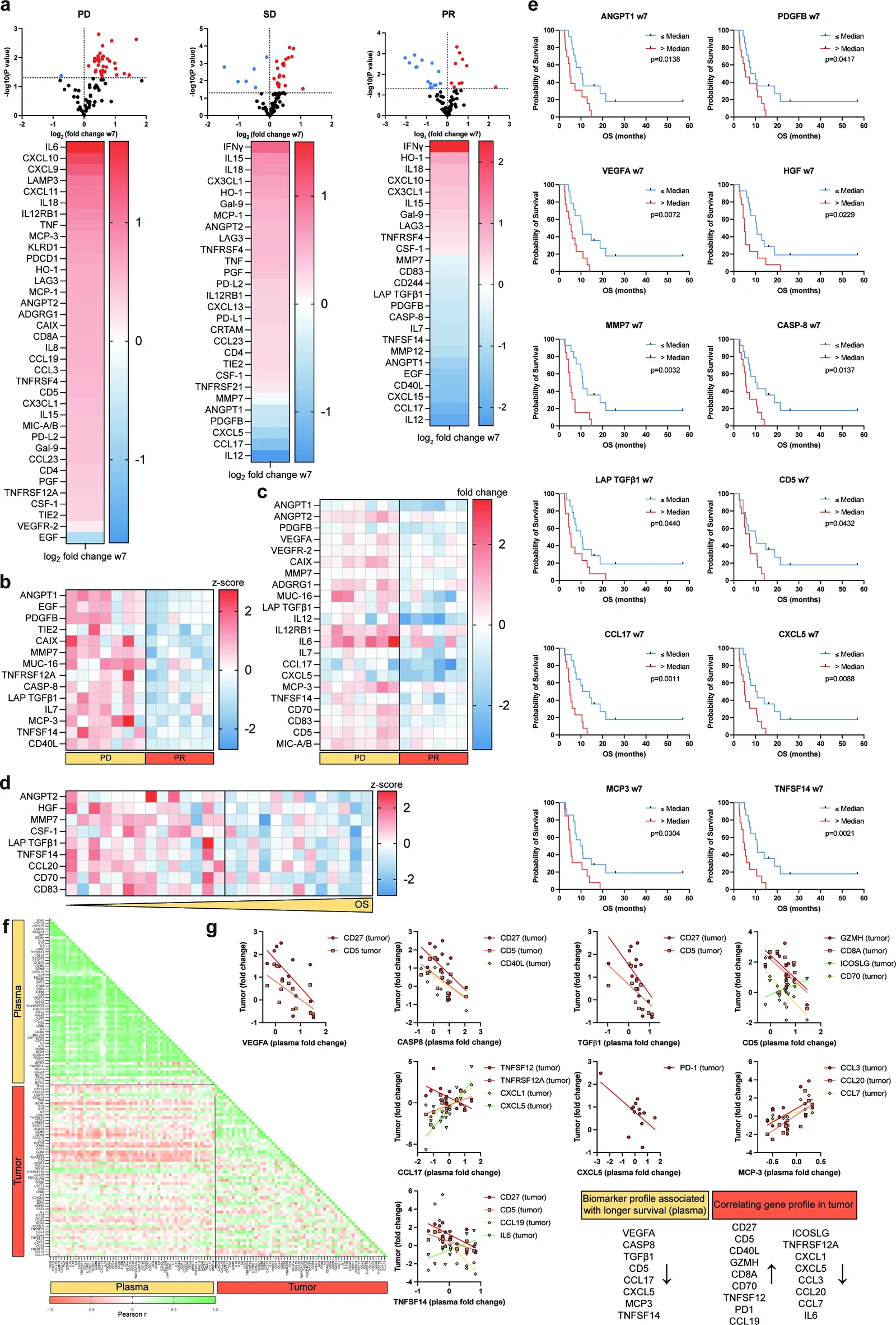
Immunological plasma biomarkers in patients with pancreatic cancer. Plasma samples were analyzed with Olink Target 96 Immuno-oncology panel. Efficacy evaluable (those with at least one second imaging post baseline) patients with pancreatic cancer (n=25) were divided based on their best response: (PD, n=7), (SD, n=12) and (PR, n=6). Volcano plots show the log_2_ fold change of plasma proteins at w 7 compared with baseline of each response group (**a**). Statistically significant differences were determined with multiple paired t-tests and upregulated proteins are shown in red, while downregulated proteins are shown in blue in the volcano plots and heatmaps. In “b” and “c”, differential protein expression between patients with PD and PR as best response were evaluated with multiple unpaired t-tests and significant different proteins are displayed on an individual patient level in heatmaps as z-score (**b**) or fold change (**c**). The actual expression level at w7 (NPX) was used for calculations (**b**), while differences in the fold change from baseline to w7 were shown in “c”. Patients were divided in two groups based on their median OS (≤median OS: n=14, >median OS: n=13) and differential expression at w7 analyzed with multiple unpaired t-tests. Significant differences in expression between both OS groups are displayed on an individual patient level in a heatmap (**d**). Patients were divided in two groups based on the median expression at w7 of the respective analyte of interest and differences in the Kaplan-Meier curves were calculated with log-rank (Mantel-Cox) test (**e**). The correlation matrix shows the correlation (Pearson) of the fold change of plasma immuno-oncology markers with the fold change of the respective genes in the tumor at w13 (**f**). Significant correlation of the identified plasma biomarkers with genes in the tumor are displayed with regression lines (**g**). NPX, normalized protein expression; OS, overall survival; PD, progressive disease; PR, partial response; SD, stable disease; w, week.

### Distinct profiles of plasma oncology proteins in progressing and responding patients with pancreatic cancer

Analysis using the oncology panel, comprising proteins predominantly associated in tumor pathogenesis, revealed a similar pattern and profound differences between progressing and responding patients at week 7. Strikingly, patients with PD exhibited widespread upregulation of oncogenic proteins, while patients with PR demonstrated an almost exclusive pattern of protein downregulation. The immune receptor OX40 (TNFRSF4), was the only protein increased in responding patients using this panel, but it was likewise increased in SD and PD patients (figure 7a). Differential expression analysis between PD and PR patients further confirmed these findings. Responding patients consistently showed lower expression of the respective protein compared with progressive patients (figure 7b). The same pattern persisted when evaluating the fold change from baseline (figure 7c). Of note, EGF was downregulated in both patient groups, but to a greater extent in responders. When stratifying patients with pancreatic cancer by their median OS in the study, a similar pattern emerged regarding the presence of the oncology proteins. Only SCF levels were overall higher in longer surviving patients, while all other significantly differential expressed proteins were lower (figure 7d). Figure 7e shows all investigated proteins that separated survival curves based on their median expression at week 7. These included cell adhesion protein CEACAM5, signaling proteins (WIF-1, WISP-1), growth factors and growth factor receptor (amphiregulin (AREG), VEGF-A, IGF1R), as well as immune receptors (FCRLB, CD70). Six additional proteins (PVRL4, ADAM8, TGFα, MK, IFNgR1, CD48) showed significant differences at week 7, but also at baseline (week 1, data not shown), which indicates that these proteins may have general prognostic/predictive value in pancreatic cancer. Finally, an univariant Cox regression analysis was performed for the in figures 6e and 7e identified oncology (figure 7f) and immuno-oncology (figure 7g) panels to further evaluate their impact on survival based on their expression at week 7. For the selected oncology proteins, only the EGFR ligand AREG was associated with an increased risk of death, whereas most of the selected immuno-oncology proteins were statistically significant with HGF, MMP7, and CD5 having the most impact on survival.

**Figure 7:**
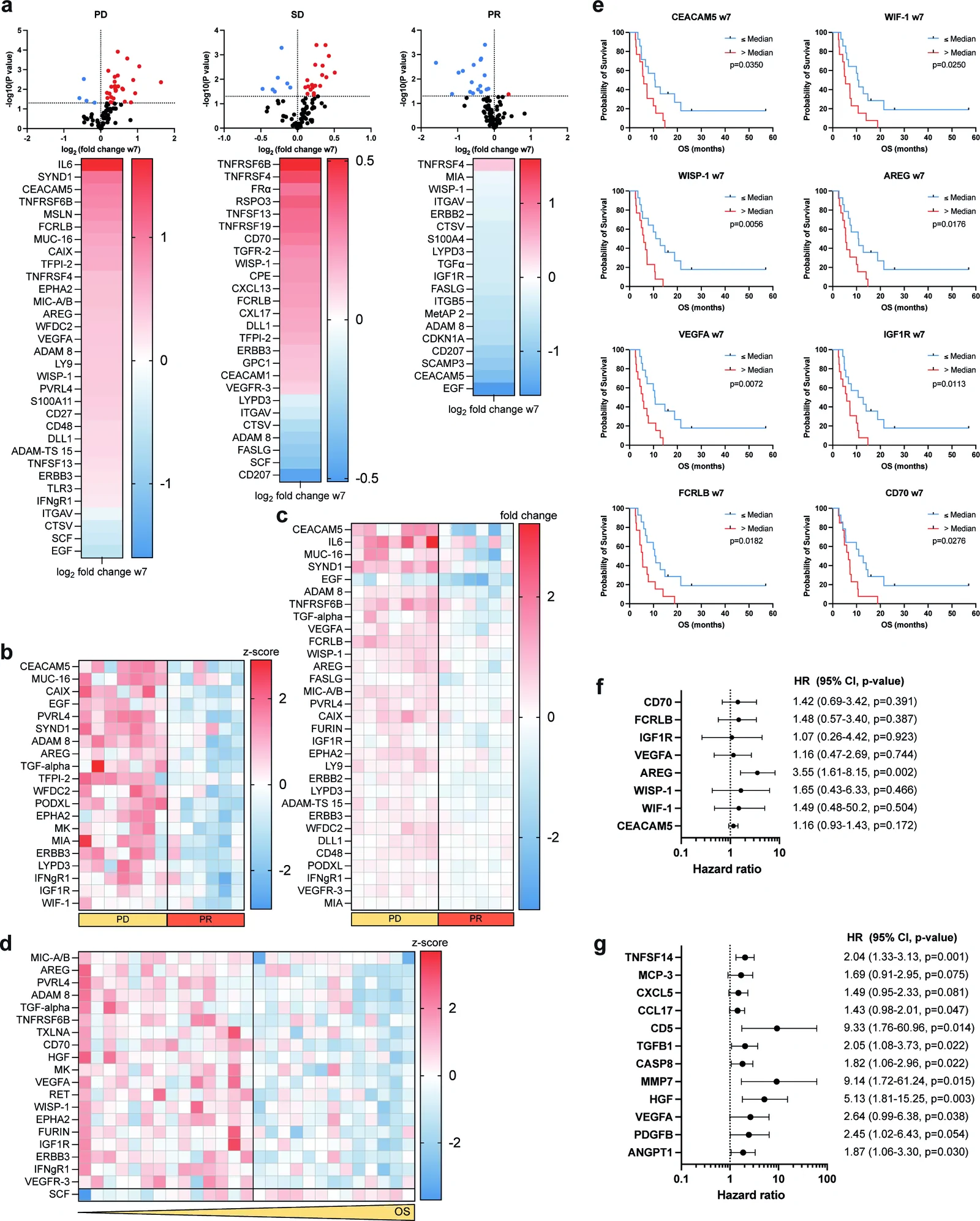
Oncology plasma biomarkers in patients with pancreatic cancer. Plasma samples were analyzed with Olink Target 96 Oncology II panel. Efficacy evaluable (those with at least one second imaging post baseline) pancreatic cancer patients (n=25) were divided based on their best response: (PD, n=7), (SD, n=12) and (PR, n=6). Volcano plots show the log_2_ fold change of plasma proteins at w 7 compared with baseline of each response group (**a**). Statistically significant differences were determined with multiple paired t-tests and upregulated proteins are shown in red, while downregulated proteins are shown in blue in the volcano plots and heatmaps. Differential protein expression between patients with PD and PR as best response were evaluated with multiple unpaired t-tests and significant different proteins are displayed on an individual patient level in heatmaps as z-score (**b**) or fold change (**c**). In “b”, the actual expression level at w7 (NPX) was used for calculations, while in “c” differences in the fold change from baseline to w7 were investigated. Patients were divided into two groups based on their median OS (≤median OS: n=14, >median OS: n=13) and differential expression at w7 was analyzed with multiple unpaired t-tests (**d**). Significant differences in expression between both OS groups are displayed on an individual patient level in a heatmap (**d**). Patients were divided into two groups based on the median expression at w7 of the respective analyte of interest and differences in the Kaplan-Meier curves were calculated with log-rank (Mantel-Cox) test. Forest plots in “f” and “g” display the HRs with 95% CI of the investigated oncology (**f**) and immuno-oncology (**g**) biomarkers calculated with univariate Cox regression analysis. NPX, normalized protein expression; OS, overall survival; PD, progressive disease; PR, partial response; SD, stable disease; w, week.

## Discussion/conclusion

Tumors with few infiltrating T cells and a high myeloid cell content, such as pancreatic cancer, are in dire need of innovative therapeutic strategies capable of transforming the immunosuppressive TME into an immunologically active milieu, in which T-cell and NK cell responses can thrive.^23^ Our investigation strongly supports that intratumoral viral-based TME gene engineering with LOAd703 in combination with chemotherapy modulates the TME to express immunostimulatory molecules and shifts the balance from an inherently hostile environment towards a pro-inflammatory state, which could allow for simultaneous treatment with immune checkpoint inhibitors.

Our heterogeneous patient cohort included individuals with various immune-resistant malignancies (pancreatic, ovarian, colorectal or biliary cancer) with pancreatic cancer representing the majority. Further, the prior treatments greatly varied in number, which also allowed for LOAd703 to be combined with gemcitabine only in the majority of patients or in combination with nab-paclitaxel (pancreatic cancer) or cisplatin (biliary cancer). Despite the heterogeneity, LOAd703-based combination therapy consistently induced significant inflammatory responses, characterized by robust antigen presentation and adaptive immune responses with T-cell and B-cell signatures. Notably, transcriptomic analysis of pancreatic tumors has previously revealed a better outcome for tumors enriched in adaptive immune cells compared with innate or myeloid cells.^24 25^ In these tumors, the majority of antigen presenting cells had B-cell origin, which emphasizes that B cells greatly contribute to induce T cell-mediated antitumor immunity.^24^ Among the elevated inflammatory mediators, the chemokine CCL5 exhibited the greatest upregulation. CCL5 has been extensively associated with the infiltration of CD8+T cells, B cells, DCs, and macrophages, as well as with improved responses to immunotherapy across multiple cancer types.^26^ Further, LOAd703 and chemotherapy combination treatment strongly induced gene signatures in the TME that have previously been validated as predictive biomarkers to immune checkpoint inhibitor therapy.^21 22^ The concurrent upregulation of multiple checkpoint molecules in response to LOAd703/chemotherapy-induced inflammation, provides a strong rationale for exploring therapeutic combinations of LOAd703 and chemotherapy with checkpoint inhibitors. Indeed, the triplet combination of LOAd703, chemotherapy, and anti-PD-L1 inhibition is currently being evaluated in pancreatic cancer in the second arm of the LOKON001 trial (NCT02705196) and a trial combining LOAd703 plus anti-PD-L1 therapy for PD-1 refractory malignant melanoma was recently completed (NCT04123470).^27^ In these PD-1 refractory melanoma patients, there was similarly an increase of proteins (blood) and mRNA (TME) related to antigen presentation and T-cell response, but also an upregulated tertiary lymphoid structure signature after treatment initiation.^28^

A frequently discussed concern for intratumoral therapies is their perceived lack of systemic efficacy. While this study primarily analyzed paired injected tumor biopsies, our data also demonstrate relevant systemic immunological changes extending beyond the local tumor environment. We observed substantial systemic immune modulation, seen both in plasma and in the composition of immune cells in patients’ blood after the LOAd703 and chemotherapy combination treatment. Two major soluble factors associated with immunotherapy response, IFN-γ and CXCL10,^29 30^ were increased in plasma samples after treatment. IL-15, which is structurally similar to IL-2 and regulates NK and CD8+T cell proliferation and survival, was also upregulated in patients’ plasma. IL-15 has been suggested to have a critical role in the generation and maintenance of CD8+memory T cells.^31 32^ In line with this, we detected an increase of circulating effector memory CD8+T cells in the patients, and their levels were higher in responding patients. This effector memory T-cell signature is associated with clinical response to immune checkpoint inhibition,^33^ suggesting that LOAd703 together with chemotherapy potently shapes systemic immunity toward a phenotype favorable for antitumor activity. It should be acknowledged that LOAd703 was administered combined with gemcitabine-based chemotherapy, and therefore one cannot rule out that some of the systemic effects were related to the chemotherapy alone. For example, we have previously demonstrated the immune-modulating effects of gemcitabine, such as the reduction of peripheral TGF-β1, myeloid-derived suppressor cells (MDSCs), Tregs, and NK cells.^34^ Likewise, we noted a reduction of NK cells and Tregs, accompanied by decreased plasma levels of Treg-recruiting chemokine CCL17. The immunosuppressive chemokine CXCL5, which is connected to poor response to immunotherapy,^35^ was also reduced in plasma. CD40L can be shed from activated cells as well as platelets and elevated CD40L in the blood of patients with cancer is suggested to have immunosuppressive effects.^36 37^ Interestingly, plasma levels of CD40L were decreased post LOAd703 treatment. This observation highlights an important advantage of our engineered trimerized, membrane-bound form of CD40L expressed by LOAd703 as it is protected from shedding compared with wild type CD40L.^38^ Detection of the transgenes in the TME, however, is not likely 2 weeks post LOAd703 gene therapy, as the adenovirus infected cells will be killed by the immune system or by oncolysis, which warrants a tumor biopsy in close proximity to treatment in upcoming studies.

In-depth analysis of patients with pancreatic cancer provided notable insights into factors associated with a better than study median survival. The TME of patients with longer survival than the study median was enriched for genes of an adaptive immune response and displayed increased expression of CD40, which is the target receptor for LOAd703-expressed TMZ-CD40L and its central node role in network analysis. This suggests that LOAd703 was likely the primary driver of immune activation within the TME, rather than chemotherapy alone. In support of this conclusion, Rupp *et al* compared the TME of primary resected pancreatic tumors with the TME of tumors resected after neoadjuvant chemotherapy and observed that chemotherapy instead resulted in a downregulation of adaptive immune pathways.^39^ Nevertheless, the role of chemotherapy when used together with LOAd703 may be different. An adaptive antitumor immune response is driven by effector CD8+T cells and these are regulated by the transcription factor Eomesodermin (EOMES).^40 41^ Interestingly, tumors from patients with prolonged survival exhibited higher EOMES levels along with elevated levels of key effector molecules IFN-γ and tumor necrosis factor (TNF). These findings align with previously reported results linking intratumoral CD40 expression with Th1 immune responses and improved survival across cancers.^42^ As LOAd703 is based on an adenoviral vector that selectively replicates in tumor cells, direct tumor cell lysis is part of LOAd703’s mechanism of action. Oncolytic adenoviruses induce autophagy in tumor cells, which is thought to facilitate viral replication.^43^ In our study, patients with longer survival than study median had a higher increase of the core autophagy regulator ATG5 that is otherwise related to tumor progression, indicating increased viral replication in these patients. ATG5 has also been implicated in the priming of tumor-specific CD4+T cells.^44^ Collectively, these data support LOAd703’s dual mechanism of action to promote efficient antitumor responses through direct tumor debulking via oncolysis and robust immune priming through transgene-driven immune activation and facilitation of antigen presentation, but it still remains unclear if the same effect would be seen without the concurrent chemotherapy.

When comparing the factors identified with longer than study median survival and response, five factors were overlapping and may be associated with a clear clinical benefit within our study. Three of these genes encode for DC markers: ITGAX (CD11c), CLEC4A, and IL-18. The other overlapping genes are killer cell immunoglobulin-like receptor 3DL2 (KIR3DL2) and CSF3R (granulocyte colony-stimulating factor receptor; GCSFR). ITGAX has been associated with prolonged OS and response to immunotherapy in different cancers.^45–47^ DCIR mediates cross-presentation and activation of antigen-specific CD8+T cell responses, especially when combined with toll-like receptor (TLR)7/8 activation and triggering of the CD40 pathway.^48^ Interestingly, TLR8 and MHC class II molecule HLA-DMB were also upregulated in responding tumors, further supporting the induction of a DC-driven and antigen presentation-rich signature in response to LOAd703 combination therapy. High CLEC4A expression has also been correlated with increased immune cell infiltration and improved clinical outcome in colorectal and lung cancer.^49 50^ KIR3DL2 binds to MHC class I molecules and IGSF8.^51 52^ IGSF8 is overexpressed in many tumors and is suggested to inhibit antitumor NK-cell responses.^52^ However, in line with our study, low expression of KIR3DL2 has been associated with reduced OS in patients with liver cancer.^53^ CSF3R is expressed on myeloid cells, including neutrophils, but is also overexpressed in many cancers and generally linked with tumor progression.^54^ High CSF3R expression has been associated with shorter survival in glioma patients.^55^ In our study, upregulation of CSF3R expression in responding patients may reflect an activated neutrophil phenotype. This is supported by the concurrent elevation of neutrophil cytosol factor 4. Neutrophils are generally considered to have a protumoral role, but in the context of CD40 activating immunotherapy, antitumor neutrophilic responses have been observed, and this response was dependent on the presence of DCs, IL-12 and IFN-γ.^56^ E-selectin is also upregulated by inflammatory cytokines on endothelial cells and facilitates lymphocyte rolling, leading to adhesion and infiltration of T cells in tumors.^57 58^

Proteomics analysis of plasma from the patients with pancreatic cancer allowed us to identify potential biomarkers associated with poor outcomes in this cohort. These included growth factors and factors involved in angiogenesis and tissue remodeling, cell adhesion, signaling, and immunosuppression. Many of these factors, such as VEGF, TGFβ, HGF, PDGFB, and AREG, are involved in the crosstalk between pancreatic stellate cells/fibroblasts and tumor cells and play a major role in the immunosuppressive TME.^59 60^ In vitro, we have previously shown a downregulation of TGF-β, hepatocyte growth factor (HGF) and AREG in stellate cells in response to LOAd703 infection^7^ and in the total patient cohort, several of these tumor-promoting factors were reduced after treatment initiation.

In the TME, patients with shorter survival had higher levels of complement system components, which have an immunosuppressive and tumor-promoting role in the TME through their interaction with tumor-associated macrophages and neutrophils.^61^ Also, the antiviral factor ILF3 was higher in the TME of patients with shorter survival. In line with this, non-responding tumors had higher expression of antiviral factor IFT1. Thus, LOAd703 treatment effect may be hampered by a stronger antiviral immunity in some patients. Of note, ILF3 has also been shown to have a role in suppressing DC maturation^62^ and therefore may hinder DC activation in these patients. The immunosuppression in the pancreatic TME is driven by tumor-promoting macrophages and MDSCs. In progressing patients, factors associated with M2 polarization of macrophages and expansion of MDSCs were increased, such as LAP-TGFβ1, CCL20, CSF-1, and HGF.

Due to the sample size and advanced statistics for the number of analytes per sample, it was not possible to further divide the study population considering all parameters of interest such as LOAd703 dose level, first line versus second line, all different indications separated and so forth. However, the presented analysis will be guiding the forthcoming analyses performed on patients treated with LOAd703 in more homogeneous groups and treated randomized to control arms.

Given the evolving landscape of pancreatic cancer due to the approval of the RAS(ON) multi-selective inhibitor, daraxonrasib, as a monotherapy for second line patients,^63^ a LOAd703 combination with daraxonrasib is highly relevant. A study by Orlen *et al*, recently demonstrated that KRAS inhibitor treatment decreased myeloid cells while increasing T cells in immunocompetent pancreatic cancer models. Further, the treatment effect was potentiated by the combination with a CD40 antibody immunotherapy.^64^ The role of KRAS inhibitors on the immune system was elegantly reviewed recently.^65^

To summarize, TME gene engineering therapy with LOAd703 in combination with chemotherapy facilitated an immune responsive profile in commonly immunotherapy resistant tumors by inducing an adaptive immune response. This response was characterized by Th1 polarization and enhanced antigen presentation signatures, along with gene expression profiles associated with favorable responses to immune checkpoint inhibitors. Notably, these intratumoral effects were accompanied by systemic immune activation detectable in peripheral blood, underscoring the broad immunologic impact of LOAd703 therapy when combined with chemotherapy. Patients with pancreatic cancer with longer than median survival in this study displayed gene signatures consistent with adaptive immune responses, particularly markers of cross-presenting DCs, as well as autophagy-related signatures linked to oncolysis. In contrast, patients with shorter survival exhibited gene signatures indicative of tumor-supportive myeloid cells and fibroblasts, antiviral resistance mechanisms, and complement activation. Even if this study is performed on a small population of patients and underpowered for proper biomarker identification, collectively, these findings support the emerging paradigm that LOAd703 and similar TME gene engineering treatments in combination with chemotherapy, may unlock the immunotherapeutic potential of traditionally immunotherapy unresponsive tumors, such as pancreatic cancer.

## Supplementary material

10.1136/jitc-2026-016908 online supplemental file 1

10.1136/jitc-2026-016908 online supplemental file 2

## Data Availability

Data are available upon reasonable request. Data may be obtained from a third party and are not publicly available.

## References

[1] Morad G, Helmink BA, Sharma P (2021). Hallmarks of response, resistance, and toxicity to immune checkpoint blockade. Cell.

[2] Bockorny B, Grossman JE, Hidalgo M (2022). Facts and Hopes in Immunotherapy of Pancreatic Cancer. Clin Cancer Res.

[3] Xiao Z, Todd L, Huang L (2023). Desmoplastic stroma restricts T cell extravasation and mediates immune exclusion and immunosuppression in solid tumors. Nat Commun.

[4] Fucikova J, Coosemans A, Orsulic S (2021). Immunological configuration of ovarian carcinoma: features and impact on disease outcome. J Immunother Cancer.

[5] Cammarota A, Balsano R, Pressiani T (2025). The Immune-Genomics of Cholangiocarcinoma: A Biological Footprint to Develop Novel Immunotherapies. Cancers (Basel).

[6] Gatenbee CD, Baker A-M, Schenck RO (2022). Immunosuppressive niche engineering at the onset of human colorectal cancer. Nat Commun.

[7] Eriksson E, Milenova I, Wenthe J (2017). Shaping the Tumor Stroma and Sparking Immune Activation by CD40 and 4-1BB Signaling Induced by an Armed Oncolytic Virus. Clin Cancer Res.

[8] Quezada SA, Jarvinen LZ, Lind EF (2004). CD40/CD154 interactions at the interface of tolerance and immunity. Annu Rev Immunol.

[9] Kotowicz K, Dixon GLJ, Klein NJ (2000). Biological function of CD40 on human endothelial cells: costimulation with CD40 ligand and interleukin-4 selectively induces expression of vascular cell adhesion molecule-1 and P-selectin resulting in preferential adhesion of lymphocytes. Immunology.

[10] Eliopoulos AG, Davies C, Knox PG (2000). CD40 induces apoptosis in carcinoma cells through activation of cytotoxic ligands of the tumor necrosis factor superfamily. Mol Cell Biol.

[11] Chester C, Sanmamed MF, Wang J (2018). Immunotherapy targeting 4-1BB: mechanistic rationale, clinical results, and future strategies. Blood.

[12] Labani-Motlagh A, Naseri S, Wenthe J (2021). Systemic immunity upon local oncolytic virotherapy armed with immunostimulatory genes may be supported by tumor-derived exosomes. Mol Ther Oncolytics.

[13] Wenthe J, Naseri S, Hellström A-C (2022). Immune priming using DC- and T cell-targeting gene therapy sensitizes both treated and distant B16 tumors to checkpoint inhibition. Mol Ther Oncolytics.

[14] Wenthe J, Naseri S, Hellström A-C (2020). Immunostimulatory oncolytic virotherapy for multiple myeloma targeting 4-1BB and/or CD40. Cancer Gene Ther.

[15] Wenthe J, Naseri S, Labani-Motlagh A (2021). Boosting CAR T-cell responses in lymphoma by simultaneous targeting of CD40/4-1BB using oncolytic viral gene therapy. Cancer Immunol Immunother.

[16] Naseri S, Cordova MM, Wenthe J (2024). CD40 stimulation via CD40 ligand enhances adenovirus‐mediated tumour immunogenicity including ‘find‐me’, ‘eat‐me’, and ‘kill‐me’ signalling. *J Cellular Molecular Medi*.

[17] Musher BL, Rowinsky EK, Smaglo BG (2024). LOAd703, an oncolytic virus-based immunostimulatory gene therapy, combined with chemotherapy for unresectable or metastatic pancreatic cancer (LOKON001): results from arm 1 of a non-randomised, single-centre, phase 1/2 study. Lancet Oncol.

[18] Hamid O, Rydén V, Mehmi I (2024). 988O A phase I/II trial (LOKON003) evaluating tumor microenvironment (TME) gene engineering using a viral vector combined with atezolizumab in advanced malignant melanoma. Ann Oncol.

[19] Hahn A, Irenaeus S, Sandin LC (2026). Tumor Microenvironment Gene Engineering with LOAd703 in Patients with Solid Malignancies: LOKON002 Phase I/IIb Clinical Trial. Clin Cancer Res.

[20] Szklarczyk D, Kirsch R, Koutrouli M (2023). The STRING database in 2023: protein-protein association networks and functional enrichment analyses for any sequenced genome of interest. Nucleic Acids Res.

[21] Ayers M, Lunceford J, Nebozhyn M (2017). IFN-γ-related mRNA profile predicts clinical response to PD-1 blockade. J Clin Invest.

[22] Fehrenbacher L, Spira A, Ballinger M (2016). Atezolizumab versus docetaxel for patients with previously treated non-small-cell lung cancer (POPLAR): a multicentre, open-label, phase 2 randomised controlled trial. Lancet.

[23] Farhangnia P, Khorramdelazad H, Nickho H (2024). Current and future immunotherapeutic approaches in pancreatic cancer treatment. *J Hematol Oncol*.

[24] Sivakumar S, Jainarayanan A, Arbe-Barnes E (2025). Distinct immune cell infiltration patterns in pancreatic ductal adenocarcinoma (PDAC) exhibit divergent immune cell selection and immunosuppressive mechanisms. Nat Commun.

[25] de Santiago I, Yau C, Heij L (2019). Immunophenotypes of pancreatic ductal adenocarcinoma: Meta-analysis of transcriptional subtypes. Int J Cancer.

[26] Shan J, Xu Y, Lun Y (2024). Comprehensive analysis of the potential biological significance of CCL5 in pan-cancer prognosis and immunotherapy. Sci Rep.

[27] Hamid O, Ekström-Rydén V, Mehmi I (2026). LOAd703-induced tumor microenvironment gene engineering in combination with atezolizumab in metastatic malignant melanoma: a phase I/II trial. Nat Commun.

[28] Grauers Wiktorin H, Ekström-Rydén V, Ek I (2026). Reprogramming myeloid cells and restoring T cell fitness in checkpoint inhibitor resistant melanoma patients. Biomark Res.

[29] Reschke R, Yu J, Flood BA (2021). Immune cell and tumor cell-derived CXCL10 is indicative of immunotherapy response in metastatic melanoma. J Immunother Cancer.

[30] Karachaliou N, Gonzalez-Cao M, Crespo G (2018). Interferon gamma, an important marker of response to immune checkpoint blockade in non-small cell lung cancer and melanoma patients. Ther Adv Med Oncol.

[31] Schluns KS, Williams K, Ma A (2002). Cutting edge: requirement for IL-15 in the generation of primary and memory antigen-specific CD8 T cells. J Immunol.

[32] Becker TC, Wherry EJ, Boone D (2002). Interleukin 15 is required for proliferative renewal of virus-specific memory CD8 T cells. J Exp Med.

[33] Fairfax BP, Taylor CA, Watson RA (2020). Peripheral CD8^+^ T cell characteristics associated with durable responses to immune checkpoint blockade in patients with metastatic melanoma. Nat Med.

[34] Eriksson E, Wenthe J, Irenaeus S (2016). Gemcitabine reduces MDSCs, tregs and TGFβ-1 while restoring the teff/treg ratio in patients with pancreatic cancer. J Transl Med.

[35] Deng J, Ma X, Ni Y (2022). Identification of CXCL5 expression as a predictive biomarker associated with response and prognosis of immunotherapy in patients with non-small cell lung cancer. Cancer Med.

[36] Pazoki A, Dadfar S, Shadab A (2024). Soluble CD40 Ligand as a Promising Biomarker in Cancer Diagnosis. Cells.

[37] Chung HW, Lim J-B (2014). Clinical significance of elevated serum soluble CD40 ligand levels as a diagnostic and prognostic tumor marker for pancreatic ductal adenocarcinoma. J Transl Med.

[38] Eriksson E, Moreno R, Milenova I (2017). Activation of myeloid and endothelial cells by CD40L gene therapy supports T-cell expansion and migration into the tumor microenvironment. Gene Ther.

[39] Rupp L, Dietsche I, Kießler M (2024). Neoadjuvant chemotherapy is associated with suppression of the B cell-centered immune landscape in pancreatic ductal adenocarcinoma. Front Immunol.

[40] Pearce EL, Mullen AC, Martins GA (2003). Control of effector CD8+ T cell function by the transcription factor Eomesodermin. Science.

[41] Llaó-Cid L, Roessner PM, Chapaprieta V (2021). EOMES is essential for antitumor activity of CD8^+^ T cells in chronic lymphocytic leukemia. Leukemia.

[42] Yan C, Richmond A (2021). Hiding in the dark: pan-cancer characterization of expression and clinical relevance of CD40 to immune checkpoint blockade therapy. Mol Cancer.

[43] Rodriguez-Rocha H, Gomez-Gutierrez JG, Garcia-Garcia A (2011). Adenoviruses induce autophagy to promote virus replication and oncolysis. Virology.

[44] Oh DS, Lee HK (2019). Autophagy protein ATG5 regulates CD36 expression and anti-tumor MHC class II antigen presentation in dendritic cells. Autophagy.

[45] Tan W, Liu M, Wang L (2021). Novel immune-related genes in the tumor microenvironment with prognostic value in breast cancer. BMC Cancer.

[46] Szadai L, Bartha A, Parada IP (2024). Predicting immune checkpoint therapy response in three independent metastatic melanoma cohorts. Front Oncol.

[47] Wong D, Yin Y (2023). Immune micro-environment analysis and establishment of response prediction model for PD-1 blockade immunotherapy in glioblastoma based on transcriptome deconvolution. J Cancer Res Clin Oncol.

[48] Klechevsky E, Flamar A-L, Cao Y (2010). Cross-priming CD8+ T cells by targeting antigens to human dendritic cells through DCIR. Blood.

[49] Ma H, Wu G, Chen H (2024). CLEC4A expression as a prognostic biomarker and immunoregulator in lung adenocarcinoma: insights from immune cell infiltration. MEDJ.

[50] Trimaglio G, Sneperger T, Raymond BBA (2024). The C-type lectin DCIR contributes to the immune response and pathogenesis of colorectal cancer. Sci Rep.

[51] Hansasuta P, Dong T, Thananchai H (2004). Recognition of HLA-A3 and HLA-A11 by KIR3DL2 is peptide-specific. Eur J Immunol.

[52] Li Y, Wu X, Sheng C (2024). IGSF8 is an innate immune checkpoint and cancer immunotherapy target. Cell.

[53] Zhu J, Jin A, Pan B (2024). Exploring the role of KIR3DL2 on NK cells in hepatocellular carcinoma and its potential prognostic implications. iScience.

[54] Park SD, Saunders AS, Reidy MA (2022). A review of granulocyte colony-stimulating factor receptor signaling and regulation with implications for cancer. Front Oncol.

[55] Bark SA, Dalmolin M, Malafaia O (2024). Gene Expression of *CSF3R*/CD114 Is Associated with Poorer Patient Survival in Glioma. Int J Mol Sci.

[56] Gungabeesoon J, Gort-Freitas NA, Kiss M (2023). A neutrophil response linked to tumor control in immunotherapy. Cell.

[57] Haraldsen G, Kvale D, Lien B (1996). Cytokine-regulated expression of E-selectin, intercellular adhesion molecule-1 (ICAM-1), and vascular cell adhesion molecule-1 (VCAM-1) in human microvascular endothelial cells. J Immunol.

[58] Afanasiev OK, Nagase K, Simonson W (2013). Vascular E-selectin expression correlates with CD8 lymphocyte infiltration and improved outcome in Merkel cell carcinoma. J Invest Dermatol.

[59] Xu Z, Pang TCY, Liu AC (2020). Targeting the HGF/c-MET pathway in advanced pancreatic cancer: a key element of treatment that limits primary tumour growth and eliminates metastasis. Br J Cancer.

[60] Mucciolo G, Araos Henríquez J, Jihad M (2024). EGFR-activated myofibroblasts promote metastasis of pancreatic cancer. Cancer Cell.

[61] Zhang R, Liu Q, Li T (2019). Role of the complement system in the tumor microenvironment. Cancer Cell Int.

[62] Wang Z-Y, Cheng J, Wang Y (2024). Macrophage ILF3 promotes abdominal aortic aneurysm by inducing inflammatory imbalance in male mice. Nat Commun.

[63] O’Reilly EM, Wainberg ZA, Hendifar AE (2026). Daraxonrasib or Chemotherapy in Previously Treated Metastatic Pancreatic Cancer. N Engl J Med.

[64] Orlen M, Vostrejs WP, Sor R (2025). T-cell Dependency of Tumor Regressions and Complete Responses with RAS(ON) Multi-selective Inhibition in Preclinical Models of Pancreatic Ductal Adenocarcinoma. Cancer Discov.

[65] Quevrin C, Mondini M, Meziani L (2026). RAS signaling at the crossroads of radioresistance and tumor immunity. J Immunother Cancer.

