# Supplementary material for: Immunostimulatory gene therapy with chemotherapy remodels pancreatic tumors and induces immune signatures associated with prolonged survival
Source: J Immunother Cancer. 2026 Oct 28;14(10):e016908. doi: 10.1136/jitc-2026-016908 (PMC13723919; doi:10.1136/jitc-2026-016908)
Supplement: Supplementary file 1 [file jitc-14-10-s001.pdf]

Supplementary Material

Immunostimulatory gene therapy with chemotherapy remodels pancreatic tumors and induces immune signatures associated with prolonged survival

Jessica Wenthe[1,2], Hanna Grauers Wiktorin[1], Amanda Hahn[1,3], Sedigheh Naseri[1], Ahmed Calandigary[1], Luke White[1], Linda C. Sandin[2], Clara Bernedal Nordström[2], Stephan Mielke[4,5], Tanja Lövgren[1], Gustav J. Ullenhag[1,3†], Angelica Loskog[1,2*†]

[1]Uppsala University, Department of Immunology, Genetics and Pathology, Uppsala, Sweden
[2]Lokon Pharma AB, Uppsala, Sweden
[3]Uppsala University Hospital, Department of Oncology, Uppsala, Sweden
[4]Department of Clinical Science, Intervention and Technology, Karolinska Institute
[5]Department of Laboratory Medicine, Karolinska University Hospital, Stockholm, Sweden

[*]Corresponding author: Angelica Loskog, Dept of Immunology, Genetics, and Pathology, Uppsala University. Dag Hammarskjöldsväg 20, 751 85 Uppsala, Sweden. Email: angelica.loskog@igp.uu.se.

[†]These authors contributed equally to this work

Table of Content

Supplementary Table S1: Nanostring statistical analysis (log2 fold change >1): Statistics were calculated using RUV-4 normalization with Benjamini-Hochberg correction for multiple tests. 3

Supplementary Table S2: Olink Immunology Panel: Multiple paired t-tests followed by False Discovery Rate (FDR) method of Benjamini and Hochberg (FDR=5%) 5

Supplementary Table S3: Olink Oncology Panel statistical analysis: Multiple paired t-tests followed by False Discovery Rate (FDR) method of Benjamini and Hochberg (FDR=5%) 8

Supplementary Table S4: Statistical analysis: Multiple paired t-tests followed by False Discovery Rate (FDR) method of Benjamini and Hochberg (FDR=5%) 11

Supplementary Table S5: Nanostring differential gene expression between pancreatic cancer patients based on median OS statistical analysis: Multiple paired t-tests 12

Supplementary Table S6: Nanostring differential gene expression between pancreatic cancer patients SD vs PR: Multiple paired t-tests 32

Supplementary Table S7: Olink in plasma differential expression between pancreatic cancer patients response and survival 52

Supplementary Figure S1: Flow cytometry gating strategy 84

Supplementary Figure S2: Proteins associated with immune stimulation and recruitment 85

Supplementary Figure S3: Proteins associated with T and NK cells 86

Supplementary Figure S4: Proteins associated with immunosuppression 87

Supplementary Figure S5: Proteins associated with blood vessels and angiogenesis 88

Supplementary Figure S6: Proteins associated with tumor biology 89

Supplementary Table S1: Nanostring statistical analysis (log2 fold change >1): Statistics were calculated using RUV-4 normalization with Benjamini-Hochberg correction for multiple tests.

Marker	Fold change week13 – week1	p-value post hoc BH
CCL5	2,059	1,09E-05
CTSS	1,9837	1,28E-04
CXCL9	1,9014	7,95E-04
GZMA	1,8936	9,90E-05
CCL18	1,8278	1,94E-02
HLA-DRA	1,7259	1,13E-04
HLA-DPB1	1,7166	7,95E-04
CD53	1,7087	7,21E-05
CYBB	1,6976	1,28E-04
GZMK	1,6383	1,03E-03
CXCL10	1,6377	2,37E-02
C1QB	1,6214	2,08E-03
ITGB2	1,5964	2,64E-04
GZMB	1,5816	8,15E-03
CD48	1,5635	1,28E-04
SLAMF7	1,5398	2,55E-04
GZMH	1,5317	2,46E-04
CCR5	1,5314	1,31E-04
CHIT1	1,5089	2,61E-02
CD74	1,5087	1,85E-04
HLA-DPA1	1,478	1,28E-04
HLA-DMA	1,4421	4,78E-06
COL3A1	1,4413	8,86E-03
PTPRC	1,4306	1,09E-05
FCGR3A	1,4209	2,12E-03
CD3D	1,3981	2,79E-04
C1QA	1,3853	5,10E-03
HLA-DMB	1,3851	1,59E-05
CXCR6	1,3845	1,03E-03
PRF1	1,3698	1,28E-04
TNFSF13B	1,3666	1,28E-04
S100A8	1,3443	7,75E-03
KLRK1	1,3427	6,98E-04
CD163	1,3409	4,73E-03
MSR1	1,3132	1,05E-03

HLA-DRB3	1,303	1,74E-04
CD8A	1,2877	2,34E-03
ITGAX	1,2699	1,31E-03
CXCR4	1,2636	6,82E-03
FCER1G	1,2465	5,36E-04
CD84	1,2373	1,28E-04
TARP	1,2345	1,28E-04
HLA-B	1,2157	3,38E-03
LCP1	1,2034	7,95E-04
IL2RG	1,1992	1,73E-02
IL7R	1,1872	3,55E-04
HLA-A	1,1797	3,38E-03
LILRB1	1,1625	1,74E-04
PSMB9	1,1556	6,70E-03
KLRB1	1,1484	3,52E-03
CR1	1,1469	2,08E-03
TLR4	1,1414	1,28E-04
GNLY	1,1387	3,49E-02
HLA-DQA1	1,1361	2,41E-02
STAT1	1,1339	4,81E-03
CD96	1,1283	1,12E-03
CD68	1,1239	7,75E-03
TNFRSF17	1,1129	1,92E-02
HLA-DQB1	1,0942	2,08E-03
LY96	1,0863	2,64E-04
SIGLEC1	1,0858	3,11E-03
CD27	1,0842	6,71E-03
LY86	1,0542	1,28E-04
CD3E	1,0488	3,50E-03
LY9	1,0487	9,15E-03
IRF4	1,0477	2,33E-02
CD8B	1,0409	1,31E-02
C3AR1	1,0361	2,12E-03
CCR1	1,0351	6,64E-03
INPP5D	1,0282	1,28E-04
HCK	1,0241	9,01E-04
ITGAL	1,016	1,70E-04
HLA-G	1,013	6,82E-03
C2	1,0089	4,99E-02

Supplementary Table S2: Olink Immunology Panel: Multiple paired t-tests followed by False Discovery Rate (FDR) method of Benjamini and Hochberg (FDR=5%)

Marker	log2 fold change w7	p-value (-log10) post hoc BH
Gal-9	0,4623	7,189
HO-1	0,6003	7,155
IL15	0,6241	6,789
IL18	0,6896	6,329
CCL17	-1,036	5,966
CXCL5	-0,8339	5,743
CX3CL1	0,4564	5,501
IL12	-1,153	5,461
PDGFB	-0,3974	4,735
ANGPT2	0,3351	4,264
CSF-1	0,1724	4,264
TNFRSF4	0,3749	4,192
ANGPT1	-0,5066	3,919
IL12RB1	0,3485	3,919
PD-L2	0,2475	3,502
CCL23	0,2682	3,191
TNFRSF21	0,1295	3,151
PGF	0,2664	3,115
EGF	-0,6192	3,017
TIE2	0,1608	2,943
LAG3	0,3102	2,943
MCP-3	0,3398	2,872
IFNγ	1,173	2,662
LAMP3	0,4138	2,662
CRTAM	0,315	2,575
CD4	0,1699	2,575
CD40L	-0,6293	2,57
CXCL10	0,5971	2,567
KLRD1	0,3357	2,567
FASLG	-0,3452	2,245
MCP-1	0,3481	2,137
CXCL11	0,3818	2,137
CXCL9	0,4537	2,137
CD5	0,205	2,137
MIC-A/B	0,166	2,103

CD8A	0,3208	2,058
TNF	0,3216	2,049
CAIX	0,3001	1,825
PD-L1	0,2152	1,825
MMP12	-0,3111	1,814
ADGRG1	0,2001	1,713
CCL3	0,2874	1,713
IL6	0,5111	1,673
MMP7	-0,07932	1,666
PDCD1	0,2097	1,496
TNFRSF12A	0,1578	1,422
NCR1	-0,1759	1,422
4-1BB	0,1522	1,377
CXCL13	0,2425	1,364
CD244	-0,1876	1,279
IL7	-0,2087	1,253
ADA	0,1656	1,253
TNFSF14	-0,2381	1,25
CD27	0,1158	1,217
GZMA	0,2079	1,098
TWEAK	-0,1466	1,092
CD83	-0,1402	1,091
CD70	0,1317	1,089
GZMB	0,2668	0,962
GZMH	0,4234	0,931
VEGFA	0,1375	0,9028
NOS3	0,1857	0,8873
MUC-16	0,2353	0,7891
IL8	0,1747	0,757
CASP-8	-0,1605	0,6688
VEGFR-2	0,05309	0,6486
CXCL1	-0,1036	0,5951
TRAIL	0,0751	0,5873
MCP-4	-0,09388	0,546
LAP TGF-beta-1	-0,0916	0,4881
HGF	0,05767	0,4556
CCL20	0,1922	0,3931
CD40	-0,05515	0,2858
IL10	0,05821	0,1389

DCN	0,007244	0,06442
CCL19	-0,01422	0,03172
CCL4	0,0105	0,02093
MCP-2	-0,005824	0,0187
ICOSLG	0,002579	0,0187
Gal-1	0,0001546	0,001323

Supplementary Table S3: Olink Oncology Panel statistical analysis: Multiple paired t-tests followed by False Discovery Rate (FDR) method of Benjamini and Hochberg (FDR=5%)

Marker	log2 fold change w7	p-value (-log10) post hoc BH
ITGAV	-0,1741	3,758
TNFSF13	0,2514	3,752
TNFRSF6B	0,5003	3,752
SYND1	0,4592	3,752
TNFRSF4	0,4084	3,752
CD207	-0,4583	3,752
5'-NT	0,3805	3,212
EGF	-0,5983	2,686
CTSV	-0,2188	2,686
DLL1	0,1942	2,686
EPHA2	0,2671	2,553
SCF	-0,3945	2,544
WFDC2	0,18	2,544
FR-alpha	0,2232	2,544
FCRLB	0,2789	2,519
TGFR-2	0,2008	2,462
FASLG	-0,3526	2,462
MIC-A/B	0,1729	2,121
LYPD3	-0,1218	2,089
CD27	0,1548	2,049
WISP-1	0,1897	2,006
RSPO3	0,1614	1,882
CAIX	0,3313	1,862
TFPI-2	0,2311	1,688
ERBB3	0,09626	1,661
TNFRSF19	0,211	1,599
GPC1	0,1221	1,57
S100A11	0,1526	1,57
TXLNA	0,274	1,532
MSLN	0,2639	1,512
IL6	0,5187	1,424
VEGFR-3	0,1102	1,424
PVRL4	0,1398	1,406

ITGB5	-0,1799	1,406
CD70	0,1892	1,379
SCAMP3	-0,3507	1,375
hK11	0,09669	1,343
CD48	0,08987	1,322
VEGFA	0,1751	1,238
CXCL13	0,2181	1,234
LYN	0,1278	1,198
IFNgR1	0,06788	1,186
CRNN	-0,2059	1,158
CPE	0,1029	1,133
LY9	0,117	1,073
FGF-BP1	-0,1385	1,014
CEACAM1	0,05041	0,9851
GZMH	0,4603	0,9794
CXL17	0,1185	0,9794
MK	0,1285	0,9062
ADAM-TS 15	0,1191	0,8325
AREG	0,1613	0,8011
TRAIL	0,08989	0,7086
S100A4	-0,1321	0,7086
PPY	0,2121	0,7086
hK8	-0,09016	0,6645
MetAP 2	-0,1637	0,6109
CD160	-0,08401	0,6109
KLK13	0,1092	0,602
MUC-16	0,2102	0,601
TLR3	0,08356	0,5915
VEGFR-2	0,05549	0,5911
XPNPEP2	0,05279	0,5005
ESM-1	-0,07441	0,4791
CDKN1A	-0,1681	0,4184
ADAM 8	-0,08252	0,396
HGF	0,06338	0,3835
GZMB	0,163	0,3699
TCL1A	-0,183	0,3412
FR-gamma	0,04726	0,3309
IGF1R	0,05099	0,3089
Gal-1	0,02747	0,2239

ERBB4	-0,03445	0,217
ABL1	-0,1048	0,2159
VIM	-0,1211	0,202
MAD homolog 5	0,03251	0,1626
RET	-0,0262	0,1185
SPARC	-0,01335	0,09755
TGF-alpha	-0,03042	0,09755
CYR61	0,02225	0,09755
CEACAM5	-0,06021	0,09755
ANXA1	-0,0356	0,09755
ERBB2	-0,01355	0,09265
PODXL	-0,003933	0,02356
FURIN	-0,006016	0,02356
hK14	0,006933	0,02356
GPNMB	0,004821	0,02356
MIA	0,003168	0,02356
ICOSLG	-0,003008	0,02356
SEZ6L	-0,001715	0,01101
WIF-1	0,001862	0,01101

Supplementary Table S4: Statistical analysis: Multiple paired t-tests followed by False Discovery Rate (FDR) method of Benjamini and Hochberg (FDR=5%)

	P value	Mean of > Median OS	Mean of ≤ Median OS	Difference	SE of difference	q value	-log10(q value)
PVRL4	0,00014	5,242	5,983	-0,7408	0,1744	0,004209	2,376
VEGFR-3	0,001221	7,225	7,422	-0,1966	0,05614	0,018319	1,737
CD70	0,003671	3,812	4,251	-0,4395	0,1417	0,036706	1,435
SYND1	0,005817	7,91	8,611	-0,7005	0,2393	0,038346	1,416
WISP-1	0,006391	6,431	6,79	-0,3586	0,1241	0,038346	1,416
TNFRSF6B	0,007773	5,12	5,683	-0,5638	0,2003	0,038867	1,41
TXLNA	0,016954	2,839	3,379	-0,5404	0,2161	0,056514	1,248
EPHA2	0,013302	3,77	4,161	-0,3906	0,1502	0,056514	1,248
S100A11	0,016805	3,686	4,013	-0,3268	0,1305	0,056514	1,248
5'-NT	0,020877	10,55	11,1	-0,5497	0,2278	0,06263	1,203
LY9	0,072163	5,687	5,953	-0,2659	0,1437	0,160758	0,7938
ERBB3	0,077357	8,238	8,403	-0,1655	0,09111	0,160758	0,7938
CAIX	0,072492	4,864	5,432	-0,5684	0,3074	0,160758	0,7938
CD27	0,080379	7,937	8,184	-0,247	0,1374	0,160758	0,7938
DLL1	0,079251	10,85	11,05	-0,206	0,1141	0,160758	0,7938
IL6	0,153116	4,174	4,845	-0,6711	0,4601	0,275991	0,5591
FCRLB	0,156395	2,196	2,438	-0,2424	0,1675	0,275991	0,5591
MIC-A/B	0,184497	4,685	5,295	-0,6099	0,451	0,307495	0,5122
FR-alpha	0,244142	9,14	9,297	-0,1572	0,1329	0,385488	0,414
WFDC2	0,278133	7,249	7,41	-0,161	0,1462	0,417199	0,3797
CD48	0,308658	6,839	6,953	-0,1146	0,111	0,44094	0,3556
hK11	0,34018	4,876	4,995	-0,1199	0,1241	0,463882	0,3336
TNFSF13	0,392505	8,891	8,984	-0,09298	0,1075	0,511964	0,2908
TGFR-2	0,500273	7,424	7,52	-0,096	0,141	0,577238	0,2386
TFPI-2	0,494415	8,019	8,166	-0,1462	0,2118	0,577238	0,2386
TNFRSF19	0,491846	5,639	5,775	-0,1359	0,1957	0,577238	0,2386
MSLN	0,592817	4,171	4,304	-0,1324	0,2455	0,635161	0,1971
GPC1	0,576115	4,56	4,63	-0,07015	0,1244	0,635161	0,1971
TNFRSF4	0,840607	6,721	6,757	-0,03681	0,1818	0,869594	0,06068
RSPO3	0,887396	5,958	5,937	0,02091	0,1467	0,887396	0,05188

Supplementary Table S5: Nanostring differential gene expression between pancreatic cancer patients based on median OS statistical analysis: Multiple paired t-tests

Marker	log2 fold change	p-value (-log10) post hoc BH
SPP1	3,002	1,4508128
CCL15	2,179	1,4507882
FPR2	2,07	2,5353614
TNFRSF13B	1,884	1,4222211
S100A12	1,832	1,755253
IL1RN	1,68	1,5451399
CAMP	1,563	1,3305901
IL27	1,513	1,6726411
TNFRSF10C	1,468	1,4241658
IL1R2	1,331	1,5246921
KIR3DL2	1,298	1,3790108
IL21	1,256	1,6673394
CSF3R	1,238	2,2987775
IFNG	1,172	2,2077484
ITGAX	1,172	1,5563772
SPANXB1	1,159	1,3111265
TNF	1,121	1,6819993
RELB	0,9772	1,304177
EOMES	0,9742	1,3437772
SH2B2	0,9709	1,8228099
CLEC6A	0,944	2,0600317
CLEC4A	0,8344	1,4976958
CD40	0,8243	1,8808101
RUNX3	0,8169	1,8344886
TBX21	0,8138	1,438374
MTMR14	0,7887	1,7785075
IL18	0,7607	1,5846927
PPARG	0,7567	1,3860525
LYN	0,7371	1,8861569
TNFRSF10B	0,7253	1,818185
NUBP1	0,7208	1,3743481
LCK	0,6738	1,5909634
TRIM39	0,582	1,3370911
RIPK2	0,553	1,4612629

ATG5	0,5131	1,5181562
ILF3	-0,5361	1,3804997
ITGB1	-0,6836	1,3125335
APP	-0,9372	1,6176587
CD99	-0,9467	1,6254899
FEZ1	-1,263	2,1075158
C1S	-1,634	1,3272189
C1R	-2,146	1,507519
C4B	-2,207	1,793795
CCL21	-2,593	1,3202542
C3	-3,226	1,4523976
CD7	0,713	1,2910695
IL21R	1,431	1,2608946
CREBBP	0,7775	1,2542462
IL9	1,179	1,253833
LRP1	-1,391	1,2453612
LY96	0,5826	1,2349427
NFKB2	0,5368	1,2335723
ENG	-1,31	1,2304824
CD9	0,7023	1,2288169
PRKCD	0,7556	1,2253006
CCL23	1,005	1,2091662
PDGFRB	-1,321	1,2054144
CD200	-1,15	1,1981437
CD81	-0,8429	1,1936369
LILRA1	0,7581	1,1761868
CXCR4	0,8487	1,1741781
IFI16	-1,01	1,1712241
CD37	0,659	1,1677402
ITGA1	-1,058	1,1585717
CXCL12	-1,765	1,1575405
S100A8	1,528	1,1519588
CCRL2	0,84	1,1472517
PYCARD	0,7179	1,1358629
IKBKE	0,6975	1,1350027
TFRC	1,511	1,1341856
IL12RB1	0,525	1,1210304
PECAM1	-0,871	1,1208238
CLEC5A	1,61	1,1153009

IL13RA2	0,9179	1,113814
CASP3	0,5065	1,1049257
IFNA1	1,207	1,1023289
CSF2RB	0,7709	1,1020597
BCL10	0,5515	1,0987761
CXCR2	1,109	1,0866419
MAVS	-0,7593	1,0841888
IL17RA	0,7612	1,0671459
MFGE8	-1,01	1,0643668
MAGEA1	1,337	1,049903
CXCR6	1,068	1,043289
AXL	-0,7577	1,0421725
CCL25	0,8246	1,0399242
PRAME	0,8387	1,0322883
REL	0,5741	1,0302394
TLR10	0,6286	1,0167211
MAP2K1	0,4122	1,0081628
MAP2K2	-0,4307	1,0004997
A2M	-1,454	0,9929012
CR2	1,116	0,98799
IL8	2,347	0,9845438
SLC11A1	1,776	0,9807923
EGR2	1,098	0,9753584
PSEN1	0,3441	0,974981
EGR1	-0,7338	0,9742682
POLR2A	-0,4422	0,9638983
IFNA17	0,9067	0,9594807
BTK	0,542	0,9586942
FADD	0,6914	0,9584179
CXCR1	0,9121	0,9411604
SYT17	0,8558	0,9306465
THBS1	-1,215	0,9304392
HAVCR2	1,231	0,9269969
ST6GAL1	-0,9596	0,9218732
CD19	0,9637	0,9203124
TXNIP	-0,9826	0,9168846
CREB1	-0,3482	0,9063052
EWSR1	-0,4443	0,9011037
SH2D1B	1,016	0,8928884

BID	0,9899	0,8867287
HLA-DRB4	1,157	0,885379
C1QA	-1,077	0,8812592
GATA3	1,038	0,8808728
MEFV	1,216	0,872798
IL13	0,6901	0,8721698
IL1B	1,272	0,8668189
NRP1	-0,8596	0,8631146
FCGR1A	0,8072	0,8526082
IL18RAP	0,888	0,8473448
VEGFC	-0,8213	0,8404148
IL6ST	-0,9788	0,8387638
CTAG1B	0,825	0,8372296
F13A1	-1,847	0,8364153
TNFRSF14	0,5189	0,830746
ATM	1,212	0,8255884
IRF5	0,7711	0,8240767
IRF8	0,5657	0,8186338
TLR8	0,6087	0,8164939
JAM3	-1,072	0,8110603
NCF4	0,5098	0,8105995
IRF1	0,4183	0,8056411
IFNAR1	0,3903	0,8052609
THY1	-1,101	0,7993766
ADA	0,4097	0,7979806
XCL2	0,6016	0,7976261
NLRC5	0,7072	0,7975035
CMKLR1	-0,5593	0,796445
CD244	0,5907	0,7937923
TNFAIP3	0,5341	0,7927832
IFNAR2	0,3586	0,7922849
CCL3L1	1,513	0,7920696
STAT3	-0,5799	0,7868144
HLA-DPA1	-0,8459	0,7860628
CCL3	1,333	0,7847432
IFI35	-0,5195	0,7810393
PAX5	0,7875	0,773854
TNFSF11	0,6391	0,769408
STAT2	-0,5902	0,7693748

TNFRSF1B	0,4795	0,7647115
PPBP	1,805	0,7612315
CD74	-0,9622	0,759603
IRAK2	0,6465	0,7567858
SERPING1	-1,15	0,7549787
PASD1	0,6543	0,7541346
SELE	0,9282	0,7517496
FCF1	-0,4793	0,7484372
CFI	-1,191	0,7411234
KIR_Activating_Subgroup_1	0,9831	0,7380951
ITK	0,7596	0,7376439
PRM1	0,8369	0,7376082
CARD9	0,7864	0,7354797
CFB	-1,015	0,7345501
TFEB	0,576	0,7316889
AMICA1	0,5634	0,7279866
IL6R	0,6718	0,7277406
MAP3K1	0,4025	0,72599
ZNF143	0,5667	0,7247048
IL23R	0,8234	0,723552
CCL4	1,16	0,7210979
CHIT1	1,847	0,7202327
BATF	0,537	0,7194876
ITGAE	0,5472	0,7145305
COL3A1	-0,9782	0,714157
IL2RG	0,9327	0,7133101
PLA2G6	0,6601	0,7108259
IRF2	0,5017	0,7102951
NOS2A	1,274	0,7093445
NT5E	-0,8483	0,7090488
G6PD	0,5693	0,7070554
FCER2	0,6975	0,6993458
IL17F	1,094	0,6966981
ALAS1	0,5563	0,6943735
CCL7	0,7777	0,682689
CD3G	0,5184	0,6822959
CD163	-1,118	0,6821204
BMI1	-0,4341	0,6807586
CD3D	0,5565	0,6781345

CARD11	0,4476	0,6769563
CD79B	0,6845	0,6760697
IL1RL2	0,7563	0,6732833
PLAUR	1,16	0,6696146
HLA-DQA1	-1,265	0,6655885
POU2AF1	0,8109	0,6646745
LAIR2	0,6103	0,6645662
TFE3	0,5798	0,6638546
TNFRSF4	0,7634	0,6624769
CTLA4	0,7069	0,6620039
CD8A	0,6806	0,6611711
DOCK9	-0,3345	0,6592962
CCL28	1,8	0,6580217
CD1B	0,871	0,6574628
CSF1	-0,5401	0,6526837
ZC3H14	-0,2531	0,6515218
KIR_Inhibiting_Subgroup_2	0,8807	0,6408516
TREM1	1,541	0,6395107
FYN	-0,3954	0,6389468
PIN1	-0,4068	0,6376966
TP53	-0,4641	0,6373159
CTSH	0,6698	0,633004
AICDA	0,9278	0,6327932
TBK1	0,2729	0,6315327
CCL20	0,983	0,6287215
TICAM1	0,3529	0,6270972
IL6	0,8381	0,6203537
TNFSF13	0,4849	0,6170002
IL17RB	1,15	0,6146351
C7	-1,93	0,6123806
SH2D1A	0,7073	0,6122134
NCAM1	-0,8269	0,6116892
BST2	-0,6953	0,604602
ELANE	0,5819	0,5980466
CD1A	0,6195	0,5958251
CXCR3	0,6322	0,5949594
CSF2	0,8805	0,5946366
IL18R1	0,4936	0,5915769
CYFIP2	0,8536	0,5915667

ICAM2	-0,3555	0,5859514
CLEC7A	0,6117	0,5844958
TUBB	-0,5705	0,5828722
CD70	0,8312	0,5813093
PPIA	0,3222	0,5808823
C1QB	-1,098	0,5798329
IL1RL1	0,5691	0,5791947
IFNB1	0,6612	0,5774266
MS4A1	1,353	0,5751918
ATF2	-0,2475	0,5734399
BLK	0,7495	0,5701412
PSMB10	0,3679	0,5699459
SPO11	0,9026	0,5677605
SEMG1	0,5345	0,5620355
GNLY	0,8354	0,5563225
ITGAM	0,6855	0,5531181
SLAMF7	0,6099	0,5511144
CEACAM1	0,5415	0,5478316
APOE	1,119	0,5477979
CD3EAP	-0,3428	0,5475695
ATG10	0,6235	0,5471988
NFATC3	0,2888	0,5460215
SBNO2	0,3664	0,5446617
IL1R1	-0,6154	0,5435854
TLR1	0,3372	0,5431771
IFNL2	0,6422	0,5427541
TREM2	0,9169	0,5414437
CD22	0,8796	0,5395547
FOS	0,6185	0,5392525
ITGA2B	1,14	0,5386005
TNFSF4	-0,5334	0,5380665
NEFL	0,9482	0,534143
DDX50	0,3626	0,5340494
USP9Y	0,762	0,5305359
IL10	0,6011	0,5301162
CDH1	-1,568	0,5263904
SLAMF1	0,7385	0,5260505
CD58	0,3564	0,5246688
TNFRSF17	0,8419	0,5231843

ATG16L1	-0,3574	0,5217973
ETS1	-0,4632	0,5213397
HLA-DRB3	-0,5932	0,520923
SPN	0,4976	0,5194412
TNFRSF18	0,7826	0,5166381
GZMB	0,9068	0,5162844
IL17A	0,6635	0,5151608
ABCF1	-0,2797	0,5089901
MICA	0,5362	0,5073625
CD5	0,431	0,5061364
CD48	0,5221	0,5047846
MAGEA12	0,6969	0,5004105
CASP1	0,323	0,4994603
BIRC5	-0,736	0,4983972
MAPK11	0,7284	0,4949953
PDGFC	-0,472	0,4926259
TNFRSF8	0,6428	0,4812374
CTSL	0,7546	0,4799648
IL10RA	-0,3312	0,4793226
CKLF	0,2849	0,4769458
THBD	-0,5019	0,4741651
IL12B	0,7937	0,4740694
IL4	0,6391	0,4707288
TBP	0,3644	0,470707
IGLL1	0,588	0,4690553
CCR1	0,5952	0,4686833
LILRB3	0,6545	0,466907
POU2F2	0,3334	0,466031
KIR3DL1	0,9455	0,46511
MASP1	0,5149	0,464559
MAPKAPK2	0,2303	0,4616148
LILRA5	0,4148	0,4612127
ITGB3	0,8518	0,4611424
TPSAB1	-0,7736	0,4607658
IL15RA	0,2603	0,4548265
MAGEA4	0,7621	0,4531071
ITGA6	-0,4883	0,452613
MAPK3	-0,3667	0,4478321
NFATC1	-0,2774	0,447226

SOCS1	0,6661	0,4448523
ATF1	0,2297	0,4444098
DPP4	0,6811	0,4441417
PTGS2	1,033	0,4437711
NCR1	0,4938	0,442831
CCL19	-1,569	0,4427275
CD3E	0,466	0,4426998
TICAM2	0,3536	0,442423
SIGLEC1	-0,5982	0,4421332
CEACAM6	-1,895	0,4417259
HLA-DPB1	-0,8446	0,4414378
IL13RA1	-0,2902	0,4410372
FUT7	0,539	0,4396653
TAP2	0,3635	0,4394096
IL22RA2	0,3721	0,4373789
GTF3C1	-0,2509	0,4358857
ICAM1	0,4823	0,4358692
ARG2	0,6698	0,4347722
TNFSF12	-0,6683	0,4339115
SMAD2	0,3019	0,4338113
C9	-1,186	0,4324796
CD14	-0,6883	0,4291816
LAMP3	-0,6769	0,4273259
BCL2L1	0,278	0,4263372
ANP32B	-0,2757	0,4221407
HLA-DMB	0,3325	0,4196465
MIF	0,3806	0,4186774
CDH5	-0,4672	0,4183086
IRF4	0,6193	0,4172755
C2	0,6554	0,4162743
IL15	0,4924	0,4159052
FN1	-0,9231	0,413586
CXCL14	-1,1	0,4129528
ZKSCAN5	0,4024	0,4128426
CTAGE1	0,4127	0,4128146
IFIT1	-0,6732	0,4119458
KIR_Inhibiting_Subgroup_1	0,6369	0,4030699
PRPF38A	-0,2222	0,4025988
EDC3	-0,3339	0,401793

HDAC3	0,216	0,4011614
BST1	0,5563	0,4010083
IL12RB2	-0,8343	0,4003527
MICB	0,3964	0,3972284
IL17B	0,6703	0,394515
LGALS3	0,8015	0,3941166
TNFSF13B	-0,3593	0,3937692
TIGIT	0,351	0,3880124
CD59	-0,3733	0,3854497
HCK	0,4534	0,3810288
LAG3	0,6807	0,3791563
ITGB4	-0,8079	0,3782101
ROPN1	0,428	0,3769741
IL7	0,4865	0,3761287
ISG20	0,3509	0,3748924
CEACAM8	0,3586	0,3735262
IFITM2	-0,3836	0,3733405
MS4A2	-0,6581	0,3685735
CXCL11	-0,7475	0,3682276
TARP	0,3271	0,3663225
MPPED1	-0,5315	0,3661439
CXCL13	0,7881	0,3637388
IKBKB	0,3298	0,3631121
AKT3	-0,3337	0,3621572
LTK	0,5696	0,3598248
MAP4K2	0,3662	0,3592563
TRAF3	-0,2228	0,3589177
KIR3DL3	0,4639	0,3570352
CXCR5	0,5605	0,3555102
TNFSF15	0,6832	0,3530339
CCL8	0,757	0,3524039
CD1D	-0,4488	0,3515668
TLR9	0,2472	0,3513912
CD53	0,4107	0,3484623
HLA-C	-0,3452	0,347745
CT45A1	0,5	0,3453144
GAGE1	0,4209	0,3440571
KLRC1	0,3394	0,3437829
SSX4	0,6455	0,3432245

F12	-0,5871	0,3410256
MARCO	1,383	0,3409599
FOXP3	0,4818	0,3403005
DEFB1	0,8745	0,3399394
ARG1	-1,286	0,3384022
HLA-DQB1	-0,3861	0,3382668
EP300	-0,1306	0,3379414
IFIT2	-0,4819	0,3374084
CD4	-0,3519	0,3363622
RPS6	-0,3341	0,3360514
IL19	0,6109	0,3356768
IL2	0,5501	0,3351052
SAA1	-1,123	0,3314464
CD247	0,3046	0,3305018
CSF3	0,727	0,3290745
MR1	0,3457	0,3282553
ULBP2	0,5296	0,3281868
PVR	0,247	0,3269699
CASP8	-0,2222	0,3265091
ABL1	-0,196	0,3223401
CD36	-0,8847	0,3213642
CSF1R	0,3007	0,3200436
C8A	-0,7911	0,3198976
CDK1	-0,5226	0,3197787
RUNX1	-0,3003	0,3187072
BLNK	0,4239	0,3187027
TPTE	-0,2751	0,318322
IRF3	0,2901	0,3143138
C4BPA	-1,117	0,3138797
CD276	-0,1991	0,3078663
ITCH	-0,2855	0,3074263
TNFRSF11A	0,5105	0,3071337
TAP1	0,3991	0,3053501
IL3	0,4102	0,3041849
EBI3	-0,3438	0,3041569
TNFSF8	0,3672	0,3018159
IGF2R	0,272	0,3015767
IL24	0,4116	0,301488
CFD	-0,654	0,2995576

LILRB1	0,3125	0,2987378
MEF2C	-0,278	0,2977143
CXCL1	0,5846	0,2972878
C5	0,5085	0,2961148
CD83	0,4091	0,2940149
CFP	0,4092	0,2935357
ATG7	0,1622	0,2913183
RAG1	0,3972	0,2897252
TNFRSF1A	-0,2381	0,2890436
MAPK1	-0,1252	0,2874934
IKBKG	-0,2715	0,2865691
IL7R	-0,326	0,2858656
C6	-1,161	0,2851574
ZNF346	0,3166	0,2850528
MAGEC2	-0,2667	0,2843292
HLA-DRA	-0,4576	0,2837971
CXCL16	0,3378	0,2827732
KLRD1	0,38	0,2826641
HLA-DOB	0,2518	0,2818572
PLA2G1B	-1,278	0,2815124
CD86	0,3761	0,2810427
TRAF2	0,2565	0,2797811
BAGE	0,3257	0,2782026
IL1RAPL2	0,2757	0,2762702
MRPS5	0,1689	0,2762431
DDX58	-0,3466	0,2759971
CD46	-0,3479	0,2731628
ITGAL	0,1776	0,2727695
FCGR2B	0,4678	0,2715115
IL5	0,5128	0,2711951
TLK2	0,1656	0,2694939
GPATCH3	0,1922	0,2691523
NOD1	-0,2218	0,2691096
CTSW	0,32	0,2690458
SSX1	-0,4749	0,268313
PSMD7	-0,2331	0,2649827
JAK2	-0,2122	0,2646487
MAPK8	0,2695	0,2640859
AMBP	-1,384	0,2635868

FCER1G	0,4113	0,2619484
DUSP4	0,3794	0,2618372
IRAK1	-0,177	0,2604071
SAP130	-0,198	0,2602711
SELL	0,4801	0,2592032
TLR2	0,2708	0,2551409
CCR3	0,5292	0,2550854
LILRA4	-0,371	0,2533183
CCL5	0,2387	0,2529216
IL11RA	-0,3574	0,2527444
S100B	-0,5026	0,2522116
PRG2	-0,3544	0,2488772
NOTCH1	-0,2795	0,2484107
TCF7	-0,2701	0,2452214
STAT4	0,2666	0,2443561
TNFRSF11B	0,4619	0,2436081
IRAK4	-0,1942	0,2384014
CD8B	0,3478	0,2373792
MCAM	-0,4404	0,2371265
MME	0,6342	0,2362427
JAK1	-0,1867	0,235525
MAGEB2	0,3913	0,2353039
CCL17	-0,366	0,2349323
IFITM1	-0,4582	0,2331769
NFKB1	0,2485	0,2324204
DHX16	0,252	0,2316424
IFNA2	0,444	0,2315773
CYLD	0,1555	0,2276203
CCL11	-0,4384	0,2275888
PIK3CG	-0,2195	0,2266539
CCL2	0,3991	0,2246532
ITGA5	-0,2951	0,2242432
SYK	0,192	0,222529
CCL22	-0,2746	0,2202355
ICAM3	0,2108	0,2175102
IFI27	-0,6464	0,2160243
CYBB	-0,3841	0,2116061
FUT5	0,3248	0,2115636
IL22	0,5361	0,2113467

CD68	0,4083	0,2108919
TNFSF18	-0,3367	0,2085185
C8B	-0,5534	0,208256
C8G	-0,4217	0,2073975
MAP3K5	0,1171	0,2073065
TIRAP	0,1117	0,2060532
IRGM	0,2436	0,2048481
HLA-DMA	-0,1767	0,2046331
CXCL3	0,4876	0,2026189
MAPK14	0,1202	0,2015234
MAGEA3	0,2777	0,199791
IDO1	0,2753	0,1986292
CD160	0,3178	0,1977566
TAPBP	-0,2497	0,1968974
KLRG1	0,3448	0,1960686
GPI	0,1888	0,1958988
FAS	0,2084	0,1957801
LILRB2	0,1813	0,1954136
SELPLG	0,1966	0,1953584
CCL1	-0,2869	0,1941282
MRC1	-0,3586	0,193934
ZAP70	0,182	0,190955
CD47	0,263	0,190093
ADORA2A	0,2271	0,1898878
CD33	0,2436	0,1890414
IL22RA1	0,3426	0,1890252
IL12A	0,2004	0,1886885
REPS1	0,1522	0,1877352
CD274	0,3454	0,1861036
FCER1A	-0,5328	0,1860836
PTPRC	-0,1586	0,1842483
CD44	0,2443	0,1828255
CD79A	0,31	0,182029
ITGB2	-0,3148	0,1812992
EPCAM	-0,6693	0,181112
ICOSLG	0,1335	0,1800246
SPACA3	-0,2442	0,1799142
FCGR3A	0,3962	0,1791827
HPRT1	0,2244	0,1783784

IL1A	-0,3653	0,1773188
CCL24	0,4901	0,1756841
KLRC2	0,2888	0,1746343
CD96	0,1872	0,1740192
CR1	0,2599	0,1736498
NFKBIA	0,1991	0,1725332
KLRF1	0,3484	0,1725144
CCND3	-0,1027	0,1706415
INPP5D	0,1649	0,1638241
MX1	-0,3217	0,1634879
HLA-B	-0,242	0,162139
SPA17	0,2778	0,1620273
TMUB2	-0,1662	0,1613643
AIRE	0,2155	0,1592887
MNX1	0,4332	0,1587425
TMEFF2	0,2134	0,1575337
TNFRSF13C	0,3451	0,1571163
CCR4	0,2914	0,1563711
TNFSF10	-0,3488	0,1517585
ELK1	0,1153	0,1506359
CCL13	-0,4025	0,1505394
MAF	-0,1661	0,1493905
LTB	0,293	0,149219
XCR1	0,2081	0,1483222
IL32	-0,2669	0,1460513
DUSP6	0,1761	0,1458434
LY86	0,1265	0,1446438
CXCL5	0,5143	0,1441363
ATG12	0,2188	0,1440752
TTK	-0,2333	0,1438447
LAMP1	-0,131	0,1432059
LBP	-0,4699	0,1410125
SF3A3	-0,08553	0,1391675
PMCH	0,2154	0,1388457
STAT6	-0,06789	0,1381145
HLA-G	0,197	0,1372921
GZMK	-0,1931	0,1370718
GZMA	0,1682	0,1370485
RORC	-0,2761	0,1360672

PLAU	-0,2067	0,1340874
TNFRSF12A	-0,204	0,1331104
CD38	-0,3172	0,1321167
CREB5	0,1994	0,1310518
CD34	-0,2576	0,1309826
SMPD3	0,2894	0,1307472
PRF1	0,1379	0,1302105
MAP2K4	-0,09954	0,1293661
ENTPD1	0,2109	0,1280402
IL26	0,1597	0,1274493
CD24	-0,5694	0,1271715
CD63	0,1707	0,127054
CEBPB	0,2893	0,1268783
CD164	-0,1626	0,1267759
OAS3	-0,2658	0,1266806
LRRN3	-0,2459	0,1264353
JAK3	0,1434	0,1261129
MASP2	-0,2656	0,1259858
IL11	0,4285	0,1259411
CXCL2	0,2586	0,1246094
PRKCE	0,2238	0,1245805
CMA1	-0,305	0,1231941
ZNF205	0,1697	0,122312
CHUK	0,08812	0,1219139
PDCD1LG2	-0,1967	0,1209288
ANXA1	-0,2589	0,1188454
ISG15	-0,329	0,1182166
BAX	0,09467	0,1180006
CCL27	-0,1731	0,1171984
MUC1	-0,384	0,1145658
CD207	0,1943	0,1138625
FLT3	0,2012	0,1124063
CTSS	0,2659	0,1120931
IL2RA	0,2032	0,1115038
LTA	-0,1432	0,1106064
HSD11B1	-0,2607	0,1096589
IL2RB	-0,1015	0,108564
HAMP	-0,3161	0,1081783
CD27	0,1615	0,1080524

ICOS	-0,1747	0,1064041
HRAS	0,12	0,1037483
RELA	0,06325	0,1024328
MERTK	0,1338	0,1006359
TNFSF14	0,08901	0,1002971
TAL1	0,2234	0,0988311
RORA	-0,1576	0,0981773
CD1C	0,2695	0,0980472
FCGR2A	0,1523	0,0978497
CCL18	0,4365	0,0966734
PDCD1	0,1583	0,0964065
NLRP3	0,1047	0,0961767
GUSB	0,07657	0,095298
IL3RA	0,2014	0,0945593
LIF	-0,1796	0,0943433
ECSIT	0,1109	0,0942673
CD84	0,1224	0,091825
LTBR	-0,0757	0,0905451
CTCFL	0,1598	0,0894124
ITGA4	-0,09762	0,0889863
IL16	0,1012	0,0883354
GZMM	0,1025	0,0877779
CXCL9	0,2004	0,0875643
PSMB8	-0,1181	0,0842394
KLRB1	0,1585	0,0839832
NFATC4	-0,1429	0,0838383
PSMB7	-0,0842	0,0831924
COG7	-0,08224	0,0822094
MAGEC1	0,1182	0,0790881
IL4R	-0,143	0,0783026
DNAJC14	0,08034	0,0780613
MAP3K7	-0,06724	0,0773731
TLR7	-0,1365	0,077314
IFIH1	0,1133	0,0769794
COLEC12	-0,2252	0,0740071
CNOT4	-0,08216	0,0733803
CCR6	-0,1067	0,0731238
MYD88	0,08743	0,072854
PIK3CD	-0,06586	0,0721868

CCL14	-0,2343	0,0714654
CC2D1B	0,1193	0,0688774
CDKN1A	0,0691	0,0685732
MSR1	-0,1266	0,0673989
LY9	-0,0908	0,0668728
NUP107	0,05809	0,0665051
SDHA	-0,07368	0,065446
TYK2	-0,06018	0,0647815
EIF2B4	0,06497	0,0640189
HLA-A	-0,1059	0,0619709
VCAM1	0,1234	0,0590873
PSMB9	0,1004	0,0583579
CNOT10	0,04819	0,0578356
C3AR1	0,08493	0,057795
CXCL10	-0,1998	0,0569895
FOXJ1	0,1746	0,0567375
TXK	-0,1099	0,052385
CD40LG	0,0605	0,0521998
PTGDR2	-0,1497	0,0516521
LTF	-0,2138	0,0510279
SYCP1	-0,06736	0,0503183
SMAD3	0,04566	0,0501369
PNMA1	0,04466	0,0479582
KIR_Activating_Subgroup_2	0,1021	0,0477434
LAMP2	-0,05319	0,0465559
STAT1	-0,1052	0,0464312
NOL7	-0,03861	0,0460809
KIT	0,1156	0,0456793
CCR5	0,02846	0,0440473
SLAMF6	0,05873	0,0437273
CRP	0,2262	0,0428268
CX3CR1	0,1034	0,0427304
RRAD	-0,1397	0,0407921
SIGIRR	-0,06198	0,039523
DDX43	-0,0993	0,039338
IFNGR1	-0,04307	0,0391816
TLR4	0,03573	0,0390201
NFATC2	-0,02325	0,0379599
CCL16	-0,1116	0,0375563

CD55	-0,06835	0,0373617
CX3CL1	0,06101	0,0371067
IRF7	-0,06168	0,0366335
LCN2	0,1649	0,0353355
TLR3	-0,06321	0,0352347
IL1RAP	-0,05085	0,0348199
NOD2	0,06795	0,0346172
CTSG	0,08536	0,0325733
YTHDF2	0,02005	0,0317053
CCL26	-0,08754	0,0312668
AMMECR1L	-0,03015	0,0305258
VEGFA	0,06046	0,0304387
MST1R	0,1109	0,0298252
TLR5	-0,05136	0,0293366
IL23A	0,05518	0,029239
CLEC4C	0,07213	0,0291461
ERCC3	0,02535	0,0288137
CD2	-0,04172	0,0280616
BCL2	0,05404	0,0278324
CD180	0,02763	0,0275727
HMGB1	0,02019	0,0273881
FLT3LG	0,03906	0,0272669
ALCAM	0,04248	0,0269604
AGK	-0,02888	0,0260777
TGFB2	-0,06438	0,0255242
SPINK5	-0,04605	0,0253267
TAB1	0,03008	0,0252535
IL25	0,04755	0,0252517
ABCB1	-0,08606	0,0252245
TNFRSF9	0,05235	0,0244956
GZMH	-0,03388	0,0237124
TRAF6	-0,01738	0,0236757
PBK	-0,04815	0,0236592
KLRK1	-0,03349	0,0219191
CD97	-0,02671	0,0217524
OSM	-0,03046	0,0213065
USP39	-0,01793	0,0202705
CD1E	-0,05009	0,0198811
UBC	-0,04844	0,019227

PSEN2	0,01711	0,0178699
IL5RA	0,04796	0,0178555
CD80	-0,03905	0,0177233
C1QBP	0,02297	0,0174959
ITGA2	-0,034	0,0159221
CXCL6	-0,06131	0,0154704
STAT5B	0,01504	0,0152711
CD209	0,03615	0,0144792
S100A7	0,0276	0,0143293
CCR2	-0,02645	0,0134482
HLA-E	0,02811	0,0131687
CD6	-0,01989	0,0127316
IFNA7	-0,01932	0,012068
CD28	-0,03227	0,012022
BCL6	-0,01921	0,010649
IGF1R	-0,01292	0,0098481
CCR7	0,02644	0,0096172
IFNL1	-0,01273	0,0095923
CCR9	-0,01655	0,0090829
TANK	-0,005669	0,0083815
IFNA8	0,01772	0,0074611
DMBT1	-0,04124	0,0065378
LCP1	0,01054	0,0065272
ICAM4	0,009723	0,0057568
CLU	0,009631	0,0055166
BTLA	-0,01134	0,0053846
TGFB1	0,004314	0,0037638
IL34	0,00388	0,0030805
TOLLIP	0,003566	0,0028912
F2RL1	0,006315	0,0024088
SERPINB2	-0,004481	0,0023341
MBL2	-0,005863	0,0023315
CASP10	-0,002567	0,0010497
TLR6	-0,0000176	1,129E-05

Supplementary Table S6: Nanostring differential gene expression between pancreatic cancer patients SD vs PR: Multiple paired t-tests

Marker	log2 fold change	p-value (-log10)<br>post hoc BH
CLEC4A	1,104	2,67
ITGAX	1,345	1,977
IL22	2,408	1,954
FCF1	-0,8307	1,929
CSF2RB	1,023	1,808
EGR2	1,514	1,685
CXCR3	1,189	1,657
PRAME	1,081	1,573
AIRE	1,11	1,57
ENTPD1	1,315	1,57
HLA-DMB	0,7823	1,57
CSF3R	1,062	1,557
NCF4	0,7514	1,53
APP	-0,9312	1,521
IFIT1	-1,557	1,478
TREM2	1,709	1,429
KIR3DL2	1,345	1,409
CXCR4	0,9459	1,37
ABCF1	-0,5314	1,363
CXCL11	-1,793	1,347
CTLA4	1,109	1,339
TLR8	0,8327	1,338
IL18	0,7107	1,326
DOCK9	-0,5226	1,321
ATG16L1	-0,6549	1,316
PSMD7	-0,7125	1,308
SELE	1,313	1,29
FUT7	1,092	1,261
SPP1	2,831	1,242
ITK	1,063	1,236
BMI1	-0,6378	1,228
IKBKG	-0,7531	1,228

S100A12	1,559	1,223
CD37	0,6928	1,222
S100A8	1,615	1,217
REL	0,6457	1,212
IL23A	-1,187	1,2
C1S	-1,57	1,181
LAIR2	0,8916	1,181
ITGB1	-0,6592	1,175
CSF1R	0,7441	1,17
CLEC5A	1,69	1,165
SPN	0,8545	1,163
KIR_Inhibiting_Subgroup_2	1,299	1,15
CD1B	1,261	1,148
STAT2	-0,7704	1,147
HAVCR2	1,429	1,146
TNFSF13	0,7323	1,146
IFITM1	-1,442	1,142
TNFRSF10C	1,331	1,138
CD84	0,8655	1,135
ZC3H14	-0,3618	1,11
PSMB8	-0,8909	1,108
CLEC6A	0,7037	1,101
TXNIP	-1,121	1,098
CCL2	1,266	1,097
TLR10	0,6689	1,085
IL16	0,7189	1,076
DDX58	-0,9178	1,073
MFGE8	-1,035	1,064
CDH1	-2,438	0,996
FEZ1	-0,8765	0,9915
HLA-C	-0,7199	0,9743
ITGAM	1,013	0,9694
IFI27	-1,963	0,9635
NT5E	-1,051	0,9583
LY86	0,5388	0,9568
AMICA1	0,6853	0,9561
CASP8	-0,4799	0,9524
IL21	0,9367	0,9349
HLA-B	-0,9196	0,9334

CHIT1	2,212	0,9211
FPR2	1,273	0,918
MAP2K2	-0,4187	0,917
TNFRSF4	0,9625	0,9153
HCK	0,8419	0,9028
GZMM	0,6556	0,8939
SF3A3	-0,3597	0,8865
TNFRSF11B	1,177	0,8514
CARD9	0,8845	0,851
KLRG1	1,05	0,848
CLEC7A	0,7989	0,8442
BTK	0,5106	0,8363
IL18R1	0,6341	0,8334
CT45A1	0,9469	0,8257
IL17RA	0,665	0,8206
ITCH	-0,5864	0,8132
BTLA	1,023	0,8128
TNFRSF13B	1,393	0,81
LYN	0,4739	0,8081
C1R	-1,529	0,8014
ITGAL	0,3974	0,8008
CCRL2	0,6925	0,7997
MAPK1	-0,2652	0,7853
ISG15	-1,464	0,771
IL1R2	0,9158	0,7657
CFB	-1,067	0,7634
PSMB7	-0,5114	0,7619
CD79B	0,748	0,7455
SH2B2	0,6011	0,7435
CCL3L1	1,479	0,7364
JAK2	-0,4608	0,7364
TNFSF11	0,6335	0,731
CD46	-0,7318	0,7291
EOMES	0,6935	0,729
CCL3	1,3	0,7274
STAT3	-0,5646	0,7271
CCL18	2,232	0,7222
TAPBP	-0,6828	0,7212
CCL22	0,6834	0,7207

LILRB1	0,6057	0,7177
HAMP	1,448	0,7132
HLA-A	-0,8087	0,7114
LTBR	-0,4065	0,7104
TRAF3	-0,3717	0,7103
CXCR1	0,7741	0,6991
BIRC5	-0,9523	0,699
DMBT1	-2,777	0,6972
CCL20	1,079	0,6966
TFRC	1,143	0,6937
TLR1	0,408	0,6927
CD164	-0,6339	0,6925
IRAK1	-0,3727	0,6889
MX1	-0,9969	0,6853
GZMK	0,6964	0,6834
ITGA2B	1,373	0,6814
TAP1	-0,7317	0,6772
TNFSF14	0,4205	0,6739
IL17F	1,094	0,6719
IL13	0,5972	0,671
PRPF38A	-0,327	0,6706
GZMH	-0,6226	0,6671
MTMR14	0,4597	0,6667
IL15	0,6972	0,6561
IRAK4	-0,4246	0,6541
TLR2	0,5581	0,6505
AICDA	0,9684	0,6503
IRF7	-0,7269	0,6444
ITGA4	0,4987	0,6437
MAVS	-0,559	0,6406
ATF2	-0,2723	0,6364
ADA	0,3616	0,636
OAS3	-0,9762	0,6329
HLA-DMA	0,4282	0,6316
PRM1	0,7721	0,6299
IL1RN	1,001	0,6223
SLAMF1	0,8489	0,6207
CR2	0,8487	0,6198
CDK1	-0,8696	0,6191

ANP32B	-0,371	0,6184
LTA	0,584	0,6176
TLR4	0,3849	0,6175
ABCB1	1,412	0,6168
ICOSLG	0,3537	0,6142
EWSR1	-0,3543	0,6125
IL21R	0,9335	0,6076
HLA-DQB1	-0,6063	0,6062
IFI35	-0,4491	0,6044
CCR5	0,2678	0,6001
POLR2A	-0,3332	0,5987
PPBP	1,568	0,5944
HLA-G	-0,6438	0,5921
IL17A	0,7486	0,5911
IL13RA1	-0,3662	0,5906
SLC11A1	1,301	0,5835
CD1C	1,174	0,5801
CD86	0,6619	0,5789
BID	0,7657	0,5776
STAT4	0,5234	0,5756
VEGFA	-0,7875	0,5745
CD3E	0,5739	0,5725
CCL7	0,706	0,5713
ADORA2A	0,5468	0,5709
TNFRSF12A	-0,6642	0,5658
CCL4	1,006	0,5635
LILRA5	0,49	0,5628
ISG20	-0,4821	0,5601
ILF3	-0,3126	0,5545
PRKCD	0,4696	0,5539
CREB1	-0,2579	0,5533
CCR2	0,7454	0,5532
ITGA6	-0,5766	0,5516
CREBBP	0,4725	0,5464
CD40	0,4062	0,5422
TNFRSF8	0,7166	0,5416
IL7	0,6509	0,5414
CD19	0,6943	0,5392
CD22	0,8993	0,5378

CTSW	0,5541	0,5377
KLRD1	-0,6308	0,5359
EBI3	-0,5381	0,5353
TLR3	-0,6795	0,5344
CCR9	0,6673	0,5306
GAGE1	0,592	0,5276
C3	-1,768	0,527
MAP3K7	-0,3392	0,5248
MAGEA1	0,8715	0,523
APOE	1,108	0,5214
IL12RB1	0,328	0,5197
TNFSF18	-0,6991	0,5172
CD180	0,3663	0,5141
PIN1	-0,358	0,5123
SAP130	-0,3395	0,511
TNFRSF1A	-0,3753	0,5108
LIF	-0,7378	0,5078
TFE3	0,4935	0,5078
RUNX3	0,3861	0,5031
IL1B	0,9052	0,5029
GPATCH3	0,3171	0,5026
S100A7	-0,6817	0,5015
F2RL1	-0,912	0,4988
CD96	0,4385	0,4942
CAMP	0,8561	0,4937
MARCO	1,86	0,4907
IFNAR1	0,2831	0,4843
TNF	0,5359	0,4825
CSF1	-0,4479	0,482
STAT1	-0,8046	0,4801
IFNA17	0,5842	0,4777
ITGB4	-0,9817	0,4758
ELANE	0,5049	0,4727
SH2D1A	0,6031	0,4717
IFIT2	-0,6318	0,4684
IL6R	0,5049	0,4648
SLAMF6	0,4643	0,4646
KIR_Inhibiting_Subgroup_1	0,7244	0,4641
CD70	0,7257	0,464

PYCARD	0,4101	0,4639
TTK	-0,6158	0,4639
ERCC3	-0,2975	0,4608
HSD11B1	0,8696	0,4597
CRP	1,806	0,4584
RORA	0,5795	0,4582
SERPINB2	-0,6245	0,4548
EP300	-0,1661	0,4498
DPP4	0,7039	0,4493
YTHDF2	-0,21	0,4477
KIR3DL1	0,9415	0,4473
MAP3K1	0,2941	0,4464
CCR1	0,5874	0,4452
GATA3	0,668	0,4441
BCL2	0,6383	0,4439
IFNG	0,4656	0,4431
SH2D1B	0,6425	0,4385
CCL28	1,382	0,4369
CD1E	0,81	0,4358
IL6ST	-0,6369	0,4325
IL13RA2	0,502	0,4305
ATG12	-0,5432	0,4278
TICAM2	0,3523	0,4259
TNFSF10	-0,8248	0,4258
IL10	0,5255	0,4257
MS4A1	1,114	0,424
CCL15	1,017	0,4222
CTSH	0,514	0,4215
FADD	0,4062	0,4209
IL8	1,355	0,42
FAS	0,3907	0,4178
IFNA7	-0,4921	0,4148
TP53	-0,3497	0,4139
CD33	0,4666	0,4134
TNFSF13B	-0,3813	0,4126
ITGA5	-0,4857	0,4087
FYN	-0,294	0,4076
ITGB3	0,7963	0,4073
NEFL	0,7968	0,4067

PBK	-0,6206	0,4036
C1QA	-0,6452	0,4033
PIK3CD	0,2906	0,4001
CD160	0,5714	0,3997
CD1D	0,5067	0,3994
CD27	0,4919	0,3994
C9	1,144	0,3987
HMGB1	-0,2232	0,3978
BAGE	0,4404	0,3976
NUBP1	0,3302	0,3966
EPCAM	-1,288	0,3962
POU2AF1	0,5724	0,3939
IL7R	0,4237	0,3882
TGFB1	0,3319	0,388
ATM	0,7337	0,3872
MAPK14	-0,2076	0,3871
CD247	0,3504	0,3843
AKT3	0,3575	0,3839
ITGA1	-0,5183	0,383
IL1RL1	0,4312	0,3821
NLRP3	0,342	0,3808
PPARG	0,3344	0,3803
PVR	-0,2838	0,3794
BLNK	0,4964	0,3789
G6PD	0,3741	0,3783
NOS2A	0,8347	0,378
TLR9	0,2676	0,3777
PSMB9	-0,5151	0,3754
GTF3C1	-0,2295	0,3748
SELL	0,6552	0,3731
SDHA	-0,3379	0,3731
KLRB1	0,5759	0,3723
IDO1	-0,4669	0,3719
CD53	0,4418	0,3713
SOCS1	0,5948	0,3702
PASD1	0,4049	0,3695
MAGEA12	0,5701	0,3691
IFI16	-0,4772	0,3681
CXCL9	-0,6934	0,3672

C3AR1	0,4323	0,3671
SPACA3	0,446	0,367
CCR3	-0,7215	0,3664
ALAS1	0,3555	0,3591
MASP1	0,4349	0,3587
EIF2B4	-0,2949	0,3583
LRP1	-0,6183	0,3553
PLAUR	0,7531	0,3551
CR1	0,4765	0,3546
C8B	0,8676	0,3534
HLA-DRB3	0,4556	0,3529
CD276	0,2268	0,3527
CEACAM1	-0,4008	0,3522
CD59	-0,355	0,3496
EDC3	-0,3061	0,347
GZMA	-0,3717	0,3462
IL12B	0,6426	0,3461
IL12A	-0,3353	0,3452
CD44	0,4195	0,3449
NOTCH1	0,3715	0,3446
CD48	0,4024	0,3443
IFNGR1	-0,3015	0,3435
IL17B	0,6179	0,3434
HLA-DOB	0,3013	0,3433
IFITM2	-0,3681	0,3427
ARG2	0,5707	0,3407
CCL11	0,6196	0,34
CD274	0,5775	0,3397
CCL16	0,8063	0,3396
CD209	0,6584	0,338
NFATC2	-0,1652	0,3367
TREM1	0,9891	0,3364
CD3D	0,3432	0,336
CXCR2	0,5099	0,3359
IRF1	-0,2305	0,3358
SYK	0,2734	0,3353
IL2	0,5632	0,3347
CD38	-0,6995	0,331
CCL23	0,4296	0,3309

VEGFC	-0,4357	0,3309
CTAG1B	0,4368	0,3291
PDCD1	0,4611	0,3291
COG7	-0,2766	0,3283
TIRAP	0,1668	0,3282
PNMA1	-0,2476	0,3263
MST1R	-0,9659	0,3246
FCGR2B	0,551	0,3236
ICAM3	0,2976	0,3235
LY9	-0,3632	0,3208
THBS1	-0,5907	0,3207
FCER2	0,4053	0,3196
COL3A1	0,5604	0,3194
IL19	0,5978	0,3164
TUBB	-0,3719	0,3162
IRGM	0,3534	0,3143
IRAK2	0,3512	0,3131
IFIH1	-0,3897	0,3127
CXCR6	0,4747	0,3123
IL11	0,9401	0,3114
TARP	0,2943	0,3098
CTSG	0,6559	0,3083
CD1A	0,3894	0,3078
CX3CL1	-0,4072	0,3024
CD68	0,5596	0,3015
CD47	-0,3927	0,3009
HLA-E	-0,5125	0,3004
MAPK3	-0,276	0,299
TRIM39	0,2176	0,299
IL10RA	0,2368	0,2984
BST2	-0,4214	0,2977
CD5	0,2945	0,296
CYFIP2	0,5202	0,2941
CD83	0,4192	0,294
CNOT10	-0,2058	0,2933
TNFRSF9	0,5089	0,2927
RELB	0,3635	0,2923
FCER1A	0,7915	0,2922
PLAU	0,4063	0,2908

NOD1	0,2411	0,2897
MS4A2	-0,5544	0,2868
CXCL10	-0,8485	0,2863
MAP3K5	0,1556	0,285
LTK	0,486	0,2848
HPRT1	-0,3376	0,2838
BATF	-0,2758	0,2799
CCL19	1,13	0,2799
TMUB2	-0,269	0,2793
F13A1	-0,8586	0,2789
ARG1	1,119	0,275
IGF2R	0,2587	0,2746
UBC	-0,5654	0,2737
C1QB	-0,6368	0,2717
TANK	-0,149	0,2716
IFNL2	0,3894	0,2712
SYT17	0,3616	0,2689
CCR7	0,6016	0,2688
IL12RB2	-0,6268	0,2688
PAX5	0,3734	0,2688
IRF5	0,3504	0,268
TNFRSF18	0,4866	0,2679
SIGLEC1	-0,4145	0,2665
MYD88	-0,2774	0,2651
LAMP1	-0,2255	0,2649
TAL1	0,5355	0,2649
SMAD2	0,2098	0,2627
PTGS2	0,704	0,2619
TBK1	-0,1446	0,2611
SPA17	-0,4239	0,2606
CNOT4	-0,2553	0,2604
PIK3CG	0,2515	0,2597
ZNF346	0,2976	0,2561
CD40LG	0,2547	0,2554
TLR5	0,3739	0,254
AXL	-0,2854	0,2536
IL27	0,4368	0,25
TICAM1	0,1813	0,25
CD244	0,2597	0,2498

CD3G	0,2513	0,2495
TPSAB1	0,4905	0,2479
PLA2G1B	-1,178	0,247
HLA-DRB4	0,4702	0,2467
LILRB2	0,2254	0,2467
CCND3	-0,1424	0,2448
DEFB1	0,6895	0,2446
IL17RB	0,5884	0,2446
ZNF143	0,2583	0,243
ROPN1	0,3087	0,2418
FLT3	0,3972	0,2407
IL1RAPL2	-0,2504	0,2381
TBX21	0,2434	0,2379
CEACAM6	-1,193	0,2367
FCER1G	0,3877	0,2365
BLK	0,3909	0,235
PSMB10	-0,1912	0,234
CLU	-0,3392	0,2337
PTPRC	0,1982	0,2336
IFNAR2	0,1493	0,2334
IL1RAP	-0,2928	0,2334
SIGIRR	-0,315	0,2326
TNFAIP3	0,2213	0,2322
TNFSF8	0,3042	0,232
RIPK2	0,1603	0,2313
TFEB	0,2501	0,2308
SLAMF7	0,3203	0,2303
EGR1	-0,2646	0,2291
CCL5	-0,2254	0,229
IL9	0,3671	0,2286
IRF8	0,2247	0,2262
CCL25	0,2792	0,2224
MAP2K4	-0,1607	0,2196
IL24	0,3254	0,219
SERPING1	0,4675	0,2184
MAP2K1	0,1389	0,2163
SMAD3	-0,1741	0,2161
CD6	-0,2862	0,2154
CSF2	0,4124	0,2138

CD24	-0,9088	0,2127
MNX1	0,5684	0,2123
CD4	0,2479	0,2114
KIR_Activating_Subgroup_1	0,3924	0,2099
AMBP	1,174	0,2093
CCL21	-0,7279	0,2087
ST6GAL1	-0,3309	0,2085
IFNA1	0,3732	0,2078
TNFRSF14	0,1901	0,2075
TAP2	-0,2059	0,2074
C4B	-0,5274	0,207
TNFRSF11A	0,378	0,2061
PRG2	0,3097	0,2053
NFATC1	0,1533	0,2046
MAP4K2	0,2354	0,2013
IRF4	0,354	0,2003
TIGIT	0,2117	0,2001
NOL7	-0,1495	0,2001
CCL8	0,4895	0,1992
LCK	0,1645	0,1982
IL1RL2	0,3043	0,1972
CSF3	0,489	0,196
MRC1	0,3708	0,196
ATF1	0,1229	0,1957
TRAF2	-0,1958	0,1955
TMEFF2	0,2626	0,1951
CD163	-0,4375	0,1927
CFD	-0,4657	0,1921
ITGA2	-0,3561	0,1919
ICAM2	0,1536	0,1918
CD97	0,2051	0,1908
ICAM1	0,2535	0,1884
STAT5B	0,1612	0,1879
NUP107	-0,1505	0,1873
LAMP3	-0,3585	0,1869
RPS6	-0,2107	0,1851
CXCL5	-0,6537	0,185
SPO11	0,3854	0,1844
FCGR2A	0,2726	0,1842

BCL10	-0,1542	0,184
IL26	0,2247	0,1835
SEMG1	0,2281	0,1828
VCAM1	-0,3489	0,1821
PRKCE	0,3176	0,1811
IFNA8	-0,3721	0,1803
IFNL1	-0,2084	0,1803
CCR6	0,2445	0,1798
IL11RA	-0,2737	0,1783
KLRK1	-0,2403	0,1777
CREB5	0,2658	0,1773
JAK1	-0,1506	0,1771
CD81	-0,2174	0,1768
IRF2	0,175	0,1739
LGALS3	0,4238	0,1737
REPS1	-0,1459	0,1736
IL32	-0,3162	0,1726
HDAC3	0,112	0,1725
TNFSF15	0,3897	0,1721
CCL24	0,4929	0,1719
TLK2	-0,1166	0,1716
LAMP2	0,1784	0,1709
F12	0,3399	0,1698
C1QBP	-0,1972	0,1693
IL22RA1	-0,3188	0,1688
CTSL	0,3359	0,1684
LCP1	0,2375	0,1676
NCAM1	-0,3077	0,1668
CD99	-0,1961	0,1655
DHX16	-0,1944	0,1655
USP39	-0,1306	0,1654
SPINK5	0,2669	0,1633
MASP2	0,341	0,163
CCL26	-0,4123	0,1627
CCL14	0,503	0,1626
IL6	0,2978	0,1599
TNFSF4	0,2082	0,159
ETS1	-0,1843	0,1584
ALCAM	0,2256	0,1583

ATG5	0,1052	0,1571
MICB	-0,1904	0,1565
CDH5	-0,2157	0,1563
ABL1	-0,1092	0,1544
CD7	0,1567	0,1544
SMPD3	0,3417	0,1537
IL4	0,2647	0,1525
CXCL12	-0,4057	0,1517
C6	0,7004	0,1513
RORC	0,3077	0,1498
IL2RA	0,2702	0,1497
CXCL13	0,3888	0,1495
CXCL1	0,3348	0,1481
FLT3LG	0,1937	0,1481
DDX43	-0,3427	0,1467
GPI	-0,1476	0,1431
TAB1	-0,1549	0,1421
DUSP4	0,2318	0,142
IKBKB	-0,155	0,1412
CKLF	-0,1085	0,1401
LILRB3	0,2528	0,1398
ANXA1	-0,3055	0,1394
CD2	0,1889	0,1379
BAX	0,1111	0,1375
FCGR1A	0,2023	0,136
TNFSF12	0,2659	0,136
MAGEC1	-0,1972	0,1356
IL18RAP	0,2209	0,1343
RRAD	-0,4288	0,1335
IGLL1	0,2153	0,1321
INPP5D	0,1391	0,1311
MBL2	-0,2942	0,1297
IKBKE	0,1411	0,1295
CD28	0,3133	0,1278
AGK	-0,1313	0,1278
HLA-DRA	0,237	0,1267
CD34	0,2559	0,1265
KIT	0,3015	0,1255
CXCL6	0,4549	0,1249

PRF1	-0,1347	0,1234
ZAP70	0,126	0,1216
CD14	0,2478	0,1204
IL1A	-0,2637	0,1188
CX3CR1	-0,273	0,1186
BCL2L1	-0,09756	0,1164
GUSB	0,09398	0,1164
AMMECR1L	0,1082	0,1163
MEF2C	-0,1268	0,1146
XCR1	0,1678	0,1131
LRRN3	0,2279	0,113
KLRC2	-0,1992	0,1113
CMKLR1	0,1222	0,111
SSX1	0,2269	0,11
CXCR5	0,2138	0,1096
NOD2	0,204	0,1091
TNFRSF10B	0,09856	0,109
JAM3	-0,2242	0,1071
MME	0,3239	0,1057
JAK3	-0,1245	0,1048
TLR6	0,15	0,1044
KIR3DL3	0,1677	0,1037
TYK2	-0,09496	0,1035
LTB	0,2161	0,1031
MSR1	0,1906	0,1024
IL2RB	-0,0976	0,1012
HLA-DPB1	0,254	0,1009
MAGEB2	-0,192	0,1007
HLA-DPA1	0,1705	0,1006
USP9Y	0,1995	0,1002
IL25	0,1792	0,1001
CD63	0,1374	0,09711
CCL17	0,1703	0,09487
NFKB2	0,07834	0,09484
IL22RA2	0,1063	0,0946
CHUK	-0,07179	0,09452
CASP10	-0,2108	0,09253
SAA1	-0,386	0,09201
MRPS5	0,06701	0,092

MPPED1	0,166	0,09013
CLEC4C	0,214	0,08971
COLEC12	-0,2742	0,08924
THY1	0,1967	0,0892
MICA	0,1296	0,0887
ZKSCAN5	-0,1119	0,08769
LAG3	-0,2017	0,0876
IL23R	0,1527	0,08659
CFP	0,1449	0,08569
TLR7	-0,1511	0,08408
ATG10	-0,1358	0,08392
XCL2	0,1018	0,08391
MIF	-0,09872	0,08221
MAPKAPK2	-0,05524	0,08165
LILRA1	0,0978	0,08052
TCF7	-0,1044	0,08048
IRF3	-0,09134	0,07996
FN1	-0,234	0,07939
CCL27	-0,1244	0,07933
CD8A	0,1218	0,07805
CD8B	-0,1342	0,07804
CCR4	0,1594	0,07774
CD36	-0,265	0,07719
C4BPA	0,3377	0,07645
IL3RA	0,1685	0,07579
ATG7	0,0507	0,07438
ICAM4	0,1197	0,07435
RAG1	0,124	0,07387
CTAGE1	-0,09564	0,07195
KIR_Activating_Subgroup_2	-0,1535	0,07178
MCAM	0,1562	0,07122
SPANXB1	0,1248	0,07067
CTCFL	-0,1297	0,06946
TXK	-0,1465	0,06934
CASP1	0,06254	0,0689
CD55	-0,125	0,06886
ICOS	0,12	0,06881
GZMB	-0,1683	0,06756
DUSP6	0,08938	0,06717

PSEN1	0,04179	0,06707
NFATC4	0,1169	0,06576
CD58	-0,0625	0,06434
TBP	0,06782	0,0631
CXCL16	0,09251	0,0629
SELPLG	-0,07236	0,06232
TNFRSF1B	-0,06299	0,06207
PSEN2	0,05797	0,06188
HRAS	-0,07618	0,06174
MR1	-0,08266	0,06169
ENG	0,1256	0,06016
PECAM1	-0,0879	0,06007
IFNB1	0,1014	0,05988
ITGAE	0,07121	0,05863
CXCL2	-0,1325	0,05854
A2M	-0,1521	0,05789
CMA1	0,1555	0,05761
NFKBIA	0,07512	0,0572
CXCL3	-0,1598	0,05692
IL5RA	0,1497	0,05668
NFKB1	0,07195	0,05634
BST1	-0,104	0,05588
FCGR3A	0,1404	0,05536
C2	0,1171	0,05488
IL5	-0,1277	0,05483
IL34	0,06654	0,0545
FOXJ1	-0,1716	0,05427
NRP1	-0,09187	0,05409
C5	0,1153	0,05353
IL4R	-0,1025	0,05345
IL1R1	-0,08721	0,05269
BCL6	-0,09309	0,05267
C7	0,2505	0,05239
PDGFC	0,07127	0,05232
CTSS	0,1347	0,05229
TRAF6	0,03793	0,05197
MAGEA4	0,1197	0,05114
PMCH	-0,08747	0,05062
DDX50	0,0496	0,05009

ELK1	0,04306	0,04999
STAT6	-0,02722	0,04971
NCR1	0,07373	0,04765
MUC1	-0,1708	0,04655
PDCD1LG2	0,08337	0,04655
CEACAM8	0,05944	0,04653
MAF	-0,05594	0,04447
CXCL14	-0,1574	0,04319
ITGB2	0,08617	0,04264
ULBP2	0,08771	0,04187
KLRF1	-0,09474	0,04051
CCL1	0,07037	0,0404
IGF1R	-0,0523	0,04023
CD207	0,07223	0,03837
LILRA4	-0,06753	0,03726
MAGEC2	-0,04418	0,03722
GNLY	-0,08337	0,0371
CFI	-0,09573	0,03624
S100B	-0,08933	0,03621
SBNO2	-0,03544	0,03536
MAPK11	0,07235	0,03388
CD3EAP	0,03144	0,0336
FOXP3	0,0625	0,03343
TPTE	-0,03723	0,03311
KLRC1	-0,04252	0,03251
ZNF205	-0,04965	0,03197
C8G	0,07573	0,03096
FUT5	0,05673	0,0306
FOS	-0,05143	0,03002
THBD	0,0442	0,02897
CDKN1A	0,03066	0,02849
PTGDR2	0,08566	0,02813
CD200	-0,05215	0,02709
ECSIT	-0,03354	0,02597
HLA-DQA1	0,07708	0,0253
NFATC3	-0,01956	0,02451
CASP3	-0,02179	0,02447
DNAJC14	0,02723	0,02444
LY96	0,02192	0,02274

IFNA2	-0,05278	0,02224
CARD11	0,02152	0,02007
CD79A	-0,0404	0,01988
RUNX1	-0,02311	0,01854
TOLLIP	-0,02118	0,01703
TGFB2	0,03953	0,01512
CD74	0,03248	0,01511
NLRC5	0,02237	0,0146
C8A	0,04158	0,01262
CYLD	-0,01021	0,01198
PPIA	0,01013	0,01172
MERTK	0,01738	0,01165
CD9	0,01273	0,01078
SSX4	0,02564	0,01005
TNFRSF17	0,02391	0,009816
MAGEA3	0,01669	0,009807
LTF	-0,04346	0,009682
LBP	0,03722	0,009578
POU2F2	-0,009397	0,008956
CC2D1B	0,01674	0,008855
IL15RA	0,007148	0,008569
PLA2G6	-0,01255	0,008088
CYBB	0,01778	0,007911
MAPK8	-0,01011	0,007689
LCN2	-0,03731	0,007568
PDGFRB	0,01373	0,006082
CD80	0,01327	0,0058
TNFRSF13C	0,0149	0,005712
OSM	-0,00828	0,005554
CCL13	-0,01433	0,004533
IL2RG	0,008768	0,00399
IL3	0,004225	0,002349
RELA	-0,001524	0,002173
MEFV	0,00494	0,001958
CEBPB	-0,002815	0,001061
SYCP1	-0,001267	0,0008752

Supplementary Table S7: Olink in plasma differential expression between pancreatic cancer patients response and survival

**PD**	P value	Mean of w7	Mean of w1	Difference	SE of difference	-log10(P value)
MIC-A/B	0,001563	4,627	4,157	0,4702	0,08602	2,806
IL6	0,002345	4,32	2,637	1,683	0,3337	2,63
IL12RB1	0,002636	1,786	0,8966	0,8892	0,1805	2,579
ANGPT2	0,003932	4,883	4,286	0,5967	0,1314	2,405
CD5	0,007078	4,7	4,188	0,5124	0,1279	2,15
CCL23	0,008978	10,08	9,636	0,4475	0,1178	2,047
HO-1	0,009064	12,47	11,85	0,6201	0,1636	2,043
CAIX	0,00966	5,478	4,897	0,5804	0,1553	2,015
Gal-9	0,010468	7,381	6,926	0,4542	0,1238	1,98
CSF-1	0,010683	9,06	8,724	0,3364	0,09212	1,971
MCP-3	0,011129	3,539	2,783	0,756	0,209	1,954
ADGRG1	0,01121	1,411	0,8215	0,5896	0,1632	1,95
CD4	0,011889	2,975	2,578	0,3967	0,1113	1,925
CX3CL1	0,012026	3,148	2,666	0,4817	0,1356	1,92
TNF	0,013261	3,816	2,99	0,8254	0,2377	1,877
TIE2	0,013478	6,205	5,872	0,3329	0,09623	1,87
IL15	0,013698	3,515	3,034	0,4807	0,1395	1,863
IL18	0,015591	9,242	8,252	0,9904	0,2964	1,807
PD-L2	0,018286	1,302	0,8432	0,4585	0,1427	1,738
VEGFR-2	0,019379	6,641	6,495	0,1463	0,04618	1,713
LAG3	0,020075	3,029	2,415	0,6137	0,1955	1,697
KLRD1	0,021233	6,117	5,401	0,7162	0,2313	1,673
CXCL11	0,024117	10,76	9,737	1,019	0,3402	1,618
PGF	0,027491	7,845	7,462	0,3822	0,132	1,561
CCL19	0,028519	9,726	9,164	0,5623	0,1961	1,545
MCP-1	0,028648	11,05	10,44	0,6109	0,2133	1,543
TNFRSF4 (OX40R)	0,029638	5,363	4,829	0,5347	0,1884	1,528
PDCD1 (PD-1)	0,030719	6,172	5,499	0,6731	0,2395	1,513
TNFRSF12A (TWEAKR)	0,031076	4,916	4,553	0,3629	0,1295	1,508
IL8	0,034103	6,856	6,286	0,5699	0,2086	1,467

CCL3	0,034616	5,97	5,425	0,5448	0,2002	1,461
CXCL9	0,035337	7,265	5,972	1,293	0,4779	1,452
LAMP3	0,039642	4,919	3,87	1,049	0,4006	1,402
CXCL10	0,040047	8,802	7,347	1,455	0,5573	1,397
EGF	0,041535	7,941	8,678	-0,7373	0,2853	1,382
CD8A	0,041867	9,285	8,712	0,5734	0,2224	1,378
VEGFA	0,053423	8,959	8,665	0,2943	0,1227	1,272
CXCL13	0,055956	7,345	6,763	0,5822	0,2462	1,252
CRTAM	0,058443	4,768	4,154	0,6138	0,2632	1,233
GZMA	0,058539	5,794	5,244	0,5499	0,2359	1,233
CCL17	0,061564	7,837	8,608	-0,7707	0,3359	1,211
IFNg	0,062958	7,878	6,019	1,859	0,816	1,201
MUC-16	0,078638	5,506	4,821	0,6845	0,3234	1,104
TNFRSF21 (Death receptor 6)	0,088947	7,659	7,479	0,1795	0,08851	1,051
CD70	0,090692	2,962	2,577	0,3855	0,1914	1,042
CXCL15	0,092605	10,05	10,54	-0,4946	0,2475	1,033
4-1BB	0,105508	4,592	4,158	0,4341	0,2279	0,9767
TRAIL	0,107198	7,712	7,531	0,1808	0,0955	0,9698
CD27	0,110606	7,459	7,115	0,3439	0,1839	0,9562
CD40L	0,127027	5,697	6,33	-0,6329	0,3575	0,8961
ANGPT1	0,131651	8,281	8,566	-0,2853	0,1635	0,8806
NOS3	0,133253	0,8463	0,3657	0,4806	0,2768	0,8753
TWEAK	0,138554	7,671	7,844	-0,1723	0,1009	0,8584
CD83	0,201349	1,718	1,511	0,2068	0,1441	0,696
HGF	0,215554	7,554	7,345	0,2084	0,1505	0,6664
CASP-8	0,224682	4,495	4,826	-0,3309	0,2445	0,6484
PDGF subunit B	0,230967	9,268	9,475	-0,2066	0,155	0,6364
DCN (Decorin)	0,287924	1,727	1,669	0,05816	0,04988	0,5407
GZMB	0,308459	2,945	2,372	0,5727	0,5148	0,5108
NCR1	0,326792	2,007	1,867	0,1392	0,1304	0,4857
GZMH	0,329028	5,404	4,529	0,8748	0,8236	0,4828
MCP-2	0,336295	7,679	7,491	0,1886	0,1805	0,4733
IL10	0,339204	2,772	2,424	0,3482	0,3354	0,4695
FASLG	0,34845	4,966	4,84	0,1263	0,1242	0,4579

MMP12	0,354134	6,746	6,947	-0,2015	0,2007	0,4508
ADA	0,428378	4,147	3,968	0,1791	0,211	0,3682
CCL20	0,448368	8,312	8	0,3127	0,3856	0,3484
MMP7	0,458533	11,99	11,95	0,03551	0,04484	0,3386
MCP-4	0,517502	11,66	11,79	-0,1355	0,1972	0,2861
PD-L1	0,533508	5,625	5,468	0,157	0,2378	0,2729
LAP TGF-beta-1	0,553843	8,285	8,192	0,09248	0,1475	0,2566
CD244	0,559004	4,858	4,98	-0,1216	0,1966	0,2526
IL12	0,621295	4,34	4,53	-0,1906	0,3662	0,2067
CD40	0,667798	10,25	10,34	-0,08962	0,1987	0,1754
CCL4	0,693758	6,426	6,361	0,06552	0,1585	0,1588
IL7	0,74331	1,967	2,008	-0,04135	0,1206	0,1288
CXCL1	0,901862	8,53	8,553	-0,02243	0,1744	0,04486
Gal-1	0,911663	5,508	5,523	-0,01519	0,1313	0,04017
ICOSLG	0,944944	3,655	3,65	0,00513	0,07125	0,02459
TNFSF14 (LIGHT)	0,97679	4,6	4,595	0,005687	0,1875	0,0102

**SD**	P value	Mean of w7	Mean of w1	Difference	SE of difference	-log10(P value)
CX3CL1	0,000121	3,323	2,682	0,6411	0,1108	3,916
IL15	0,000141	3,729	2,962	0,7677	0,135	3,85
IL18	0,000417	9,089	8,369	0,72	0,1446	3,38
MMP7	0,000436	11,77	11,87	-0,09555	0,01931	3,36
HO-1	0,000476	12,26	11,65	0,6055	0,1237	3,323
Gal-9	0,000577	7,405	6,883	0,5223	0,1094	3,239
CSF-1	0,000763	9,017	8,792	0,2245	0,04879	3,118
LAG3	0,000987	3,034	2,582	0,4518	0,1017	3,005
PGF	0,001102	8,057	7,638	0,4187	0,09563	2,958
ANGPT2	0,00132	5,143	4,689	0,4541	0,1063	2,88
IL12	0,001629	4,034	5,499	-1,464	0,3532	2,788
TIE2	0,001914	6,267	6,013	0,2539	0,06269	2,718
PDGF subunit B	0,002108	8,72	9,221	-0,5018	0,1256	2,676
PD-L2	0,004837	1,401	1,076	0,3244	0,09227	2,315
TNFRSF4 (OX40R)	0,005514	5,905	5,455	0,45	0,1308	2,259
IL12RB1	0,006029	1,265	0,961	0,3044	0,08979	2,22

TNFRSF21 (Death receptor 6)	0,007585	7,766	7,626	0,1401	0,04297	2,12
CXCL15	0,010773	9,254	10,01	-0,7585	0,2475	1,968
CCL17	0,011214	7,897	8,917	-1,02	0,3353	1,95
CD4	0,013447	3,007	2,75	0,2574	0,08756	1,871
CCL23	0,015469	9,827	9,569	0,2576	0,09003	1,811
CXCL13	0,015897	7,239	6,939	0,3006	0,1056	1,799
CRTAM	0,016966	4,811	4,542	0,2686	0,09559	1,77
MCP-1	0,018873	11,29	10,79	0,5025	0,1827	1,724
PD-L1	0,022069	5,462	5,168	0,294	0,1104	1,656
ANGPT1	0,024712	7,515	7,97	-0,4549	0,175	1,607
IFNg	0,028886	7,768	6,695	1,073	0,4271	1,539
TNF	0,049557	3,707	3,259	0,4478	0,203	1,305
KLRD1	0,051644	5,599	5,303	0,2966	0,1359	1,287
CXCL11	0,052663	10,45	10,16	0,2907	0,1339	1,278
LAMP3	0,053089	4,568	4,194	0,3745	0,1728	1,275
MIC-A/B	0,053588	4,332	4,153	0,1785	0,0826	1,271
MMP12	0,055754	6,502	6,771	-0,2685	0,1255	1,254
CXCL10	0,056123	8,366	7,876	0,4893	0,2292	1,251
CD5	0,058821	4,741	4,562	0,1789	0,0849	1,23
ADGRG1	0,063044	0,9011	0,688	0,2132	0,1031	1,2
VEGFR-2	0,063349	6,47	6,351	0,1191	0,05769	1,198
ADA	0,06367	4,01	3,697	0,3123	0,1515	1,196
VEGFA	0,068355	8,947	8,707	0,2397	0,1186	1,165
MCP-3	0,091313	2,997	2,712	0,2851	0,1541	1,039
CD70	0,106245	3,144	2,931	0,2129	0,121	0,9737
CD8A	0,108857	9,27	9,028	0,2423	0,1389	0,9631
EGF	0,150812	7,335	7,745	-0,4092	0,265	0,8216
CCL3	0,163112	5,793	5,463	0,3298	0,2207	0,7875
4-1BB	0,164536	4,822	4,644	0,1784	0,1198	0,7837
ICOSLG	0,205002	3,8	3,722	0,07846	0,05824	0,6882
FASLG	0,20553	4,832	5,042	-0,2103	0,1563	0,6871
MCP-2	0,254563	7,684	7,511	0,1735	0,1443	0,5942
CCL20	0,255653	8,5	8,073	0,4268	0,3559	0,5923
CD27	0,259772	7,562	7,463	0,0985	0,0829	0,5854

CXCL9	0,271324	6,58	6,371	0,2088	0,1803	0,5665
MCP-4	0,282853	11,99	11,84	0,1516	0,1342	0,5484
GZMA	0,302273	5,405	5,207	0,1981	0,1831	0,5196
MUC-16	0,324389	3,357	3,1	0,2567	0,2488	0,4889
HGF	0,332193	7,334	7,23	0,1041	0,1026	0,4786
CAIX	0,334436	4,612	4,376	0,2355	0,2333	0,4757
TRAIL	0,344975	7,522	7,392	0,1303	0,1321	0,4622
CD83	0,346316	1,758	1,898	-0,1396	0,1419	0,4605
CD40L	0,351317	5,173	5,508	-0,3345	0,3436	0,4543
TNFSF14 (LIGHT)	0,391598	3,796	4,005	-0,2099	0,2353	0,4072
Gal-1	0,39299	5,426	5,359	0,06696	0,07531	0,4056
NCR1	0,441467	2,079	2,194	-0,1149	0,1438	0,3551
NOS3	0,444388	0,1897	0,3095	-0,1198	0,151	0,3522
PDCD1 (PD-1)	0,474377	5,911	5,825	0,08603	0,1161	0,3239
DCN (Decorin)	0,475364	1,682	1,639	0,04364	0,05905	0,323
LAP TGF-beta-1	0,508325	7,979	7,865	0,1143	0,1673	0,2939
TNFRSF12A (TWEAKR)	0,531016	4,828	4,748	0,0797	0,1232	0,2749
CCL19	0,549213	9,967	10,07	-0,1078	0,1745	0,2603
GZMB	0,549273	2,746	2,592	0,1546	0,2503	0,2602
IL10	0,552793	2,412	2,343	0,06927	0,1131	0,2574
CXCL1	0,64018	7,948	7,887	0,06096	0,1268	0,1937
CCL4	0,668778	6,441	6,351	0,09023	0,2053	0,1747
CASP-8	0,713057	4,12	4,04	0,08032	0,2128	0,1469
TWEAK	0,718414	7,741	7,684	0,05654	0,1528	0,1436
IL7	0,754314	1,399	1,466	-0,06714	0,2092	0,1224
IL6	0,8158	3,913	3,966	-0,0526	0,2204	0,08842
CD40	0,816802	10,01	9,978	0,02896	0,122	0,08788
IL8	0,834731	5,632	5,606	0,02608	0,1221	0,07845
GZMH	0,94417	4,317	4,294	0,02285	0,3189	0,02495
CD244	0,975712	4,783	4,788	-0,004727	0,1518	0,01068

**PR**	P value	Mean of w7	Mean of w1	Difference	SE of difference	-log10(P value)
Gal-9	0,000476	7,598	7,137	0,461	0,05721	3,322
CX3CL1	0,000913	3,156	2,566	0,5906	0,08429	3,04

CXCL15	0,001177	8,943	10,53	-1,582	0,2387	2,929
IL12	0,001597	3,999	6,042	-2,043	0,3297	2,797
IL18	0,001891	9,576	8,839	0,7366	0,1234	2,723
ANGPT1	0,002069	7,148	8,369	-1,222	0,2089	2,684
CCL17	0,002799	6,95	8,782	-1,832	0,3355	2,553
PDGF subunit B	0,002871	8,694	9,432	-0,7385	0,136	2,542
LAG3	0,003051	3,302	2,904	0,3981	0,07434	2,516
HO-1	0,003908	12,42	11,48	0,9391	0,1857	2,408
EGF	0,004384	6,904	8,374	-1,471	0,2988	2,358
CD40L	0,004558	4,662	6,178	-1,517	0,3108	2,341
CD83	0,007337	1,451	1,87	-0,4188	0,0962	2,134
MMP12	0,022933	5,968	6,9	-0,9319	0,2876	1,64
CXCL10	0,025971	9,018	8,314	0,7046	0,2251	1,586
CSF-1	0,026568	9,086	8,88	0,2061	0,06629	1,576
IL15	0,02787	3,338	2,757	0,5808	0,1893	1,555
MMP7	0,027952	11,47	11,81	-0,3387	0,1105	1,554
IL7	0,029128	1,306	2,11	-0,8039	0,2654	1,536
CD244	0,03302	4,493	5,048	-0,5556	0,1903	1,481
LAP TGF-beta-1	0,034118	7,583	8,212	-0,6292	0,2176	1,467
TNFRSF4 (OX40R)	0,041093	5,548	5,215	0,3331	0,1219	1,386
IFNg	0,041993	8,991	6,65	2,341	0,862	1,377
CASP-8	0,044768	3,894	4,651	-0,7569	0,2843	1,349
TNFSF14 (LIGHT)	0,044899	3,535	4,363	-0,8278	0,3112	1,348
CXCL9	0,059748	6,997	6,58	0,417	0,172	1,224
MCP-1	0,061052	11,31	10,65	0,662	0,275	1,214
PGF	0,069035	7,973	7,639	0,3339	0,1447	1,161
CD40	0,071898	10,11	10,43	-0,3246	0,1426	1,143
CD8A	0,085163	9,36	8,891	0,469	0,219	1,07
TWEAK	0,096221	7,272	7,75	-0,4783	0,2338	1,017
CCL20	0,111759	7,597	8,114	-0,5167	0,268	0,9517
NCR1	0,11582	1,955	2,261	-0,3059	0,161	0,9362
MUC-16	0,116325	3,578	4,418	-0,8401	0,4429	0,9343
TNF	0,125412	3,526	3,314	0,2122	0,1154	0,9017
VEGFR-2	0,126521	6,555	6,762	-0,2062	0,1126	0,8978

CXCL11	0,150475	10,52	10,06	0,4592	0,2706	0,8225
LAMP3	0,162688	4,752	4,477	0,2757	0,1685	0,7886
KLRD1	0,164843	6,045	5,752	0,2931	0,1803	0,7829
FASLG	0,170436	4,749	5,214	-0,4646	0,2903	0,7684
CRTAM	0,178394	4,718	4,487	0,2303	0,1472	0,7486
CD4	0,196348	3,054	2,918	0,1358	0,09115	0,707
CXCL1	0,205538	8,102	8,447	-0,3457	0,2377	0,6871
TNFRSF21 (Death receptor 6)	0,206808	7,648	7,507	0,1417	0,09771	0,6844
CD70	0,245146	2,791	2,988	-0,1969	0,1496	0,6106
VEGFA	0,254222	8,783	9,03	-0,2468	0,1916	0,5948
CCL23	0,278999	10,21	10	0,2033	0,1675	0,5544
GZMB	0,282248	3,022	2,646	0,377	0,3129	0,5494
GZMH	0,306875	5,18	4,378	0,8016	0,7047	0,513
IL12RB1	0,312752	1,435	1,228	0,2072	0,1846	0,5048
PD-L2	0,359556	1,124	0,9951	0,1288	0,1278	0,4442
NOS3	0,371357	0,7144	0,2992	0,4152	0,4229	0,4302
CCL3	0,38848	5,889	5,727	0,1617	0,1713	0,4106
Gal-1	0,406943	5,403	5,479	-0,07606	0,08404	0,3905
MCP-3	0,431858	2,675	2,592	0,08278	0,09688	0,3647
CAIX	0,457998	4,081	4,007	0,07386	0,09188	0,3391
HGF	0,458256	7,243	7,373	-0,1299	0,1617	0,3389
ANGPT2	0,467857	4,818	4,754	0,06389	0,08136	0,3299
IL8	0,48998	6,014	6,269	-0,2552	0,3427	0,3098
PD-L1	0,492254	5,724	5,56	0,1645	0,2221	0,3078
GZMA	0,544751	5,685	5,531	0,1538	0,2369	0,2638
MIC-A/B	0,569648	2,918	3,016	-0,09795	0,1611	0,2444
CCL4	0,588778	6,331	6,435	-0,1047	0,1813	0,23
TRAIL	0,599321	7,649	7,561	0,08736	0,1559	0,2223
MCP-2	0,630225	7,736	7,891	-0,1557	0,3039	0,2005
MCP-4	0,69238	11,61	11,7	-0,08814	0,2102	0,1597
IL10	0,699851	3,052	3,142	-0,09024	0,2209	0,155
CD27	0,708753	7,335	7,38	-0,0446	0,1128	0,1495
CXCL13	0,718525	7,172	7,275	-0,1031	0,2703	0,1436
ADGRG1	0,722068	1,002	0,9497	0,05257	0,1397	0,1414

PDCD1 (PD-1)	0,735279	5,776	5,697	0,07917	0,2214	0,1335
CD5	0,771151	4,606	4,577	0,02882	0,09384	0,1129
CCL19	0,844344	9,932	9,987	-0,05443	0,2632	0,07348
TIE2	0,856069	5,92	5,9	0,02064	0,1081	0,06749
ICOSLG	0,858256	3,584	3,606	-0,02184	0,1162	0,06638
IL6	0,870592	3,97	3,882	0,0872	0,5086	0,06019
DCN (Decorin)	0,871878	1,638	1,654	-0,01602	0,09441	0,05954
4-1BB	0,875128	4,764	4,784	-0,01977	0,1196	0,05793
TNFRSF12A (TWEAKR)	0,945886	4,424	4,435	-0,01092	0,1531	0,02416
ADA	0,963392	4,06	4,054	0,006692	0,1387	0,0162

**Heatmap differences in Fig 6b**	P value	Mean of PR	Mean of PD	Difference	SE of difference	-log10(P value)
MMP7	0,000619	11,47	11,99	-0,5201	0,11	3,208
ANGPT1	0,001633	7,148	8,281	-1,133	0,2734	2,787
CASP-8	0,003285	3,894	4,495	-0,6008	0,1608	2,483
CD40L	0,004317	4,662	5,697	-1,036	0,2892	2,365
EGF	0,008768	6,904	7,941	-1,037	0,3262	2,057
CAIX	0,012562	4,081	5,478	-1,397	0,4691	1,901
LAP TGF-beta-1	0,013871	7,583	8,285	-0,7014	0,24	1,858
TNFSF14 (LIGHT)	0,015506	3,535	4,6	-1,065	0,3723	1,81
MCP-3	0,015969	2,675	3,539	-0,8642	0,3039	1,797
MUC-16	0,015976	3,578	5,506	-1,928	0,678	1,797
IL7	0,017332	1,306	1,967	-0,6608	0,2362	1,761
PDGF subunit B	0,017541	8,694	9,268	-0,5743	0,2057	1,756
TIE2	0,029174	5,92	6,205	-0,2844	0,1135	1,535
TNFRSF12A (TWEAKR)	0,031633	4,424	4,916	-0,4912	0,1996	1,5
CXCL15	0,052751	8,943	10,05	-1,105	0,5093	1,278
CD244	0,080331	4,493	4,858	-0,3653	0,1897	1,095
TWEAK	0,085278	7,272	7,671	-0,3998	0,2114	1,069
MIC-A/B	0,088322	2,918	4,627	-1,709	0,9138	1,054
CCL17	0,090659	6,95	7,837	-0,8873	0,4785	1,043
CCL20	0,143932	7,597	8,312	-0,715	0,4544	0,8418

CD83	0,175173	1,451	1,718	-0,267	0,1842	0,7565
IL12RB1	0,178964	1,435	1,786	-0,351	0,2445	0,7472
IL8	0,190627	6,014	6,856	-0,8423	0,6039	0,7198
CXCL1	0,201389	8,102	8,53	-0,4286	0,3154	0,696
PDCD1 (PD-1)	0,214374	5,776	6,172	-0,3959	0,3005	0,6688
MMP12	0,223388	5,968	6,746	-0,7775	0,6025	0,6509
IFNg	0,231279	8,991	7,878	1,113	0,8787	0,6359
VEGFA	0,250788	8,783	8,959	-0,1759	0,1451	0,6007
HGF	0,253718	7,243	7,554	-0,311	0,2583	0,5956
LAG3	0,272437	3,302	3,029	0,2731	0,2364	0,5647
Gal-9	0,28479	7,598	7,381	0,2169	0,1929	0,5455
Gal-1	0,289283	5,403	5,508	-0,1047	0,09404	0,5387
IL15	0,317917	3,323	3,515	-0,1916	0,1832	0,4977
FASLG	0,387795	4,749	4,966	-0,2169	0,2412	0,4114
IL18	0,398767	9,576	9,242	0,3338	0,3802	0,3993
ADGRG1	0,402205	1,002	1,411	-0,4088	0,4691	0,3956
CD40	0,413797	10,11	10,25	-0,1412	0,1662	0,3832
TNF	0,443598	3,526	3,816	-0,2898	0,3647	0,353
CD70	0,465796	2,791	2,962	-0,1714	0,2269	0,3318
TNFRSF4 (OX40R)	0,468691	5,548	5,363	0,1844	0,2457	0,3291
IL6	0,474392	3,97	4,32	-0,3506	0,4733	0,3239
MCP-1	0,475535	11,31	11,05	0,254	0,3439	0,3228
PD-L2	0,482851	1,124	1,302	-0,1778	0,2448	0,3162
PGF	0,486604	7,973	7,845	0,1286	0,1786	0,3128
ICOSLG	0,511989	3,584	3,655	-0,07057	0,1041	0,2907
CD27	0,522765	7,335	7,459	-0,1242	0,1882	0,2817
VEGFR-2	0,530155	6,555	6,641	-0,08549	0,1319	0,2756
DCN (Decorin)	0,551791	1,638	1,727	-0,08879	0,1446	0,2582
4-1BB	0,558445	4,764	4,592	0,1726	0,2861	0,253
IL12	0,566106	3,999	4,34	-0,3404	0,5754	0,2471
CCL19	0,571096	9,932	9,726	0,2059	0,3527	0,2433
CD4	0,583148	3,054	2,975	0,07946	0,1405	0,2342
CD5	0,586508	4,606	4,7	-0,09425	0,1682	0,2317
CXCL11	0,604163	10,52	10,76	-0,2361	0,4423	0,2188

CCL23	0,625465	10,21	10,08	0,124	0,2469	0,2038
PD-L1	0,632118	5,724	5,625	0,099	0,201	0,1992
ADA	0,642874	4,06	4,147	-0,087	0,1825	0,1919
CXCL9	0,653764	6,997	7,265	-0,2676	0,5805	0,1846
GZMA	0,702739	5,685	5,794	-0,1089	0,278	0,1532
CXCL10	0,722793	9,018	8,802	0,2162	0,594	0,141
CCL3	0,72537	5,889	5,97	-0,08082	0,2243	0,1394
GZMH	0,725876	5,18	5,404	-0,2237	0,6218	0,1391
NOS3	0,748004	0,7144	0,8463	-0,1319	0,4004	0,1261
IL10	0,749065	3,052	2,772	0,2801	0,854	0,1255
TRAIL	0,75871	7,649	7,712	-0,06291	0,1998	0,1199
CSF-1	0,763998	9,086	9,06	0,02633	0,08554	0,1169
ANGPT2	0,768015	4,818	4,883	-0,06552	0,2167	0,1146
LAMP3	0,773338	4,752	4,919	-0,1667	0,5646	0,1116
CXCL13	0,780475	7,172	7,345	-0,1733	0,6069	0,1076
CCL4	0,78712	6,331	6,426	-0,09579	0,3461	0,104
KLRD1	0,79114	6,045	6,117	-0,07209	0,2656	0,1017
MCP-2	0,824303	7,736	7,679	0,05654	0,2487	0,08391
GZMB	0,836988	3,022	2,945	0,07784	0,3695	0,07728
CD8A	0,856827	9,36	9,285	0,07505	0,4064	0,06711
NCR1	0,869109	1,955	2,007	-0,05183	0,3073	0,06093
CRTAM	0,888629	4,718	4,768	-0,05001	0,3489	0,05128
HO-1	0,898235	12,42	12,47	-0,04436	0,3389	0,04661
MCP-4	0,904231	11,61	11,66	-0,04501	0,3656	0,04372
TNFRSF21 (Death receptor 6)	0,929409	7,648	7,659	-0,01011	0,1116	0,03179
CX3CL1	0,970557	3,156	3,148	0,008608	0,228	0,01298

**Heatmap differences Fig 6c**	P value	Mean of PR	Mean of PD	Difference	SE of difference	-log10(P value)
IL12	0,003485	-2,043	-0,1906	-1,852	0,5002	2,458
ANGPT1	0,004326	-1,222	-0,2853	-0,9363	0,2616	2,364
CD83	0,005173	-0,4188	0,2068	-0,6256	0,1799	2,286
MMP7	0,006803	-0,3387	0,03551	-0,3743	0,1126	2,167
ANGPT2	0,007023	0,06389	0,5967	-0,5328	0,1613	2,153

MIC-A/B	0,007793	-0,09795	0,4702	-0,5682	0,175	2,108
CXCL15	0,009521	-1,582	-0,4946	-1,088	0,3471	2,021
VEGFR-2	0,010746	-0,2062	0,1463	-0,3525	0,115	1,969
CD5	0,013089	0,02882	0,5124	-0,4836	0,1636	1,883
MUC-16	0,01622	-0,8401	0,6845	-1,525	0,5377	1,79
LAP TGF-beta-1	0,016794	-0,6292	0,09248	-0,7217	0,2563	1,775
MCP-3	0,018584	0,08278	0,756	-0,6733	0,244	1,731
IL7	0,018748	-0,8039	-0,04135	-0,7625	0,2769	1,727
IL6	0,020726	0,0872	1,683	-1,596	0,5916	1,683
CAIX	0,021289	0,07386	0,5804	-0,5065	0,1888	1,672
IL12RB1	0,023414	0,2072	0,8892	-0,682	0,2593	1,631
PDGF subunit B	0,027684	-0,7385	-0,2066	-0,5319	0,2098	1,558
ADGRG1	0,032127	0,05257	0,5896	-0,537	0,219	1,493
VEGFA	0,032284	-0,2468	0,2943	-0,541	0,2209	1,491
TNFSF14 (LIGHT)	0,036933	-0,8278	0,005687	-0,8335	0,3512	1,433
CD70	0,039502	-0,1969	0,3855	-0,5823	0,2494	1,403
CCL17	0,04819	-1,832	-0,7707	-1,062	0,4779	1,317
TNF	0,050485	0,2122	0,8254	-0,6132	0,2793	1,297
NCR1	0,052539	-0,3059	0,1392	-0,4452	0,2049	1,28
TIE2	0,053236	0,02064	0,3329	-0,3122	0,1442	1,274
MMP12	0,056432	-0,9319	-0,2015	-0,7304	0,3427	1,248
IL8	0,056949	-0,2552	0,5699	-0,8251	0,388	1,245
FASLG	0,073717	-0,4646	0,1263	-0,5909	0,299	1,132
CCL19	0,082125	-0,05443	0,5623	-0,6167	0,3224	1,086
TNFRSF12A (TWEAKR)	0,087075	-0,01092	0,3629	-0,3738	0,199	1,06
CXCL13	0,087309	-0,1031	0,5822	-0,6853	0,3651	1,059
CD40L	0,094099	-1,517	-0,6329	-0,8836	0,4823	1,026
PDCD1 (PD-1)	0,099711	0,07917	0,6731	-0,5939	0,3304	1,001
CD4	0,104034	0,1358	0,3967	-0,2608	0,1472	0,9828
EGF	0,104155	-1,471	-0,7373	-0,7337	0,4142	0,9823
CD27	0,112349	-0,04461	0,3439	-0,3885	0,2251	0,9494
CCL20	0,116058	-0,5167	0,3127	-0,8294	0,4862	0,9353
PD-L2	0,118247	0,1288	0,4585	-0,3296	0,1945	0,9272
4-1BB	0,122194	-0,01977	0,4341	-0,4539	0,271	0,9129

LAMP3	0,122448	0,2757	1,049	-0,7733	0,4622	0,912
CXCL9	0,135099	0,417	1,293	-0,8757	0,543	0,8693
CD244	0,144364	-0,5556	-0,1216	-0,434	0,2761	0,8405
HGF	0,154128	-0,1299	0,2084	-0,3383	0,221	0,8121
CCL3	0,181661	0,1617	0,5448	-0,383	0,2686	0,7407
KLRD1	0,187666	0,2931	0,7162	-0,423	0,3011	0,7266
HO-1	0,222046	0,9391	0,6201	0,319	0,2464	0,6536
TWEAK	0,230774	-0,4783	-0,1723	-0,306	0,2412	0,6368
CXCL11	0,234779	0,4592	1,019	-0,5602	0,4456	0,6293
CCL23	0,248078	0,2033	0,4475	-0,2442	0,2002	0,6054
CRTAM	0,250871	0,2303	0,6138	-0,3835	0,3164	0,6005
GZMA	0,263809	0,1538	0,5499	-0,3961	0,3364	0,5787
CXCL10	0,265682	0,7046	1,455	-0,7508	0,6402	0,5756
CASP-8	0,277246	-0,7569	-0,3309	-0,426	0,3727	0,5571
CXCL1	0,287452	-0,3457	-0,02243	-0,3233	0,2892	0,5414
CSF-1	0,290044	0,2061	0,3364	-0,1303	0,1172	0,5375
IL10	0,315963	-0,09024	0,3482	-0,4384	0,4173	0,5004
MCP-2	0,334677	-0,1557	0,1886	-0,3443	0,3413	0,4754
LAG3	0,354801	0,3981	0,6137	-0,2156	0,2232	0,45
CD40	0,372215	-0,3246	-0,08962	-0,235	0,2526	0,4292
TNFRSF4 (OX40R)	0,406306	0,3331	0,5347	-0,2015	0,2334	0,3911
IL18	0,473131	0,7366	0,9904	-0,2538	0,3416	0,325
DCN (Decorin)	0,483498	-0,01602	0,05816	-0,07418	0,1023	0,3156
CCL4	0,492466	-0,1047	0,06552	-0,1702	0,2397	0,3076
ADA	0,524543	0,006692	0,1791	-0,1724	0,2624	0,2802
CX3CL1	0,526239	0,5906	0,4817	0,109	0,1665	0,2788
IL15	0,563418	0,6113	0,4807	0,1306	0,2192	0,2492
TRAIL	0,607845	0,08736	0,1808	-0,09343	0,1769	0,2162
IFNg	0,693145	2,341	1,859	0,4818	1,189	0,1592
Gal-1	0,714692	-0,07606	-0,01519	-0,06087	0,1623	0,1459
CD8A	0,746198	0,469	0,5734	-0,1044	0,3145	0,1271
GZMB	0,761491	0,377	0,5727	-0,1958	0,6292	0,1183
TNFRSF21 (Death receptor 6)	0,779223	0,1417	0,1795	-0,03781	0,1316	0,1083
PGF	0,809745	0,3339	0,3822	-0,04823	0,1956	0,09165

ICOSLG	0,841684	-0,02184	0,00513	-0,02697	0,1319	0,07485
MCP-4	0,872523	-0,08814	-0,1355	0,04738	0,2885	0,05922
MCP-1	0,884129	0,662	0,6109	0,05115	0,3429	0,05348
NOS3	0,89652	0,4152	0,4806	-0,06542	0,4915	0,04744
GZMH	0,948332	0,8016	0,8748	-0,07325	1,105	0,02304
Gal-9	0,963301	0,461	0,4542	0,006801	0,1445	0,01624
PD-L1	0,982288	0,1645	0,157	0,007479	0,3293	0,007761

**Differences in Fig 6d**	P value	Mean of >Median OS	Mean of ≤Median OS	Difference	SE of difference	-log10(P value)
MMP7	0,003721	11,58	11,94	-0,3552	0,111	2,429
TNFSF14 (LIGHT)	0,004513	3,518	4,578	-1,06	0,3398	2,345
CSF-1	0,022087	8,974	9,133	-0,1587	0,06501	1,656
ANGPT2	0,026545	4,806	5,214	-0,4076	0,1729	1,576
HGF	0,034429	7,234	7,623	-0,3888	0,1738	1,463
CD83	0,0369	1,551	1,815	-0,2634	0,1195	1,433
CD70	0,040309	2,847	3,187	-0,3402	0,1573	1,395
LAP TGF-beta-1	0,045209	7,752	8,205	-0,4532	0,215	1,345
CCL20	0,045558	7,866	8,794	-0,9281	0,441	1,341
MIC-A/B	0,058305	3,651	4,645	-0,9932	0,5006	1,234
ANGPT1	0,08007	7,404	7,957	-0,553	0,3031	1,097
VEGFA	0,09081	8,808	9,084	-0,2768	0,1573	1,042
CASP-8	0,104128	3,992	4,448	-0,4559	0,2703	0,9824
TIE2	0,106335	6,074	6,251	-0,1768	0,1055	0,9733
CD244	0,110517	4,556	4,898	-0,342	0,2067	0,9566
CXCL15	0,130533	9,135	9,776	-0,6406	0,4097	0,8843
NOS3	0,13216	0,2487	0,6654	-0,4166	0,2677	0,8789
IL7	0,132737	1,322	1,737	-0,4152	0,2671	0,877
CAIX	0,133487	4,457	4,971	-0,5148	0,3319	0,8746
PD-L2	0,13508	1,214	1,423	-0,2084	0,1349	0,8694
CD5	0,14379	4,625	4,8	-0,1744	0,1155	0,8423
CCL17	0,144555	7,388	8,052	-0,6646	0,4413	0,84
IL8	0,152915	5,849	6,417	-0,5685	0,3856	0,8155
IL12	0,153735	3,917	4,421	-0,5039	0,3425	0,8132

CCL3	0,156404	5,716	6,037	-0,321	0,2197	0,8058
KLRD1	0,168012	5,756	6,101	-0,3443	0,2425	0,7747
CD27	0,172323	7,39	7,61	-0,2202	0,1567	0,7637
MCP-3	0,197907	2,92	3,194	-0,2742	0,2073	0,7035
EGF	0,200357	7,198	7,743	-0,5444	0,4139	0,6982
TNF	0,208259	3,54	3,835	-0,2954	0,2287	0,6814
CD40L	0,212309	4,959	5,525	-0,5657	0,442	0,673
PDGF subunit B	0,223947	8,781	9,052	-0,2713	0,2175	0,6499
TNFRSF21 (Death receptor 6)	0,230221	7,671	7,756	-0,0856	0,06961	0,6379
CXCL1	0,23947	8,009	8,301	-0,2914	0,2418	0,6207
MCP-1	0,244594	11,31	11,03	0,2879	0,2416	0,6116
Gal-9	0,2514	7,375	7,538	-0,1628	0,1386	0,5996
IL6	0,292818	3,801	4,392	-0,5901	0,5491	0,5334
IL15	0,305772	3,484	3,665	-0,1813	0,1734	0,5146
Gal-1	0,315556	5,415	5,493	-0,07809	0,07624	0,5009
ADGRG1	0,33221	0,8277	1,169	-0,3416	0,3454	0,4786
GZMA	0,344773	5,505	5,724	-0,2192	0,2276	0,4625
IL12RB1	0,345692	1,366	1,544	-0,1782	0,1854	0,4613
PD-L1	0,36357	5,499	5,693	-0,1939	0,2095	0,4394
VEGFR-2	0,369059	6,489	6,568	-0,07812	0,0854	0,4329
CD4	0,381025	2,962	3,056	-0,09424	0,1057	0,419
CCL19	0,384677	9,787	10,07	-0,2874	0,3249	0,4149
MMP12	0,385246	6,296	6,605	-0,3089	0,3496	0,4143
LAMP3	0,407284	4,787	4,535	0,2524	0,2995	0,3901
MCP-4	0,417598	11,89	11,68	0,2085	0,2529	0,3792
MUC-16	0,431477	3,757	4,218	-0,4612	0,5768	0,365
CD40	0,481366	10,06	10,18	-0,1209	0,1692	0,3175
IL18	0,493843	9,189	9,432	-0,2426	0,3493	0,3064
TRAIL	0,499306	7,555	7,637	-0,08248	0,1203	0,3016
ICOSLG	0,543061	3,653	3,706	-0,05294	0,08585	0,2652
CXCL10	0,557709	8,746	8,489	0,2569	0,4323	0,2536
HO-1	0,571219	12,39	12,29	0,1047	0,1825	0,2432
4-1BB	0,579254	4,693	4,799	-0,1063	0,1893	0,2371
TNFRSF12A (TWEAKR)	0,58551	4,729	4,823	-0,09403	0,1702	0,2325
NCR1	0,591553	2	2,121	-0,1207	0,2221	0,228

ADA	0,597752	4,01	4,126	-0,1162	0,2175	0,2235
CCL4	0,632575	6,367	6,479	-0,1126	0,2327	0,1989
CD8A	0,641004	9,332	9,181	0,1508	0,3195	0,1931
LAG3	0,695781	3,091	3,009	0,08243	0,2084	0,1575
CXCL13	0,716552	7,167	7,278	-0,1115	0,3036	0,1448
MCP-2	0,72058	7,679	7,61	0,06812	0,1883	0,1423
CCL23	0,737251	9,991	10,07	-0,07749	0,2284	0,1324
GZMH	0,776759	4,874	4,746	0,1278	0,446	0,1097
CXCL9	0,792355	6,961	6,86	0,1006	0,3782	0,1011
TNFRSF4 (OX40R)	0,802253	5,66	5,708	-0,04813	0,1901	0,09569
CRTAM	0,811133	4,784	4,839	-0,05518	0,2285	0,09091
IL10	0,815612	2,735	2,631	0,1044	0,443	0,08852
PGF	0,824339	7,988	7,954	0,03435	0,1532	0,08389
DCN (Decorin)	0,831653	1,669	1,687	-0,01854	0,08632	0,08006
IFNg	0,862481	8,105	8,005	0,0992	0,5668	0,06425
CX3CL1	0,866906	3,285	3,258	0,0274	0,1618	0,06203
GZMB	0,872191	2,888	2,827	0,061	0,3753	0,05939
FASLG	0,875961	4,889	4,857	0,03173	0,2012	0,05752
TWEAK	0,883352	7,583	7,612	-0,02858	0,1928	0,05387
PDCD1 (PD-1)	0,886959	5,975	5,947	0,02823	0,1965	0,0521
CXCL11	0,992815	10,57	10,57	-0,00256	0,2814	0,003132

**Differences in Fig 7a PD**	P value	Mean of w7	Mean of w1	Difference	SE of difference	-log10(P value)
MIC-A/B	0,000122	5,478	5,015	0,463	0,0528	3,914
FCRLB	0,000269	2,462	1,742	0,7204	0,0947	3,571
SYND1	0,000679	9,19	8,141	1,048	0,1635	3,168
ERBB3	0,001139	8,448	8,242	0,2059	0,03542	2,944
EPHA2	0,001982	4,167	3,704	0,4636	0,08886	2,703
WISP-1	0,002324	6,656	6,274	0,3817	0,07553	2,634
SCF	0,003001	7,661	8,12	-0,4597	0,09577	2,523
TNFRSF6B	0,003369	5,613	4,729	0,8841	0,1886	2,473
PVRL4	0,004134	6,153	5,774	0,3788	0,08429	2,384

IL6	0,00438	4,79	3,154	1,636	0,3686	2,359
LY9	0,006817	5,991	5,605	0,3859	0,09557	2,166
TNFRSF4	0,007084	6,411	5,939	0,472	0,1179	2,15
CD48	0,007421	7,015	6,729	0,2867	0,07234	2,13
ADAM 8	0,007669	6,34	5,95	0,39	0,09912	2,115
TFPI-2	0,009187	8,75	8,162	0,5876	0,1555	2,037
VEGFA	0,009362	10,66	10,26	0,3953	0,1051	2,029
CAIX	0,010186	5,926	5,322	0,6047	0,1638	1,992
AREG	0,01108	3,66	3,246	0,4141	0,1143	1,955
WFDC2	0,012085	7,694	7,288	0,4055	0,1143	1,918
IFNgR1	0,015167	6,782	6,613	0,1692	0,05031	1,819
CEACAM5	0,015914	5,336	4,399	0,9365	0,2817	1,798
TNFSF13	0,016626	8,944	8,705	0,2386	0,07253	1,779
DLL1	0,024907	10,83	10,55	0,2827	0,09515	1,604
TLR3	0,027843	4,191	4,007	0,1834	0,06355	1,555
EGF	0,028161	8,146	8,726	-0,5794	0,2014	1,55
S100A11	0,029257	4,037	3,688	0,3485	0,1224	1,534
CTSV	0,038522	0,5201	0,9063	-0,3862	0,1463	1,414
MUC-16	0,04278	6,607	5,953	0,6535	0,255	1,369
ADAM-TS 15	0,04477	4,276	4,005	0,271	0,1072	1,349
MSLN	0,046403	4,714	3,892	0,8215	0,3283	1,333
ITGAV	0,048067	4,559	4,726	-0,1673	0,06756	1,318
CD27	0,048156	8,008	7,675	0,3328	0,1345	1,317
VEGFR-3	0,052439	7,415	7,248	0,167	0,06926	1,28
5'-NT	0,054151	11,32	10,94	0,3832	0,1604	1,266
FURIN	0,059539	7,413	7,207	0,2051	0,08844	1,225
CXCL13	0,061749	7,627	7,075	0,5521	0,2409	1,209
hK11	0,071099	4,969	4,816	0,1531	0,06993	1,148
CXL17	0,09459	5,715	5,324	0,3904	0,1968	1,024
TXLNA	0,095261	3,367	3,026	0,3409	0,1723	1,021
CD70	0,096743	3,913	3,502	0,411	0,209	1,014
TGF-alpha	0,097296	3,313	2,914	0,3983	0,2029	1,012
LYN	0,098996	2,873	2,618	0,2553	0,1309	1,004
GPC1	0,100887	4,515	4,315	0,1999	0,1032	0,9962
TGFR-2	0,11704	7,364	7,201	0,1626	0,08886	0,9317

TRAIL	0,119968	8,264	8,091	0,1732	0,09561	0,9209
ERBB2	0,122984	5,872	5,724	0,1481	0,08256	0,9102
FR-alpha	0,136239	9,274	9,006	0,2681	0,1559	0,8657
VEGFR-2	0,158283	7,086	7,005	0,08111	0,05034	0,8006
SPARC	0,163889	9,484	9,358	0,1253	0,07901	0,7854
ITGB5	0,187319	7,687	7,931	-0,2434	0,1636	0,7274
TNFRSF19	0,20509	5,594	5,394	0,1993	0,1402	0,6881
MIA	0,220761	10,09	10,05	0,04144	0,03032	0,6561
SCAMP3	0,247463	7,518	7,974	-0,4566	0,3564	0,6065
PODXL	0,254911	4,694	4,623	0,0716	0,05689	0,5936
CD207	0,260196	2,688	2,946	-0,2583	0,2078	0,5847
FASLG	0,263419	8,937	8,825	0,1118	0,09061	0,5794
ABL1	0,300591	4,931	5,36	-0,4288	0,3786	0,522
MK	0,30227	6,724	6,505	0,2194	0,1945	0,5196
ERBB4	0,303028	10,66	10,58	0,0788	0,06995	0,5185
MetAP 2	0,30707	3,757	4,146	-0,3884	0,348	0,5128
RET	0,307489	5,412	5,26	0,1515	0,1358	0,5122
CDKN1A	0,334342	4,351	4,716	-0,3644	0,3472	0,4758
HGF	0,341276	8,269	8,06	0,2092	0,2024	0,4669
GZMH	0,369724	6,188	5,38	0,8074	0,8328	0,4321
S100A4	0,38389	5,793	6,007	-0,2137	0,2275	0,4158
FGF-BP1	0,387674	4,776	4,868	-0,09295	0,09982	0,4115
XPNPEP2	0,457311	8,963	9,049	-0,0856	0,1078	0,3398
KLK13	0,485017	3,02	2,838	0,1824	0,2451	0,3142
TCL1A	0,485573	5,602	6,05	-0,4476	0,6024	0,3137
GZMB	0,519173	4,455	4,056	0,3993	0,5833	0,2847
Gal-1	0,525173	7,072	7,133	-0,06154	0,09125	0,2797
FR-gamma	0,528117	7,674	7,614	0,05993	0,08953	0,2773
CYR61	0,536214	5,035	4,967	0,06781	0,1034	0,2707
CD160	0,545776	5,234	5,157	0,07645	0,1194	0,263
IGF1R	0,563844	4,077	4,011	0,06516	0,1067	0,2488
CPE	0,630647	3,94	3,882	0,05832	0,1152	0,2002
VIM	0,634637	3,903	4,144	-0,2412	0,482	0,1975
hK8	0,700189	5,438	5,506	-0,06759	0,1673	0,1548

GPNMB	0,720981	5,605	5,578	0,02686	0,07174	0,1421
WIF-1	0,732643	5,484	5,511	-0,02693	0,07525	0,1351
ICOSLG	0,813312	4,965	4,979	-0,01378	0,05585	0,08974
RSPO3	0,837418	5,669	5,647	0,02222	0,1037	0,07706
ANXA1	0,856212	4,137	4,187	-0,05028	0,2658	0,06742
hK14	0,883994	5,396	5,386	0,01048	0,06883	0,05355
MAD homolog 5	0,891347	2,359	2,335	0,02429	0,1704	0,04995
CRNN	0,92955	4,505	4,493	0,01198	0,1299	0,03173
PPY	0,946805	7,051	7,024	0,0262	0,3767	0,02374
ESM-1	0,962313	10,47	10,47	0,007037	0,1429	0,01668
LYPD3	0,968229	6,218	6,22	-0,002529	0,0609	0,01402
CEACAM1	0,972101	8,415	8,417	-0,002316	0,06352	0,01229
SEZ6L	0,98317	5,197	5,2	-0,002459	0,1118	0,007371

**Differences in Fig 7a SD**	P value	Mean of w7	Mean of w1	Difference	SE of difference	-log10(P value)
TGFR-2	0,0004	7,595	7,337	0,2582	0,05159	3,398
RSPO3	0,000401	6,221	5,839	0,3814	0,07624	3,396
CTSV	0,00052	0,8256	1,043	-0,2172	0,04488	3,284
TNFRSF4	0,001119	6,922	6,495	0,4268	0,09769	2,951
CPE	0,002559	4,27	4,029	0,2413	0,06217	2,592
TNFSF13	0,002782	9,065	8,723	0,3419	0,08921	2,556
TNFRSF6B	0,005357	5,429	4,922	0,5073	0,1467	2,271
TNFRSF19	0,006746	5,868	5,533	0,3356	0,1009	2,171
FR-alpha	0,008263	9,28	8,886	0,3932	0,1224	2,083
ADAM 8	0,008693	5,651	5,919	-0,268	0,08417	2,061
CEACAM1	0,010958	8,393	8,259	0,134	0,04385	1,96
ITGAV	0,014844	4,571	4,711	-0,1395	0,04835	1,828
DLL1	0,017005	11,01	10,82	0,1972	0,0702	1,769
WISP-1	0,01837	6,709	6,468	0,2413	0,08723	1,736
CD70	0,018671	4,205	3,897	0,3085	0,1119	1,729
ERBB3	0,019751	8,197	8,052	0,1447	0,0531	1,704
VEGFR-3	0,021499	7,291	7,184	0,107	0,03997	1,668
LYPD3	0,021841	6,071	6,17	-0,09856	0,03693	1,661

CXL17	0,021835	5,628	5,422	0,2053	0,07691	1,661
CD207	0,024883	2,592	3,069	-0,4764	0,1835	1,604
SCF	0,026416	7,781	8,119	-0,3382	0,132	1,578
TFPI-2	0,026698	7,942	7,764	0,178	0,06965	1,574
FASLG	0,032068	8,741	9,051	-0,3097	0,1262	1,494
FCRLB	0,037402	2,346	2,124	0,2224	0,09399	1,427
GPC1	0,039397	4,583	4,447	0,1362	0,05826	1,405
CXCL13	0,043566	7,472	7,249	0,2231	0,09785	1,361
SYND1	0,050287	7,807	7,53	0,2772	0,1261	1,299
Gal-1	0,061542	7,031	6,92	0,1104	0,05305	1,211
VEGFA	0,061931	10,55	10,32	0,23	0,1107	1,208
MK	0,070777	6,564	6,358	0,2058	0,1029	1,15
VEGFR-2	0,075483	6,926	6,852	0,07387	0,03764	1,122
ANXA1	0,076826	3,761	4,029	-0,2683	0,1374	1,114
TGF-alpha	0,079231	2,722	2,919	-0,1966	0,1017	1,101
IGF1R	0,09269	4,048	3,846	0,2017	0,1096	1,033
CD27	0,094251	8,104	8,007	0,09642	0,05265	1,026
TXLNA	0,09601	3,056	2,727	0,3297	0,1812	1,018
CRNN	0,096903	4,549	4,808	-0,2596	0,1431	1,014
PPY	0,130691	7,75	7,378	0,3725	0,2281	0,8838
MIC-A/B	0,131286	5,121	4,996	0,1249	0,07659	0,8818
MAD homolog 5	0,136784	2,716	2,501	0,2151	0,134	0,864
AREG	0,137047	3,551	3,333	0,218	0,1359	0,8631
EGF	0,144055	7,456	7,862	-0,4057	0,2579	0,8415
5'-NT	0,144226	10,37	10,14	0,2279	0,145	0,841
WFDC2	0,147875	7,212	7,088	0,124	0,07968	0,8301
hK14	0,15443	5,583	5,468	0,1148	0,07509	0,8113
WIF-1	0,158238	5,547	5,348	0,1995	0,1318	0,8007
IFNgR1	0,174513	6,632	6,558	0,07393	0,05093	0,7582
CD48	0,185906	6,918	6,842	0,07612	0,05395	0,7307
S100A11	0,202938	3,641	3,548	0,09264	0,06842	0,6926
VIM	0,206811	3,523	3,942	-0,4189	0,3122	0,6844
hK11	0,239073	5,035	4,947	0,08871	0,07126	0,6215
MSLN	0,239117	4,063	3,91	0,1531	0,123	0,6214

TLR3	0,243743	4,601	4,458	0,1425	0,1157	0,6131
LYN	0,259654	2,641	2,501	0,1394	0,1173	0,5856
ICOSLG	0,259786	5,102	5,045	0,05707	0,04803	0,5854
PODXL	0,27809	4,294	4,339	-0,04576	0,0401	0,5558
XPNPEP2	0,280224	8,56	8,498	0,06146	0,05411	0,5525
FGF-BP1	0,317671	4,582	4,776	-0,1935	0,1849	0,498
SCAMP3	0,32146	7,128	7,312	-0,1838	0,177	0,4929
CEACAM5	0,345139	3,128	3,284	-0,1565	0,1586	0,462
EPHA2	0,352311	3,963	3,868	0,09427	0,09707	0,4531
TRAIL	0,371432	8,007	7,907	0,09993	0,1072	0,4301
ADAM-TS 15	0,37531	4,476	4,383	0,09305	0,1007	0,4256
KLK13	0,38879	3,315	3,213	0,1024	0,1141	0,4103
LY9	0,409356	5,823	5,762	0,0606	0,07066	0,3879
S100A4	0,412633	5,843	5,727	0,1152	0,1353	0,3844
GPNMB	0,42221	5,774	5,727	0,04636	0,05561	0,3745
CD160	0,425639	5,276	5,356	-0,07991	0,09659	0,371
MUC-16	0,466793	4,345	4,147	0,1981	0,2628	0,3309
CAIX	0,467645	4,995	4,816	0,1796	0,2387	0,3301
TCL1A	0,490154	5,36	5,566	-0,2051	0,2873	0,3097
HGF	0,528697	8,005	7,943	0,06186	0,0951	0,2768
CDKN1A	0,542706	4,207	4,011	0,1959	0,3118	0,2654
CYR61	0,566361	5,06	4,996	0,06367	0,1077	0,2469
SPARC	0,616577	9,397	9,417	-0,02036	0,03951	0,21
ABL1	0,62717	4,873	4,738	0,1345	0,2693	0,2026
ERBB4	0,652502	10,45	10,42	0,02158	0,04662	0,1854
ESM-1	0,667027	10,44	10,48	-0,04361	0,09865	0,1759
hK8	0,680396	5,567	5,598	-0,03076	0,0727	0,1672
MetAP 2	0,755305	3,687	3,737	-0,05004	0,1566	0,1219
FR-gamma	0,763891	8,608	8,568	0,04091	0,1329	0,117
ERBB2	0,768885	5,68	5,698	-0,01743	0,05786	0,1141
IL6	0,780243	4,403	4,469	-0,06579	0,2301	0,1078
SEZ6L	0,859152	5,264	5,274	-0,01048	0,05766	0,06593
MIA	0,880189	9,822	9,831	-0,008788	0,05697	0,05542
RET	0,894815	5,22	5,233	-0,01292	0,09546	0,04827
GZMH	0,897319	5,145	5,102	0,04305	0,326	0,04705

FURIN	0,938372	7,193	7,204	-0,01046	0,1322	0,02762
ITGB5	0,952685	7,765	7,755	0,009893	0,163	0,02105
PVRL4	0,965865	5,42	5,423	-0,003482	0,07953	0,01508
GZMB	0,996704	4,208	4,207	0,00115	0,2721	0,001434

**Differences in Fig 7a PR**	P value	Mean of w7	Mean of w1	Difference	SE of difference	-log10(P value)
ITGAV	0,000393	4,493	4,747	-0,2547	0,03035	3,405
CTSV	0,001449	0,6345	0,9928	-0,3583	0,05658	2,839
LYPD3	0,001625	5,843	6,218	-0,3753	0,06079	2,789
EGF	0,002155	6,964	8,56	-1,595	0,2753	2,667
ITGB5	0,002646	7,529	8,092	-0,5632	0,1018	2,577
CD207	0,00465	2,171	3,029	-0,8585	0,1768	2,333
SCAMP3	0,005468	6,936	7,912	-0,9758	0,2088	2,262
ERBB2	0,00704	5,682	5,944	-0,2626	0,05971	2,152
ADAM 8	0,013555	5,415	6,031	-0,6159	0,1651	1,868
IGF1R	0,019334	3,742	4,125	-0,3832	0,1128	1,714
TGF-alpha	0,019349	2,448	2,831	-0,3829	0,1128	1,713
MIA	0,024991	9,679	9,841	-0,1621	0,05124	1,602
WISP-1	0,026749	6,293	6,503	-0,2099	0,06765	1,573
FASLG	0,026851	8,721	9,165	-0,4437	0,1431	1,571
CDKN1A	0,033718	3,745	4,407	-0,6618	0,2281	1,472
S100A4	0,033926	5,637	6,001	-0,3635	0,1255	1,469
MetAP 2	0,038432	3,584	4,165	-0,581	0,2082	1,415
CEACAM5	0,041122	2,416	3,57	-1,154	0,4221	1,386
TNFRSF4	0,04227	6,627	6,237	0,3903	0,144	1,374
TGFR-2	0,054854	7,296	7,186	0,1098	0,04403	1,261
TNFSF13	0,054932	8,893	8,666	0,2267	0,09091	1,26
PODXL	0,058306	4,046	4,222	-0,1761	0,07204	1,234
ABL1	0,05957	4,367	5,123	-0,7558	0,3113	1,225
CYR61	0,06626	4,652	4,898	-0,2458	0,105	1,179
FURIN	0,069519	7,245	7,538	-0,2928	0,1271	1,158
GPNMB	0,078007	5,558	5,681	-0,1235	0,05586	1,108
hK8	0,080139	5,214	5,415	-0,201	0,09182	1,096

FR-alpha	0,086704	9,016	8,866	0,1496	0,07032	1,062
KLK13	0,113861	3,262	3,086	0,1761	0,09201	0,9436
MAD homolog 5	0,12104	2,407	2,565	-0,1581	0,08475	0,9171
MUC-16	0,125422	4,627	5,44	-0,8137	0,4426	0,9016
RET	0,132942	5,186	5,409	-0,223	0,1244	0,8763
WFDC2	0,135625	7,018	6,943	0,07544	0,04244	0,8677
hK14	0,136595	5,685	5,933	-0,2482	0,14	0,8646
PVRL4	0,146611	5,023	5,172	-0,149	0,08675	0,8338
CD160	0,151648	5,122	5,288	-0,1655	0,09785	0,8192
ERBB3	0,169326	8,072	8,206	-0,1339	0,08342	0,7713
HGF	0,169924	7,92	8,11	-0,1906	0,1189	0,7697
ERBB4	0,170739	10,37	10,52	-0,1502	0,09395	0,7677
CPE	0,180745	4,217	4,143	0,07421	0,04773	0,7429
IFNgR1	0,18534	6,429	6,505	-0,07566	0,04928	0,732
XPNPEP2	0,189784	8,867	8,964	-0,09734	0,06418	0,7217
VEGFA	0,215525	10,42	10,71	-0,2871	0,2025	0,6665
VEGFR-2	0,215874	7,103	7,217	-0,1142	0,08066	0,6658
MIC-A/B	0,235778	3,77	3,838	-0,06739	0,05003	0,6275
MK	0,244014	6,156	6,42	-0,2646	0,2004	0,6126
5'-NT	0,255575	10,86	10,59	0,2786	0,217	0,5925
WIF-1	0,258971	5,15	5,277	-0,1272	0,09994	0,5867
GZMH	0,262036	6,051	5,223	0,8285	0,6555	0,5816
ADAM-TS 15	0,291013	4,338	4,435	-0,097	0,08219	0,5361
LYN	0,300552	2,669	2,537	0,1315	0,114	0,5221
GZMB	0,336202	4,684	4,211	0,473	0,4447	0,4734
ESM-1	0,337926	10,3	10,42	-0,1175	0,1109	0,4712
FCRLB	0,340158	2,191	2,099	0,09157	0,08688	0,4683
ANXA1	0,361871	3,917	4,141	-0,2236	0,2229	0,4414
VEGFR-3	0,363276	7,353	7,411	-0,05741	0,05742	0,4398
TXLNA	0,374868	3,063	2,82	0,243	0,2495	0,4261
Gal-1	0,380589	7,029	7,074	-0,04467	0,04647	0,4195
TNFRSF19	0,422392	5,382	5,461	-0,07862	0,09001	0,3743
hK11	0,453257	4,826	4,787	0,03885	0,0478	0,3437
SPARC	0,46119	9,22	9,298	-0,07736	0,09696	0,3361

MSLN	0,471349	4,158	4,022	0,1359	0,1745	0,3267
CD70	0,478167	3,791	3,904	-0,1139	0,1487	0,3204
AREG	0,504689	2,754	2,922	-0,1683	0,2343	0,297
VIM	0,514378	3,572	3,78	-0,208	0,2966	0,2887
CXL17	0,519343	5,632	5,554	0,07749	0,1119	0,2845
GPC1	0,538875	4,374	4,42	-0,0466	0,07069	0,2685
FGF-BP1	0,57979	4,163	4,202	-0,03919	0,06624	0,2367
CAIX	0,581615	4,478	4,387	0,09132	0,1551	0,2354
SEZ6L	0,584557	5,001	5,04	-0,03853	0,06598	0,2332
PPY	0,637986	8,859	8,709	0,15	0,2997	0,1952
CXCL13	0,638579	7,503	7,594	-0,09153	0,1832	0,1948
LY9	0,647836	5,682	5,733	-0,05059	0,1042	0,1885
SYND1	0,70948	8,236	8,32	-0,0846	0,2144	0,1491
CD27	0,710854	7,861	7,904	-0,04352	0,1109	0,1482
FR-gamma	0,711379	7,882	7,834	0,04876	0,1245	0,1479
CRNN	0,728166	4,516	4,577	-0,06133	0,1668	0,1378
TRAIL	0,758224	8,112	8,054	0,05769	0,1774	0,1202
CD48	0,760506	6,826	6,807	0,0184	0,05714	0,1189
RSPO3	0,78413	5,811	5,838	-0,02698	0,09335	0,1056
TCL1A	0,785925	5,46	5,585	-0,1252	0,4369	0,1046
TLR3	0,818548	3,893	3,909	-0,01585	0,06554	0,08696
DLL1	0,854962	10,74	10,73	0,01274	0,0662	0,06805
EPHA2	0,866621	3,56	3,539	0,02096	0,1186	0,06217
S100A11	0,889895	3,733	3,699	0,03468	0,2381	0,05066
TFPI-2	0,891084	7,998	7,967	0,03082	0,214	0,05008
ICOSLG	0,901206	4,877	4,87	0,006435	0,04928	0,04518
IL6	0,908244	4,386	4,341	0,04442	0,3665	0,0418
SCF	0,925379	8,003	8,022	-0,01914	0,1943	0,03368
TNFRSF6B	0,971174	5,077	5,086	-0,009192	0,242	0,0127
CEACAM1	0,995796	8,427	8,427	-0,00025	0,04514	0,00183

**Differences in Fig 7d**	P value	Mean of PR	Mean of PD	Difference	SE of difference	-log10(P value)

PVRL4	0,000163	5,023	6,153	-1,13	0,2022	3,789
PODXL	0,000408	4,046	4,694	-0,6484	0,1299	3,39
ADAM 8	0,000579	5,415	6,34	-0,9249	0,1938	3,237
EGF	0,002041	6,964	8,146	-1,182	0,2945	2,69
AREG	0,002107	2,754	3,66	-0,9068	0,227	2,676
SYND1	0,008794	8,236	9,19	-0,9537	0,3001	2,056
EPHA2	0,010861	3,56	4,167	-0,6079	0,1987	1,964
CAIX	0,011922	4,478	5,926	-1,448	0,4815	1,924
WFDC2	0,01487	7,018	7,694	-0,6753	0,2342	1,828
TGF-alpha	0,018601	2,448	3,313	-0,865	0,3136	1,73
LYPD3	0,018914	5,843	6,218	-0,3751	0,1364	1,723
IGF1R	0,020569	3,742	4,077	-0,3345	0,1238	1,687
IFNgR1	0,020841	6,429	6,782	-0,3527	0,1309	1,681
MK	0,023281	6,156	6,724	-0,5683	0,2158	1,633
CEACAM5	0,023433	2,416	5,336	-2,919	1,11	1,63
TFPI-2	0,025037	7,998	8,75	-0,752	0,2901	1,601
ERBB3	0,027284	8,072	8,448	-0,3756	0,1476	1,564
MUC-16	0,032232	4,627	6,607	-1,98	0,8082	1,492
MIA	0,03586	9,679	10,09	-0,4162	0,1741	1,445
WIF-1	0,048809	5,15	5,484	-0,3342	0,1509	1,311
SPARC	0,056613	9,22	9,484	-0,2631	0,1235	1,247
ABL1	0,056651	4,367	4,931	-0,564	0,2649	1,247
VEGFA	0,070511	10,42	10,66	-0,2375	0,1186	1,152
WISP-1	0,07273	6,293	6,656	-0,3631	0,183	1,138
SCAMP3	0,073873	6,936	7,518	-0,5815	0,2944	1,132
CYR61	0,080775	4,652	5,035	-0,3822	0,1988	1,093
SCF	0,083075	8,003	7,661	0,3426	0,1797	1,081
ERBB2	0,091355	5,682	5,872	-0,1901	0,1027	1,039
MIC-A/B	0,098413	3,77	5,478	-1,708	0,946	1,007
ERBB4	0,100424	10,37	10,66	-0,2909	0,1622	0,9982
FGF-BP1	0,106293	4,163	4,776	-0,6128	0,3484	0,9735
CDKN1A	0,115328	3,745	4,351	-0,6061	0,3545	0,9381
LY9	0,149999	5,682	5,991	-0,3089	0,1996	0,8239
S100A11	0,152635	3,733	4,037	-0,3036	0,1976	0,8163

TNFRSF6B	0,157348	5,077	5,613	-0,5365	0,3535	0,8031
HGF	0,168199	7,92	8,269	-0,3492	0,2367	0,7742
SEZ6L	0,172358	5,001	5,197	-0,1958	0,1342	0,7636
5'-NT	0,18444	10,86	11,32	-0,4571	0,3228	0,7341
PPY	0,20932	8,859	7,051	1,808	1,356	0,6792
CD207	0,216181	2,171	2,688	-0,5175	0,3944	0,6652
FURIN	0,239713	7,245	7,413	-0,1672	0,1345	0,6203
TXLNA	0,241518	3,063	3,367	-0,304	0,2455	0,6171
CPE	0,282915	4,217	3,94	0,2765	0,2449	0,5483
LYN	0,284316	2,669	2,873	-0,2043	0,1815	0,5462
CD48	0,284695	6,826	7,015	-0,1896	0,1686	0,5456
FCRLB	0,292048	2,191	2,462	-0,2717	0,2455	0,5345
ITGB5	0,312069	7,529	7,687	-0,158	0,1491	0,5057
MSLN	0,341444	4,158	4,714	-0,5555	0,5587	0,4667
RET	0,342602	5,186	5,412	-0,2254	0,2272	0,4652
FASLG	0,349976	8,721	8,937	-0,2156	0,2209	0,456
FR-alpha	0,382692	9,016	9,274	-0,2579	0,2836	0,4172
GPC1	0,383892	4,374	4,515	-0,1412	0,1557	0,4158
hK14	0,39636	5,685	5,396	0,2889	0,3273	0,4019
IL6	0,402082	4,386	4,79	-0,4047	0,4644	0,3957
hK11	0,420839	4,826	4,969	-0,1425	0,1705	0,3759
hK8	0,423902	5,214	5,438	-0,2244	0,2701	0,3727
VIM	0,461071	3,572	3,903	-0,3301	0,4321	0,3362
ICOSLG	0,464305	4,877	4,965	-0,08841	0,1166	0,3332
S100A4	0,467143	5,637	5,793	-0,1557	0,2067	0,3305
TNFRSF19	0,484633	5,382	5,594	-0,2116	0,2926	0,3146
CD27	0,493534	7,861	8,008	-0,147	0,2075	0,3067
ANXA1	0,515092	3,917	4,137	-0,2192	0,326	0,2881
TNFRSF4	0,517283	6,627	6,411	0,2158	0,3225	0,2863
TLR3	0,544867	3,893	4,191	-0,2973	0,4758	0,2637
TRAIL	0,550311	8,112	8,264	-0,1524	0,2473	0,2594
GZMB	0,560251	4,684	4,455	0,2284	0,3802	0,2516
MetAP 2	0,561005	3,584	3,757	-0,1738	0,2899	0,251
RSPO3	0,567053	5,811	5,669	0,1423	0,2412	0,2464

ITGAV	0,602462	4,493	4,559	-0,0661	0,1233	0,2201
DLL1	0,608992	10,74	10,83	-0,08625	0,1638	0,2154
KLK13	0,612211	3,262	3,02	0,2417	0,4632	0,2131
GPNMB	0,627354	5,558	5,605	-0,0474	0,09491	0,2025
ESM-1	0,638799	10,3	10,47	-0,1712	0,3546	0,1946
VEGFR-3	0,641973	7,353	7,415	-0,06182	0,1293	0,1925
CD70	0,656556	3,791	3,913	-0,122	0,267	0,1827
CD160	0,665546	5,122	5,234	-0,1115	0,2511	0,1768
Gal-1	0,683842	7,029	7,072	-0,04265	0,102	0,165
CTSV	0,70057	0,6345	0,5201	0,1145	0,29	0,1545
TNFSF13	0,747029	8,893	8,944	-0,05118	0,1547	0,1267
TGFR-2	0,771252	7,296	7,364	-0,06817	0,2288	0,1128
TCL1A	0,814247	5,46	5,602	-0,1422	0,5909	0,08924
GZMH	0,831519	6,051	6,188	-0,1362	0,6252	0,08013
ADAM-TS 15	0,835874	4,338	4,276	0,0614	0,2894	0,07786
XPNPEP2	0,842307	8,867	8,963	-0,09619	0,4722	0,07453
CXCL13	0,846342	7,503	7,627	-0,1242	0,6259	0,07245
MAD homolog 5	0,853193	2,407	2,359	0,04733	0,2498	0,06895
CXL17	0,854953	5,632	5,715	-0,08296	0,4433	0,06806
FR-gamma	0,85984	7,882	7,674	0,2086	1,154	0,06558
VEGFR-2	0,879722	7,103	7,086	0,01708	0,1103	0,05565
CEACAM1	0,884204	8,427	8,415	0,01243	0,08338	0,05345
CRNN	0,985128	4,516	4,505	0,01091	0,5722	0,006507

**Differences in Fig 7c**	P value	Mean of PR	Mean of PD	Difference	SE of difference	-log10(P value)
MIC-A/B	0,000017	-0,06739	0,463	-0,5304	0,07353	4,764
WISP-1	0,000129	-0,2099	0,3817	-0,5917	0,103	3,889
ADAM 8	0,000214	-0,6159	0,39	-1,006	0,186	3,669
FCRLB	0,00053	0,09157	0,7204	-0,6288	0,1303	3,276
PVRL4	0,001165	-0,149	0,3788	-0,5277	0,1215	2,934
LYPD3	0,00125	-0,3753	-0,002529	-0,3727	0,08662	2,903
SYND1	0,001323	-0,0846	1,048	-1,133	0,2654	2,879
CEACAM5	0,001413	-1,154	0,9365	-2,09	0,4942	2,85

ERBB3	0,002229	-0,1339	0,2059	-0,3398	0,08578	2,652
ERBB2	0,002459	-0,2626	0,1481	-0,4107	0,1052	2,609
MIA	0,004619	-0,1621	0,04144	-0,2036	0,05747	2,335
IFNgR1	0,005441	-0,07566	0,1692	-0,2449	0,07101	2,264
FASLG	0,006133	-0,4437	0,1118	-0,5555	0,1643	2,212
FURIN	0,007194	-0,2928	0,2051	-0,4979	0,1513	2,143
TGF-alpha	0,008372	-0,3829	0,3983	-0,7811	0,2437	2,077
VEGFA	0,009652	-0,2871	0,3953	-0,6824	0,2183	2,015
LY9	0,010293	-0,0506	0,3859	-0,4365	0,1413	1,987
IL6	0,011215	0,04442	1,636	-1,592	0,5234	1,95
EPHA2	0,01125	0,02096	0,4636	-0,4426	0,1456	1,949
EGF	0,011301	-1,595	-0,5794	-1,016	0,3345	1,947
MUC-16	0,012501	-0,8137	0,6535	-1,467	0,4922	1,903
TNFRSF6B	0,01313	-0,009192	0,8841	-0,8933	0,3025	1,882
IGF1R	0,014923	-0,3832	0,06516	-0,4483	0,1556	1,826
CD48	0,016148	0,0184	0,2867	-0,2683	0,09455	1,792
PODXL	0,019412	-0,1761	0,0716	-0,2477	0,09059	1,712
ADAM-TS 15	0,02257	-0,097	0,271	-0,368	0,1389	1,646
WFDC2	0,027707	0,07544	0,4055	-0,3301	0,1302	1,557
VEGFR-3	0,032738	-0,05742	0,167	-0,2245	0,09194	1,485
AREG	0,038962	-0,1683	0,4141	-0,5824	0,2485	1,409
CAIX	0,045808	0,09132	0,6047	-0,5134	0,2281	1,339
DLL1	0,045883	0,01274	0,2827	-0,27	0,12	1,338
TLR3	0,052399	-0,01585	0,1834	-0,1993	0,09165	1,281
CD207	0,05398	-0,8585	-0,2583	-0,6002	0,2782	1,268
TFPI-2	0,054886	0,03083	0,5876	-0,5568	0,2593	1,261
SCF	0,056451	-0,01914	-0,4597	0,4405	0,2067	1,248
VEGFR-2	0,057534	-0,1142	0,08111	-0,1953	0,09213	1,24
CYR61	0,05782	-0,2458	0,06781	-0,3136	0,1481	1,238
CD27	0,058385	-0,04352	0,3328	-0,3763	0,1782	1,234
CXCL13	0,063018	-0,09153	0,5521	-0,6437	0,3113	1,201
RET	0,07014	-0,223	0,1515	-0,3745	0,1867	1,154
ERBB4	0,07194	-0,1502	0,0788	-0,229	0,115	1,143
CD70	0,073218	-0,1139	0,411	-0,5249	0,2651	1,135
GPC1	0,083758	-0,0466	0,1999	-0,2465	0,1296	1,077

MSLN	0,107559	0,1359	0,8215	-0,6856	0,3913	0,9684
hK14	0,110158	-0,2482	0,01048	-0,2586	0,1488	0,958
MK	0,112227	-0,2646	0,2194	-0,484	0,2804	0,9499
SPARC	0,129783	-0,07736	0,1253	-0,2026	0,1237	0,8868
HGF	0,131997	-0,1906	0,2092	-0,3997	0,2457	0,8794
GPNMB	0,135572	-0,1235	0,02686	-0,1504	0,09336	0,8678
TNFRSF19	0,137369	-0,07862	0,1993	-0,2779	0,1734	0,8621
ITGB5	0,139779	-0,5632	-0,2434	-0,3198	0,2009	0,8546
CD160	0,153838	-0,1655	0,07645	-0,2419	0,1579	0,8129
CXL17	0,214259	0,07749	0,3904	-0,3129	0,2374	0,6691
hK11	0,219625	0,03885	0,1531	-0,1143	0,08779	0,6583
S100A11	0,245879	0,03468	0,3485	-0,3139	0,256	0,6093
SCAMP3	0,254942	-0,9758	-0,4566	-0,5193	0,4323	0,5936
ITGAV	0,289243	-0,2547	-0,1673	-0,08747	0,07856	0,5387
MAD homolog 5	0,383526	-0,1581	0,02429	-0,1824	0,201	0,4162
WIF-1	0,43205	-0,1272	-0,02693	-0,1003	0,123	0,3645
LYN	0,498208	0,1315	0,2553	-0,1237	0,1766	0,3026
CDKN1A	0,505175	-0,6618	-0,3644	-0,2974	0,4317	0,2966
ESM-1	0,516469	-0,1175	0,007037	-0,1245	0,1858	0,287
hK8	0,519324	-0,201	-0,06759	-0,1335	0,2005	0,2846
FR-alpha	0,526801	0,1496	0,2681	-0,1185	0,1814	0,2784
ABL1	0,527574	-0,7558	-0,4288	-0,327	0,5012	0,2777
TRAIL	0,562159	0,05769	0,1732	-0,1155	0,1933	0,2501
S100A4	0,594012	-0,3635	-0,2137	-0,1498	0,2729	0,2262
TGFR-2	0,624388	0,1098	0,1626	-0,05276	0,1047	0,2045
ANXA1	0,634201	-0,2236	-0,05028	-0,1733	0,3542	0,1978
MetAP 2	0,658584	-0,581	-0,3884	-0,1925	0,424	0,1814
TNFRSF4	0,665977	0,3903	0,472	-0,0817	0,1842	0,1765
FGF-BP1	0,674022	-0,03919	-0,09295	0,05376	0,1244	0,1713
TCL1A	0,682817	-0,1252	-0,4476	0,3224	0,7682	0,1657
5'-NT	0,700592	0,2786	0,3832	-0,1046	0,2649	0,1545
CRNN	0,731669	-0,06133	0,01198	-0,07331	0,2084	0,1357
RSPO3	0,734866	-0,02698	0,02222	-0,04921	0,1417	0,1338
TXLNA	0,747039	0,243	0,3409	-0,09793	0,2961	0,1267

ICOSLG	0,794465	0,006435	-0,01378	0,02022	0,07574	0,09993
SEZ6L	0,795468	-0,03853	-0,002459	-0,03607	0,1358	0,09938
PPY	0,806513	0,15	0,0262	0,1238	0,4935	0,09339
CTSV	0,870366	-0,3583	-0,3862	0,02795	0,1673	0,0603
Gal-1	0,878672	-0,04467	-0,06154	0,01687	0,108	0,05617
CPE	0,906866	0,07421	0,05832	0,01588	0,1327	0,04246
TNFSF13	0,919379	0,2267	0,2386	-0,0119	0,1149	0,03651
GZMB	0,924006	0,473	0,3993	0,07363	0,7544	0,03433
XPNPEP2	0,930261	-0,09734	-0,0856	-0,01174	0,1312	0,0314
FR-gamma	0,942008	0,04876	0,05993	-0,01118	0,1502	0,02595
VIM	0,956209	-0,208	-0,2412	0,03318	0,5906	0,01945
CEACAM1	0,98	-0,00025	-0,002316	0,002066	0,08055	0,008774
KLK13	0,982425	0,1761	0,1824	-0,0063	0,2796	0,007701
GZMH	0,984898	0,8285	0,8074	0,02106	1,087	0,006609

**Differences in Fig 7d**	P value	Mean of >Median OS	Mean of ≤Median OS	Difference	SE of difference	-log10(P value)
ERBB3	0,001224	8,085	8,424	-0,3395	0,09313	2,912
ADAM 8	0,003098	5,529	6,149	-0,6207	0,1896	2,509
CD70	0,003657	3,769	4,292	-0,5235	0,1632	2,437
PVRL4	0,004362	5,264	5,928	-0,6634	0,2116	2,36
IFNgR1	0,004444	6,511	6,78	-0,2695	0,0862	2,352
HGF	0,004515	7,867	8,366	-0,499	0,1599	2,345
VEGFR-3	0,005019	7,237	7,443	-0,2053	0,06673	2,299
FURIN	0,005666	7,086	7,475	-0,3887	0,1284	2,247
RET	0,006631	5,077	5,481	-0,404	0,1364	2,178
VEGFA	0,00931	10,38	10,81	-0,4345	0,1542	2,031
WISP-1	0,009683	6,416	6,811	-0,3945	0,1408	2,014
AREG	0,012492	3,099	3,916	-0,8172	0,3036	1,903
TGF-alpha	0,013726	2,588	3,205	-0,6171	0,2328	1,862
TXLNA	0,014843	2,901	3,468	-0,5665	0,2165	1,828
IGF1R	0,019495	3,845	4,186	-0,3412	0,1367	1,71
EPHA2	0,021056	3,762	4,155	-0,393	0,1596	1,677

MK	0,022692	6,309	6,744	-0,4347	0,179	1,644
TNFRSF6B	0,023118	5,139	5,709	-0,5695	0,2353	1,636
SCF	0,033176	7,977	7,426	0,5509	0,2444	1,479
MIC-A/B	0,041755	4,428	5,516	-1,088	0,5072	1,379
EGF	0,058714	7,209	8,013	-0,804	0,4059	1,231
CDKN1A	0,060219	3,839	4,579	-0,7401	0,376	1,22
CD27	0,061104	7,892	8,196	-0,3042	0,1551	1,214
CAIX	0,063043	4,797	5,446	-0,6492	0,3337	1,2
ANXA1	0,07254	3,776	4,164	-0,3883	0,2071	1,139
SYND1	0,088184	8,041	8,569	-0,5279	0,2975	1,055
SPARC	0,091328	9,298	9,465	-0,1668	0,09497	1,039
CEACAM5	0,101931	2,966	4,381	-1,416	0,8337	0,9917
MIA	0,10205	9,77	9,948	-0,1783	0,1051	0,9912
ABL1	0,106329	4,518	5,194	-0,6765	0,4038	0,9733
CD48	0,109856	6,824	7,008	-0,1838	0,1109	0,9592
DLL1	0,113417	10,83	11,06	-0,2269	0,1383	0,9453
S100A11	0,115303	3,681	3,915	-0,2335	0,1431	0,9382
ADAM-TS 15	0,128217	4,214	4,503	-0,2894	0,1839	0,8921
ITGB5	0,140267	7,551	7,846	-0,2951	0,1938	0,853
FCRLB	0,151086	2,178	2,47	-0,2926	0,1975	0,8208
SCAMP3	0,154512	7,023	7,494	-0,471	0,3208	0,811
WFDC2	0,154941	7,158	7,423	-0,2641	0,1801	0,8098
WIF-1	0,170512	5,311	5,551	-0,2404	0,1704	0,7682
TFPI-2	0,176137	7,95	8,274	-0,3241	0,2328	0,7542
TRAIL	0,179218	8,008	8,188	-0,1795	0,1299	0,7466
5'-NT	0,181897	10,57	10,98	-0,411	0,2993	0,7402
MetAP 2	0,188828	3,549	3,934	-0,3845	0,2846	0,7239
ERBB2	0,192711	5,649	5,813	-0,1645	0,1228	0,7151
LY9	0,193773	5,753	5,939	-0,1859	0,1392	0,7127
LYN	0,203313	2,628	2,794	-0,1668	0,1277	0,6918
Gal-1	0,211885	7,003	7,104	-0,1009	0,07875	0,6739
IL6	0,21657	4,232	4,91	-0,6777	0,5345	0,6644
CEACAM1	0,219568	8,354	8,425	-0,0711	0,05646	0,6584
MAD homolog 5	0,222398	2,401	2,629	-0,2278	0,1821	0,6529

VEGFR-2	0,240357	6,961	7,052	-0,0914	0,07599	0,6191
FR-alpha	0,242561	9,099	9,312	-0,2132	0,1781	0,6152
PODXL	0,276755	4,276	4,422	-0,1459	0,1312	0,5579
CD207	0,307427	2,358	2,615	-0,2567	0,2464	0,5123
TGFR-2	0,31198	7,391	7,557	-0,1656	0,1605	0,5059
VIM	0,316999	3,546	3,878	-0,3321	0,3253	0,4989
MUC-16	0,352997	4,734	5,304	-0,5702	0,6025	0,4522
ICOSLG	0,391681	4,942	5,024	-0,08238	0,0945	0,4071
GPNMB	0,430415	5,648	5,705	-0,0572	0,07137	0,3661
TLR3	0,433786	4,258	4,497	-0,2383	0,2995	0,3627
CTSV	0,456627	0,6224	0,7574	-0,135	0,1785	0,3404
hK11	0,465441	4,917	5,017	-0,1003	0,1352	0,3321
SEZ6L	0,465443	5,156	5,242	-0,08568	0,1156	0,3321
CXCL13	0,506375	7,384	7,594	-0,2103	0,3119	0,2955
hK14	0,517906	5,608	5,461	0,147	0,2241	0,2857
S100A4	0,522219	5,865	5,731	0,1345	0,2072	0,2821
MSLN	0,579108	4,18	4,362	-0,182	0,3239	0,2372
TNFSF13	0,581657	8,94	9,013	-0,07336	0,1314	0,2353
TNFRSF19	0,583154	5,601	5,742	-0,1417	0,2549	0,2342
LYPD3	0,59184	6,022	6,089	-0,06659	0,1226	0,2278
RSPO3	0,593359	6,004	5,902	0,1019	0,1884	0,2267
CRNN	0,610277	4,658	4,443	0,2148	0,4162	0,2145
TNFRSF4	0,610247	6,672	6,78	-0,1079	0,209	0,2145
XPNPEP2	0,618281	8,708	8,868	-0,1598	0,3167	0,2088
KLK13	0,634535	3,267	3,112	0,1556	0,3234	0,1975
FASLG	0,669106	8,781	8,86	-0,07964	0,1842	0,1745
PPY	0,6839	7,919	7,49	0,4282	1,04	0,165
CPE	0,687394	4,156	4,092	0,06363	0,1563	0,1628
ERBB4	0,692071	10,44	10,49	-0,05133	0,1281	0,1598
TCL1A	0,74387	5,354	5,567	-0,2135	0,6461	0,1285
GPC1	0,764011	4,508	4,547	-0,0385	0,1269	0,1169
FR-gamma	0,765104	7,974	8,214	-0,2404	0,796	0,1163
GZMB	0,776233	4,445	4,337	0,1078	0,3753	0,11
CXL17	0,804728	5,548	5,617	-0,06946	0,278	0,09435

ESM-1	0,823173	10,42	10,37	0,04978	0,2204	0,08451
FGF-BP1	0,874261	4,526	4,485	0,04043	0,2529	0,05836
CD160	0,915536	5,221	5,201	0,01975	0,1843	0,03832
hK8	0,923081	5,397	5,418	-0,02115	0,2168	0,03476
ITGAV	0,935938	4,527	4,534	-0,006903	0,08503	0,02875
CYR61	0,943468	4,914	4,925	-0,01137	0,1587	0,02527
GZMH	0,978064	5,647	5,635	0,01239	0,4462	0,009633

Supplementary Figure S1: Flow cytometry gating strategy

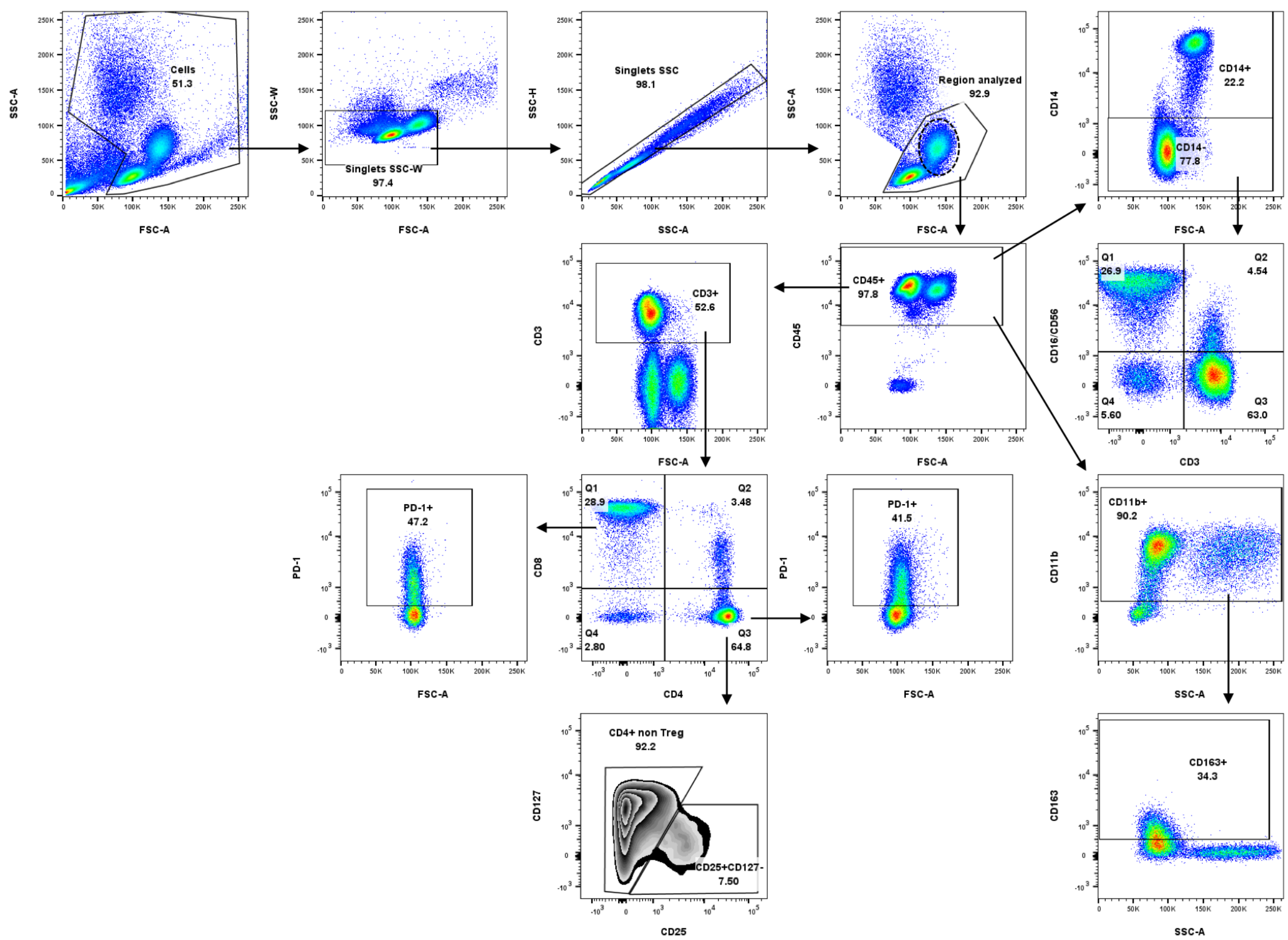

Supplementary Figure S1. Cells were first gated based on forward scatter (FSC)-A vs side scatter (SSC)-A and doublets were removed by gating on FSC-A vs SSW-W and further gating of singlets by gating on SSC-A vs SSC-H. Then the region of interest was selected (mononuclear cells or only monocytes for CD11b+ cells as shown in dashed circle) in FSC-A vs SSC-A. Immune cells were then gated based on CD45 positivity and CD3+, CD14+ cells and CD11b+ were gated out of CD45+. CD11b+ cells were gated for CD163 positivity. Natural killer cells were gated from CD14- cells (CD3-CD56+CD16+). CD8+ and CD4+ T cells were gated from CD3+ cells and further gated for PD-1 positivity. Regulatory T cells (CD25+CD127-) were gated from CD4+ T cells.

Supplementary Figure S2: Proteins associated with immune stimulation and recruitment

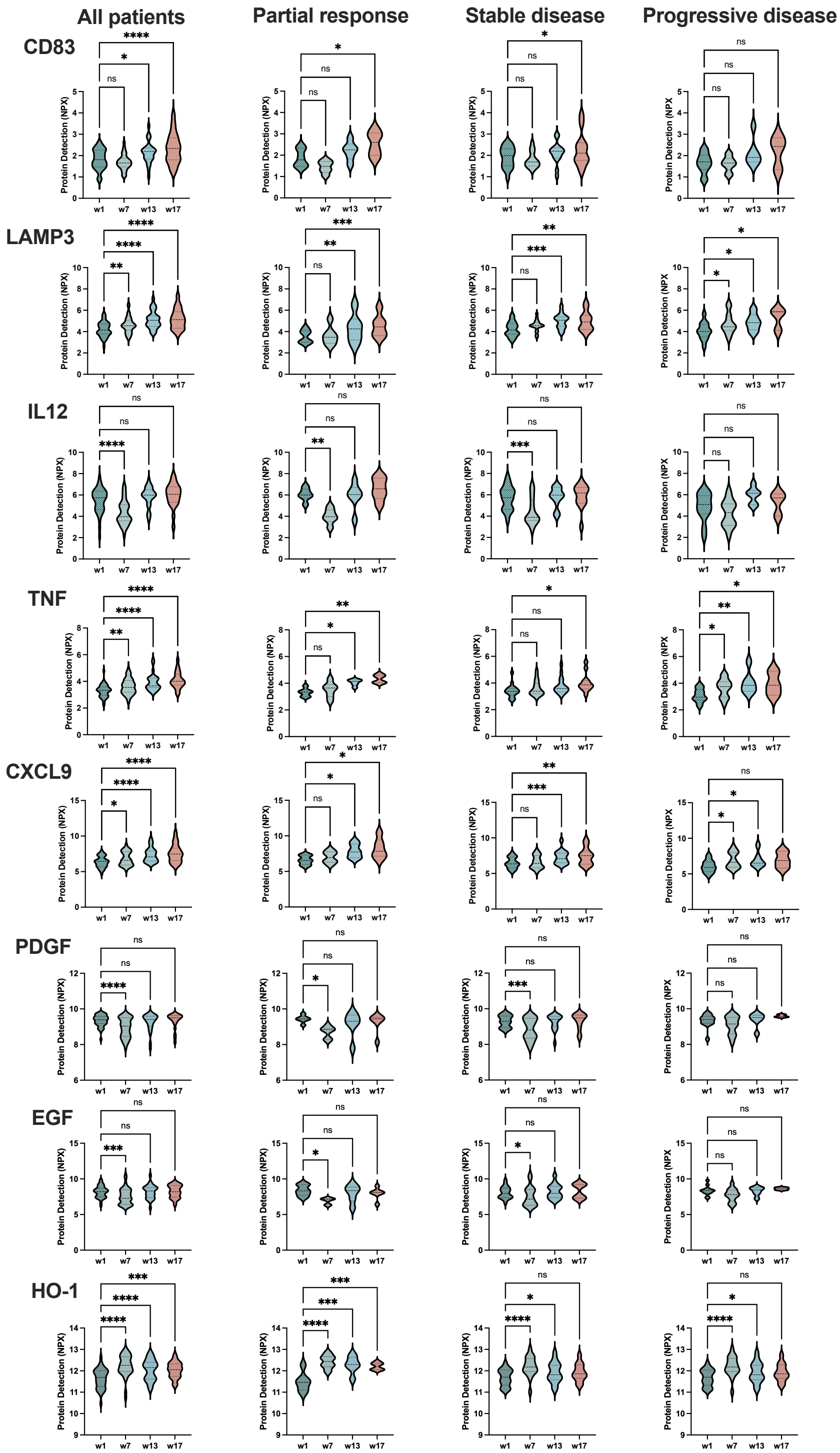

Supplementary Figure S2. Plasma samples from patients in the LOKON002 clinical trial were analyzed with Olink Target 96 Immuno-oncology panel and divided by tumor response. Statistical significances were calculated using a 2-way ANOVA with Dunnett's multiple comparison test.

Supplementary Figure S3: Proteins associated with T and NK cells

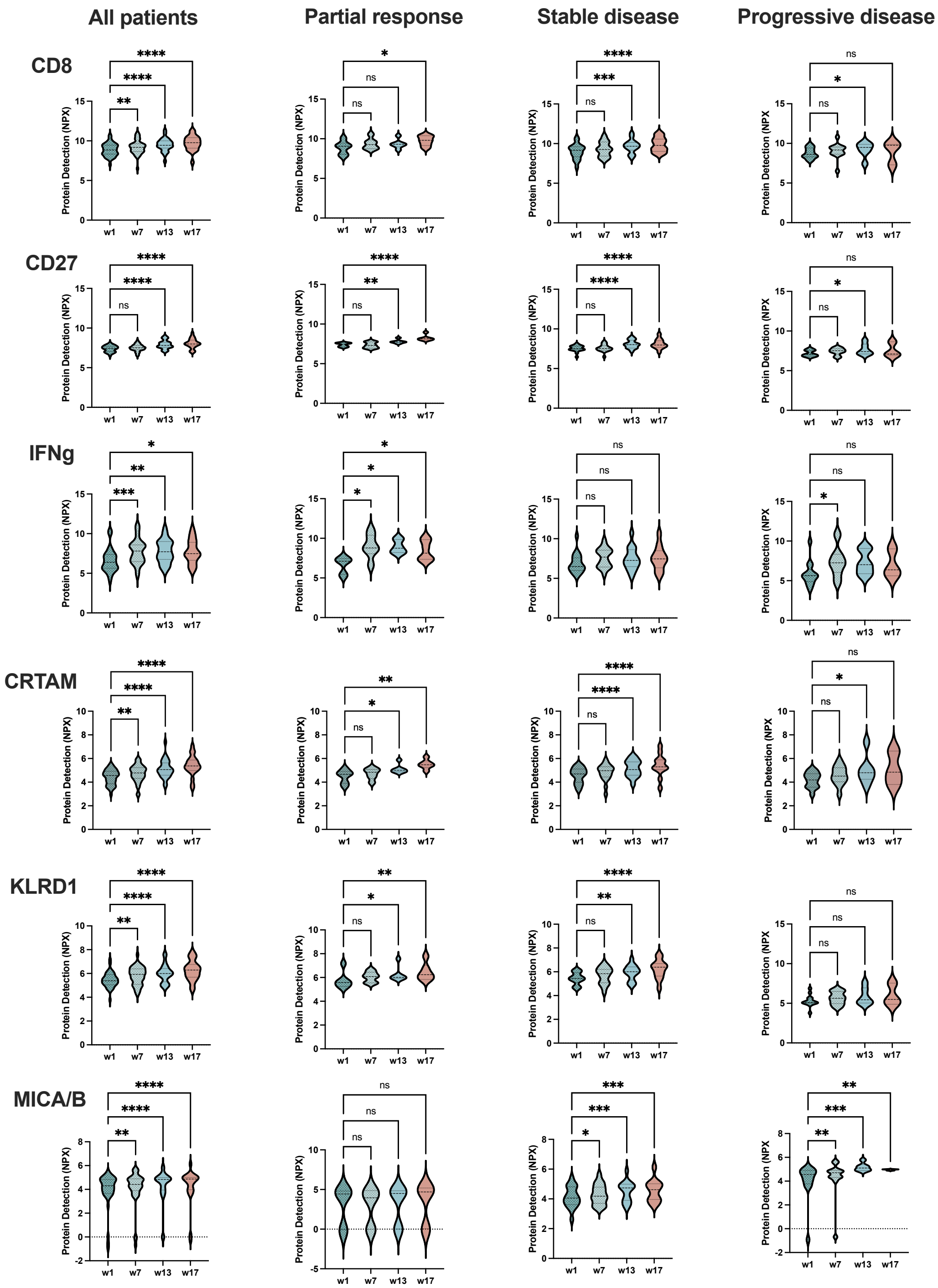

Supplementary Figure S3. Plasma samples from patients in the LOKON002 clinical trial were analyzed with Olink Target 96 Immuno-oncology panel and divided by tumor response. Statistical significances were calculated using a 2-way ANOVA with Dunnett's multiple comparison test.

Supplementary Figure S4: Proteins associated with immunosuppression

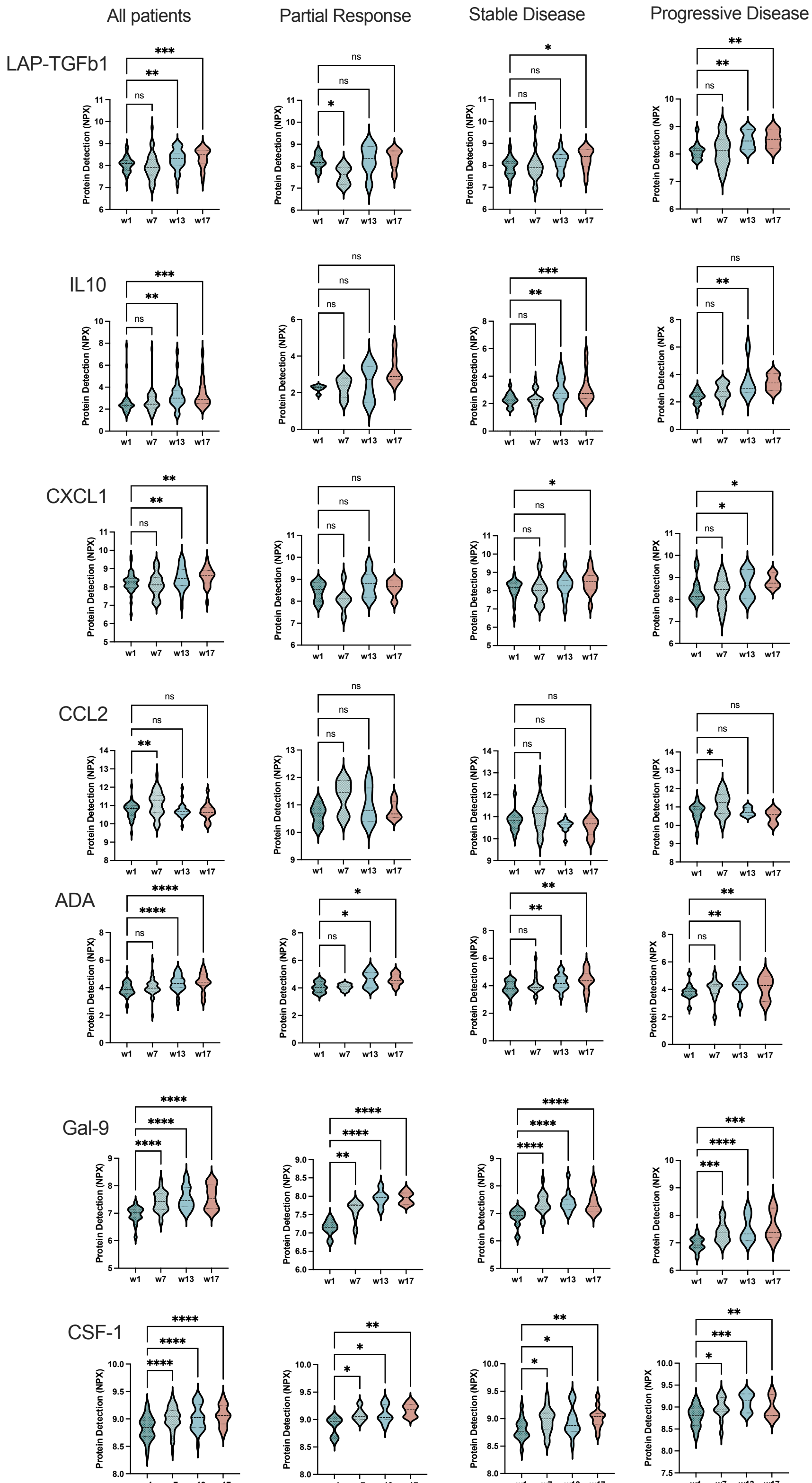

Supplementary Figure S4. Plasma samples from patients in the LOKON002 clinical trial were analyzed with Olink Target 96 Immuno-oncology panel and divided by tumor response. Statistical significances were calculated using a 2-way ANOVA with Dunnett's multiple comparison test

Supplementary Figure S5: Proteins associated with blood vessels and angiogenesis

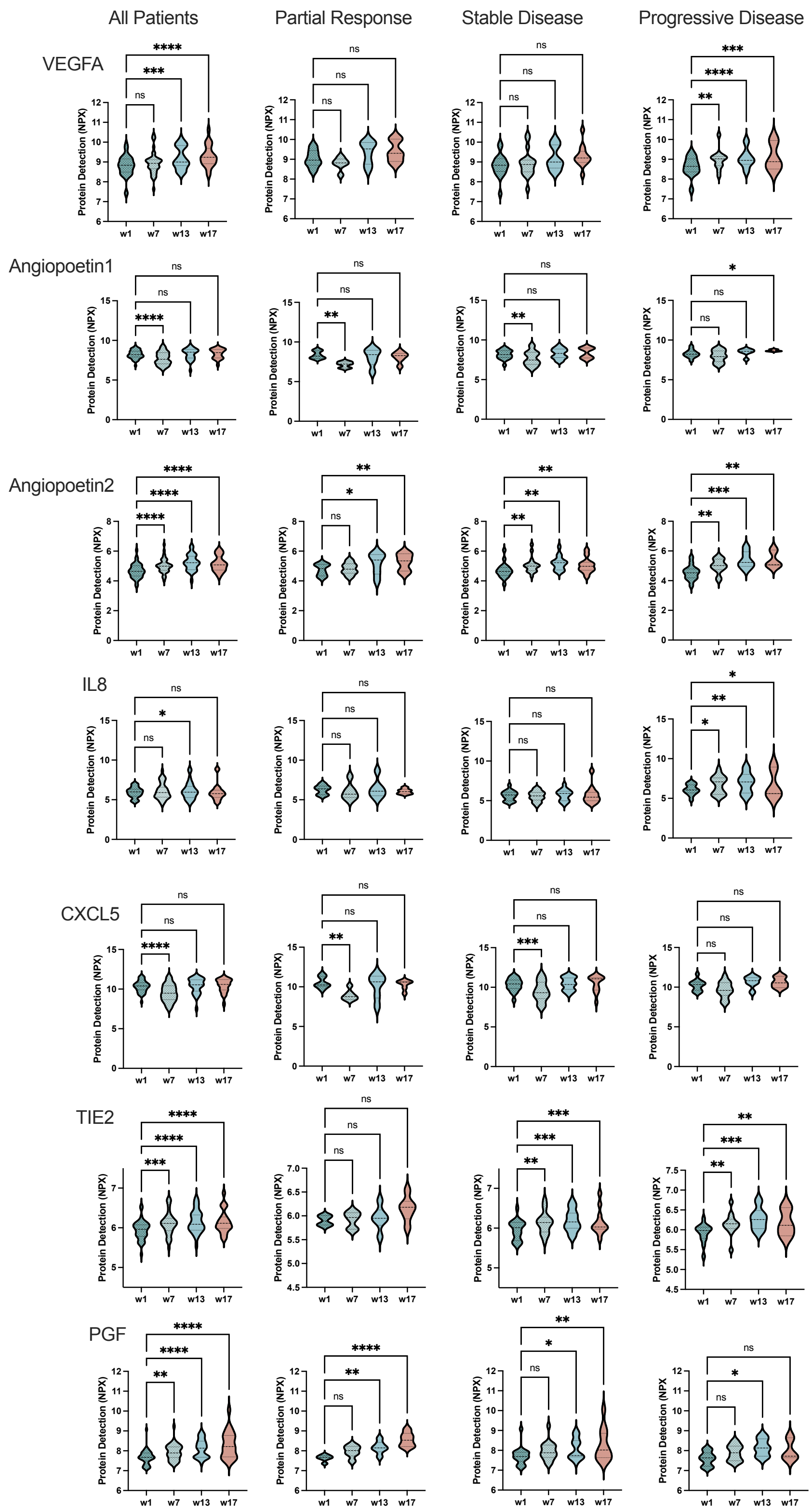

Supplementary Figure S5. Plasma samples from patients in the LOKON002 clinical trial were analyzed with Olink Target 96 Immuno-oncology panel and divided by tumor response. Statistical significances were calculated using a 2-way ANOVA with Dunnett's multiple comparison test.

Supplementary Figure S6: Proteins associated with tumor biology

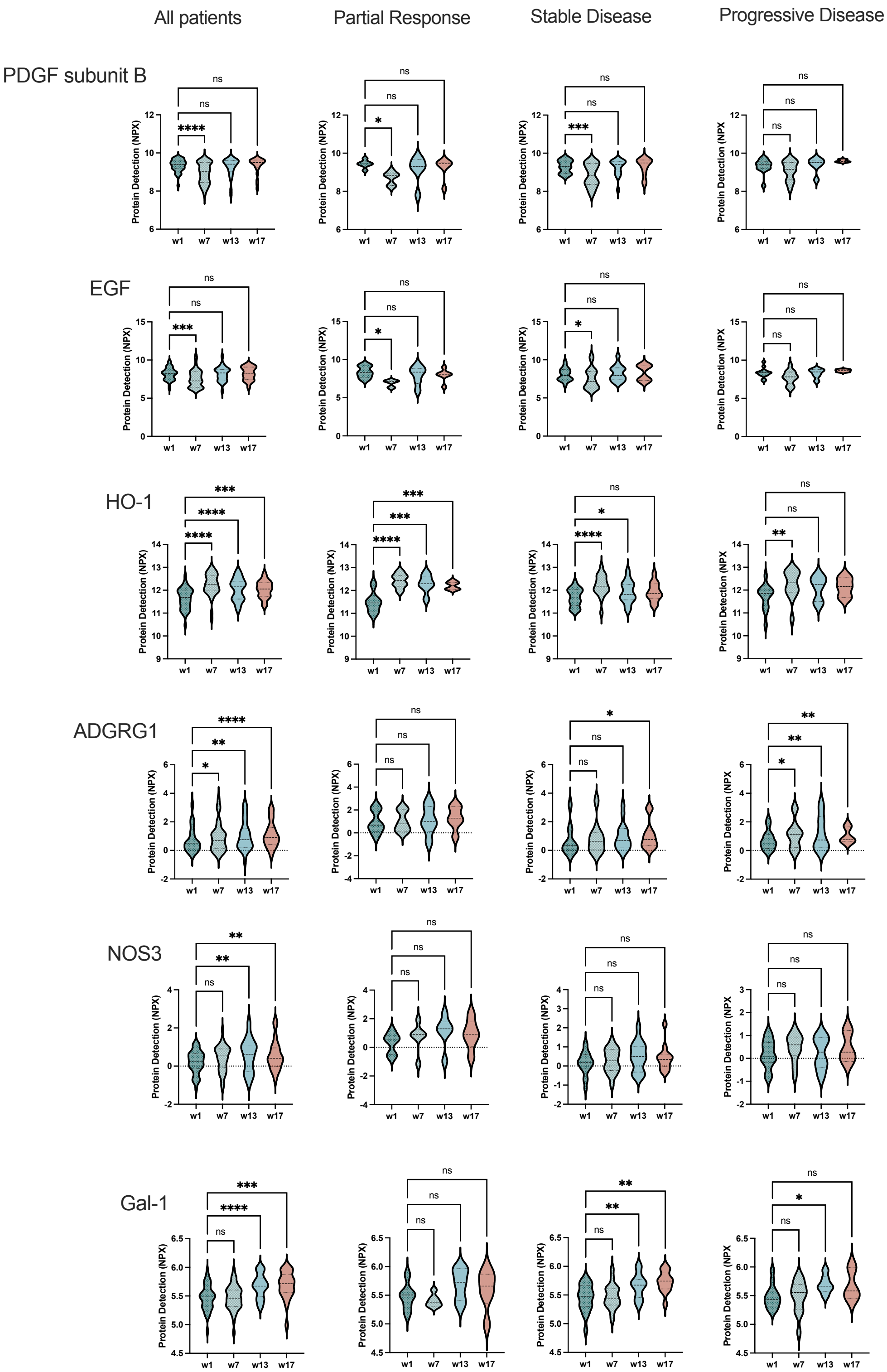

Supplementary Figure S6. Plasma samples from patients in the LOKON002 clinical trial were analyzed with Olink Target 96 Oncology II panel and divided by tumor response. Statistical significances were calculated using a 2-way ANOVA with Dunnett's multiple comparison test.
